# Second generation physical and linkage maps of yellowtail (*Seriola quinqueradiata*) and comparison of synteny with four model fish

**DOI:** 10.1186/s12864-015-1600-7

**Published:** 2015-05-24

**Authors:** Jun-ya Aoki, Wataru Kai, Yumi Kawabata, Akiyuki Ozaki, Kazunori Yoshida, Takashi Koyama, Takashi Sakamoto, Kazuo Araki

**Affiliations:** National Research Institute of Aquaculture, Fisheries Research Agency, 224-1 Hiruta, Tamaki-cho, Watarai-gun, Mie 519-0423 Japan; National Research Institute of Aquaculture, Fisheries Research Agency, 422-1 Nakatsuhamaura, Minamiise-cho, Watarai-gun, Mie 516-0193 Japan; Goto Laboratory, Seikai National Fisheries Research Institute, Fisheries Research Agency, 122-7, Nunoura, Tamanoura-cho, Goto, Nagasaki 853-0508 Japan; Faculty of Marine Science, Tokyo University of Marine Science and Technology, 4-5-7 Konan, Minato-ku, Tokyo 108-8477 Japan

**Keywords:** Yellowtail, Radiation hybrid map, Linkage map, Synteny analysis

## Abstract

**Background:**

Physical and linkage maps are important aids for the assembly of genome sequences, comparative analyses of synteny, and to search for candidate genes by quantitative trait locus analysis. Yellowtail, *Seriola quinqueradiata*, is an economically important species in Japanese aquaculture, and genetic information will be useful for DNA-assisted breeding. We report the construction of a second generation radiation hybrid map, its synteny analysis, and a second generation linkage map containing SNPs (single nucleotide polymorphisms) in yellowtail.

**Results:**

Approximately 1.4 million reads were obtained from transcriptome sequence analysis derived from 11 tissues of one individual. To identify SNPs, cDNA libraries were generated from a pool of 500 whole juveniles, and the gills and kidneys of 100 adults. 9,356 putative SNPs were detected in 6,025 contigs, with a minor allele frequency ≥25%. The linkage and radiation hybrid maps were constructed based on these contig sequences. 2,081 markers, including 601 SNPs markers, were mapped onto the linkage map, and 1,532 markers were mapped in the radiation hybrid map.

**Conclusions:**

The second generation linkage and physical maps were constructed using 6,025 contigs having SNP markers. These maps will aid the *de novo* assembly of sequencing reads, linkage studies and the identification of candidate genes related to important traits. The comparison of marker contigs in the radiation hybrid map indicated that yellowtail is evolutionarily closer to medaka than to green-spotted pufferfish, three-spined stickleback or zebrafish. The synteny analysis may aid studies of chromosomal evolution in yellowtail compared with model fish.

**Electronic supplementary material:**

The online version of this article (doi:10.1186/s12864-015-1600-7) contains supplementary material, which is available to authorized users.

## Background

Teleostei is the most numerous and diversified group of vertebrates. Among them, the order Perciformes is widely distributed throughout the world and is the most diversified of all fish orders. Perciformes are also evolutionarily interesting, because the order includes fish species with various features and forms. However, there is insufficient genomic information for the fishes in this order to allow analyses of chromosomal evolution and the identification of important trait loci for breeding. Yellowtail (*Seriola quinqueradiata*), a member of the order Perciformes, is a popular and important species in Japanese aquaculture. In this study, we report the results of transcriptome analysis, including single nucleotide polymorphism (SNP) identification.

Sequencing of expressed sequence tags (ESTs) derived from cDNA libraries is considered a useful method to identify transcripts in species that lack a sequenced genome [1]. However, obtaining a high-quality EST database using Sanger’s sequencing method is time consuming and expensive. Consequently, this method has been restricted to a few aquaculture fishes, such as Atlantic salmon [2] rainbow trout [3], and catfish [4]. Recently, next generation sequencing, e.g. Illumina sequencing, has been developed and has allowed transcriptome analysis in aquaculture fish species, such as catfish [5], silver carp [6], and large yellow croaker [7], and has permitted the identification of gene-associated markers in catfish [8], carp [9] and rainbow trout [10]. The parallel pyrosequencing technology, commercialized by 454 pyrosequencing, is another next generation sequencing technology and has been used to identify gene-associated SNPs derived from ESTs, e.g. in the blunt snout bream [11], lake whitefish [12], cutthroat trout [13], Atlantic herring [14] and Atlantic cod [15]. We developed this pyrosequencing technology to find SNPs in the transciptomes of yellowtail. SNPs in coding regions are one of the most important DNA variants for quantitative trait locus (QTL) mapping, because some Mendelian and genetically complex traits are caused by SNPs in coding regions [16].

Physical and linkage maps are important aids in the assembly of genome sequences, for comparative analysis of synteny and for identifying candidate genes by QTL analysis. High-resolution linkage maps have been constructed for various fishes, such as medaka, zebrafish, Atlantic salmon [17], rainbow trout [18], Asian seabass [19], catfish [20], Atlantic cod [21], pufferfish (fugu) [22] and carp [23]. In genetic studies of *Seriola*, the first linkage map between *S. quinqueradiata* and *S. lalandi* was produced by Ohara et al. [24]. Recently, the linkage map has been improved by the addition of more simple sequence repeat (SSR) markers, derived from bacterial artificial chromosome (BAC) end sequences [25]. In this study, a second generation linkage map was produced, represented by 24 linkage groups and having 601 SNP markers.

Moreover, we report the construction of a second generation radiation hybrid (RH) map. An RH map is a powerful tool for building a physical map of the whole genome, and can be used to aid the construction of gene orthology relationships through conserved synteny analysis. Seven RH maps have been reported in six teleost fishes, including two maps of zebrafish [26,27], gilthead sea bream [28], medaka [29], European sea bass [30], Nile tilapia [31] and yellowtail [32]. The second generation RH map of yellowtail has more than 1,500 markers and will be useful for genome sequence assembly and comparative analysis of synteny. The release of the complete genome information of the model fish species medaka (*Oryzias latipes*), zebrafish (*Danio rerio*), green-spotted puffierfish (*Tetraodon nigroviridis*), and three-spined stickleback (*Gasterosteus aculeatus*) has accelerated the evolutionary studies. Finally, we performed synteny analysis to compare yellowtail with model fish species.

## Results and discussion

### *De novo* transcriptome assembly

A cDNA library was generated from pooled RNA samples extracted from 11 tissues from a single individual. Sequencing on the Roche/454 GS FLX Titanium platform generated 1,353,405 reads. The CLC Genomic Workbench carried out the *de novo* assembly. After trimming the adapters and filtering out the low-quality and short reads, 1,345,753 high-quality reads were assembled into 56,449 contigs, with 276,945 reads remaining as singletons. The average length of the contigs was 782 bp, and the N_50_ size was 959 bp.

### Gene ontology analysis

**Figure 1:**
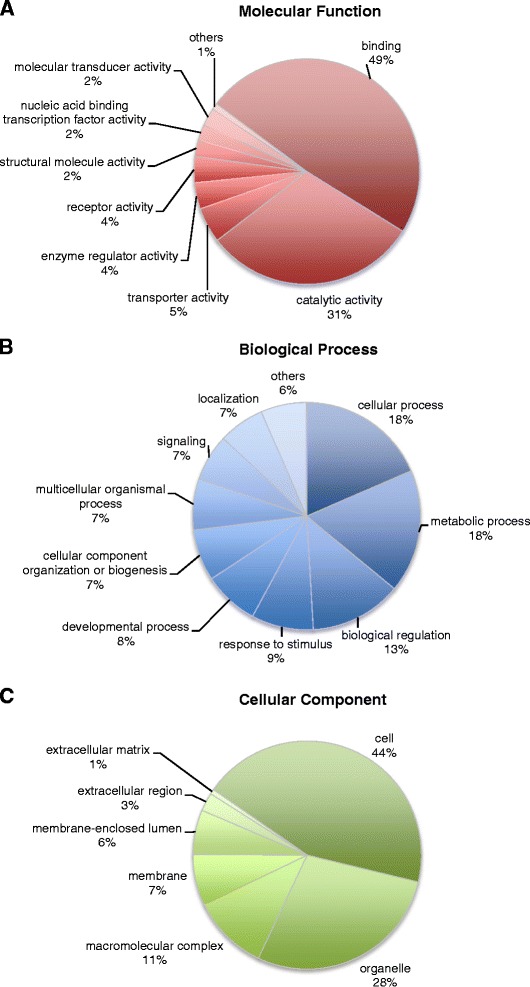
Gene Ontology assignment for assembled contigs. **(A)** Molecular function, **(B)** Biological process and **(C)** Cellular component assignment.

Of 56,449 contigs, 24,035 (43%) had a significant hit and matched 15,280 unique protein records in the nr protein database. Gene ontology (GO) analysis was conducted on these 24,035 contigs. 17,076 sequences were assigned to at least one GO term describing three functional groups: biological process, molecular function and cellular component. Summaries of the level 2 GO assignments are shown in Figure 1. Among the 17,076 sequences, the molecular function ontology comprised the majority of GO assignments (83%), followed by biological processes (79%) and cellular components (74%). In the molecular function category, binding and catalytic activity represented about 80% of the total. For biological processes, sequences involved in cellular processes (19%), metabolic processes (18%), and biological regulation (13%) were highly represented. Finally, the cell and organelle term represented about 70% of the cellular component. Transcriptome assembly using next generation DNA sequencing and GO analysis has been reported in other fish species [33-35]. Our GO analysis of yellowtail transcriptome revealed similar results to those of other fishes.

### SNP identification

Sequencing produced 570,846 raw reads derived from the full-length library and 456,482 raw reads derived from the 3′-anchored library. Quality-based variant calling using the CLC Genomics Workbench detected 9,356 biallelic putative SNPs in 6,025 contigs, with a minor allele frequency (MAF) ≥25%. SNPs with a high allele frequency are more suited for constructing a linkage map efficiently using genotyping of one family because polymorphisms of SNPs decrease in one family. These contigs were registered to DDBJ/EMBL/GenBank as accession number FX884179–FX890203.

### Mapping of SNP markers to the linkage map

**Figure 2:**
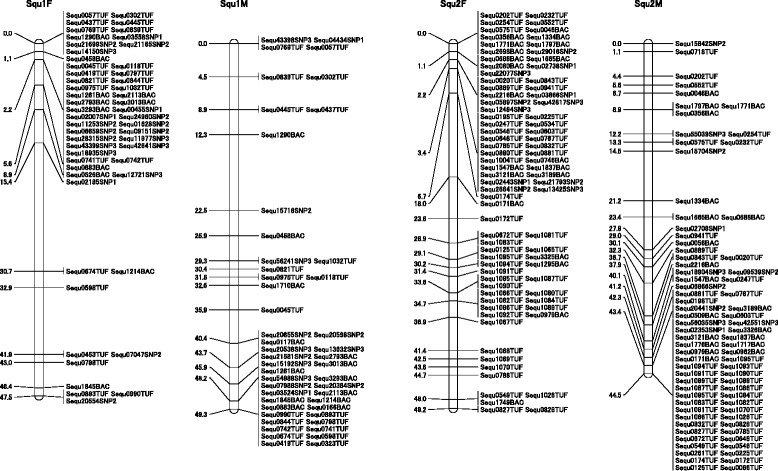
Examples of two linkage groups (female and male). Squ1 has 53 and 48 markers, including 20 and 14 SNPs, in the female and male, respectively. Squ2 has 82 and 77 markers, including 11 and 11 SNPs, in the female and male, respectively. Distances between markers are shown in centiMorgans (cM).

Direct sequencing identified 143 informative SNPs that were heterozygous in either one of the parents, and these heterozygous SNPs were used for linkage analysis using the F_1_ mapping progeny. In the SNPtype assay, 458 SNPs were mapped to the linkage map (Tables 1 and 2, Figure 2, Additional file 1). 2081 markers containing 601 SNPs, which were polymorphic in the F1 mapping progeny, were mapped in the linkage map, and 606 markers were common for both sexes. In this study, SNPs were mapped for the first time in a yellowtail linkage map.

**Table 1:** **Summary of the yellowtail genetic linkage map**

	**Female**				**Male**			
**Linkage group**	**Length (cM)**	**Number of all markers**	**Number of SNP markers**	**Number of SSR markers**	**Length (cM)**	**Number of all markers**	**Number of SNP markers**	**Number of SSR markers**
Squ1	47.49	53	20	33	49.28	48	14	34
Squ2	49.16	82	11	71	44.53	77	11	66
Squ3	40.18	38	9	29	52.71	40	11	29
Squ4	47.35	35	7	28	63.16	36	11	25
Squ5	59.55	40	2	38	50.41	52	13	39
Squ6	51.40	44	14	30	42.62	37	14	23
Squ7	50.60	44	15	29	38.24	48	16	32
Squ8	55.14	41	15	26	51.87	37	14	23
Squ9	30.20	47	9	38	52.93	49	13	36
Squ10	40.39	53	16	37	49.61	54	18	36
Squ11	0	18	8	10	54.82	25	13	12
Squ12	43.99	45	18	27	50.96	43	15	28
Squ13	16.71	29	4	25	47.11	35	10	25
Squ13′	7.84	2	1	1	-	-	-	-
Squ14	24.84	41	8	33	45.10	46	12	34
Squ14′	2.22	2	0	2	-	-	-	-
Squ15	36.98	52	17	35	52.74	50	14	36
Squ16	36.17	36	11	25	51.52	39	12	27
Squ17	38.02	28	7	21	50.47	34	11	23
Squ18	45.56	48	12	36	65.96	42	9	33
Squ19	40.56	45	15	30	59.43	41	15	26
Squ20	51.19	47	16	31	44.66	45	14	31
Squ21	44.88	39	10	29	54.78	36	9	27
Squ22	45.91	33	8	25	53.08	37	12	25
Squ23	41.05	38	13	25	44.05	41	15	26
Squ24	81.86	59	22	37	57.91	50	17	33
Total	1029.24	1039	288	751	1227.95	1042	313	729

**Table 2:** **Summary of the markers of the linkage map**

Total genetic markers	2081
Total mapped SSR markers	1480
Total mapped SNP markers	601
SNPs identified by direct sequencing	143
SNPs identified by SNPtype assay	458

In our genotyping analysis, many polymorphisms in wild yellowtail were observed; however, using one family decreased the SNP frequency to about 10%. Moreover, we used a nanofluidic dynamic array to perform high-throughput genotyping against targeted SNPs. We considered that the nanofluidic dynamic array was useful to genotype SNPs in one family, as well as sequencing analysis by Sanger’s method.

### Construction of the RH map

**Figure 3:**
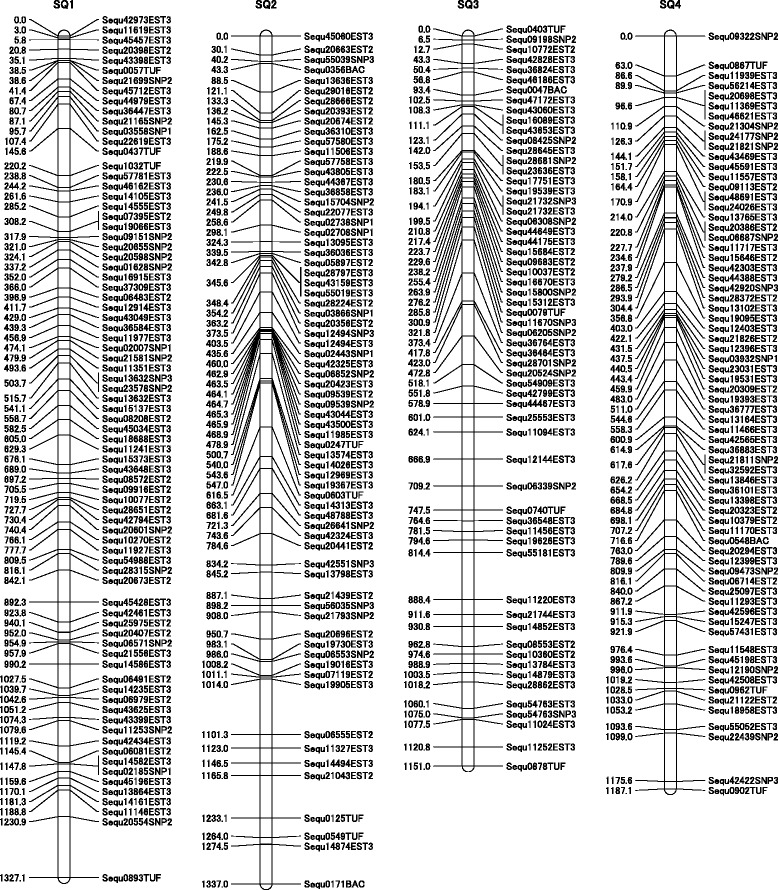
Examples of four radiation hybrid (RH) groups. Each group has 79 markers in SQ1, 70 markers in SQ2, 60 markers in SQ3 and 69 markers in SQ4. Distances between markers are shown in centiRays (cR).

PCR on a dynamic array produces high-throughput gene expression data that are essentially identical in quality to conventional microliter qRT-PCR and are superior to publicly available array data from the same tissue type [36]. In our previous study, 580 markers were mapped in the first RH map [32]. 1,563 markers, containing the previous 580 markers, were used to construct the RH map. The two-point analysis, performed at a LOD score of 4.0 and a distance threshold of 50, resulted in 75 groups using CarthaGene software [37,38]. Furthermore, with reference to the locations of several markers on the constructed genetic linkage map, 1,532 markers (1433 EST markers containing SNPs and 99SSR markers) were distributed to 24 linkage groups (Tables 3 and 4, Figure 3, Additional file 2). Thirty-one markers in eight groups were not distributed among the genetic linkage groups because of their LOD score or a distance threshold error. The RH map was constructed with a final set of 1,532 markers. In each group, the RH map ranged from 640.7 to 1,343.3 centiRays (cR), with an average of approximately 1,096 cR. The combined size of all RH groups was 26,293.5 cR. The estimated size of the yellowtail genome is 800 Mbp [24], which inferred a value of 1 cR = 30 kbp (800 Mbp/26,293.5 cR). We have obtained a large quantity of data in a short time using the BioMark™ HD system since the construction of the first RH map.

**Table 3:** **Summary of the yellowtail radiation hybrid (RH) map**

**Group**	**Size (cR)**	**No. of markers**		
		**Genes**	**SSRs**	**Total**
1	1327.1	75	4	79
2	1337.0	64	6	70
3	1151.0	55	5	60
4	1187.1	65	4	69
5	720.4	42	4	46
6	1139.4	61	4	65
7	982.3	56	3	59
8	968.0	57	4	61
9	1161.1	65	3	68
10	1132.1	60	4	64
11	640.7	33	3	36
12	1233.3	92	9	101
13	1221.8	56	4	60
14	1152.7	51	3	54
15	1343.3	71	4	75
16	1283.6	56	4	60
17	1047.6	63	5	68
18	897.4	52	3	55
19	1287.6	64	4	68
20	1126.1	59	3	62
21	909.8	51	4	55
22	764.0	48	4	52
23	968.1	58	4	62
24	1312.0	79	4	83
Subtotal	26293.5	1433	99	1532
Unlinked		31		31
Total	26293.5	1464	99	1563

cR: centiRay.

**Table 4:** **Summary of the markers of the RH map**

Total genotyped markers	1563
Total mapped markers	1532
Mapped EST markers containing SNPs	1433
Mapped SSR	99
Mapped markers in the previous study [32]	580

The RH map was compared with the linkage map to confirm the accuracy of the local order of markers (Additional file 3). In linkage group 11 of mapped to 0 cM, because chromosome recombination is unlikely to occur during meiosis. It is possible to map these genes accurately on the physical map using RH. The position of some markers was different between the RH and linkage maps, because the physical lengths of the RH map are different from the genetic lengths of the linkage map. In addition, we supposed which region of the RH map is not recombined at meiosis by comparing the RH and the linkage map. The accuracy of the local order of markers will be confirmed when the whole genome sequence becomes available. Currently, we are trying to map genome contigs of yellowtail onto the physical map.

### Synteny relationship with model fish

**Figure 4:**
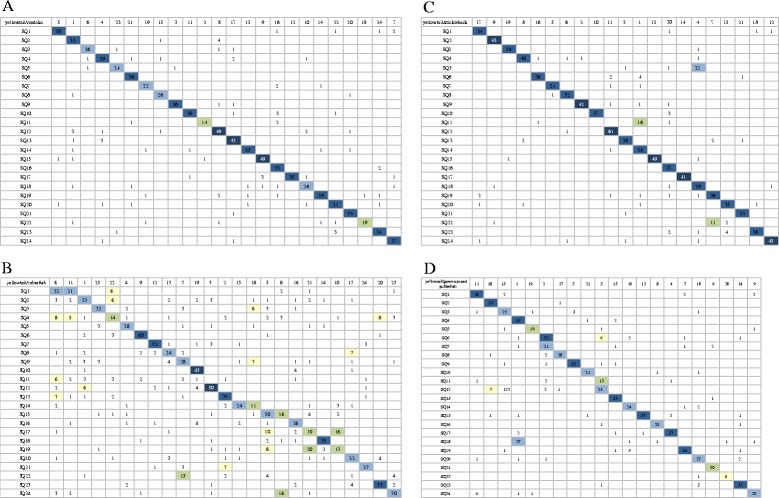
Oxford grids showing conservation of synteny between yellowtail and four model fish. **A**: medaka, **B**: zebrafish, **C**: three-spined stickleback, **D**: green spotted pufferfish. Each box is highlighted as follows: 0–4: white square, 5–10: yellow square, 11–20: green square, 21–30: sky blue square, 31–40: blue square, more than 40: dark blue square.

The 1,433 yellowtail EST marker sequences of the RH map were compared with the cDNA sequences of four fish species: medaka (*Oryzias latipes*), zebrafish (*Danio rerio*), three-spined stickleback (*Gasterosteus aculeatus*) and green-spotted pufferfish (*Tetraodon nigroviridis*) using the TBLASTX algorithm. Among the 1,433 yellowtail marker sequences, 1,036 genes (72.3%) had homologs in medaka, 1,064 genes (74.2%) had homologs in zebrafish, 1,073 genes (74.9%) had homologs in three-spined stickleback, and 1,032 genes (72.0%) had homologs in green-spotted pufferfish (Additional file 4). These values were not significantly different among the four fishes. Oxford grids between yellowtail and the four fish species are shown in Figure 4. The modal number of chromosomes in yellowtail is 48 [32]. This number is the same as that of medaka; all chromosomes could be paired one-to-one between yellowtail and medaka. The number of chromosomes in the two fishes is the same and their chromosomal structures are similar. These results suggested that we would observe conserved synteny between yellowtail and medaka, and that the syntenic relationship between yellowtail and zebrafish would be rather low. However, they might not be evolutionarily closer than the relationships between yellowtail and medaka. Medakas are a member of the order *Beloniformes*, which includes freshwater and marine fish, such as Pacific saury and flying fish. Zebrafish are a member of *Cypriniformes*, which consists exclusively of freshwater fish. Our synteny results reflected the known taxonomic relationships of these fishes. In Teleostei, it is thought that a whole genome duplication and eight subsequent major rearrangements occurred about 314–404 million years ago [39]. Moreover, medaka and zebrafish are thought to have diverged after the eight major rearrangement events, after which medaka and green-spotted pufferfish diverged. The chromosome number in three-spined sticklebacks is the same as that of green-spotted pufferfish. Furthermore, the Oxford grid between yellowtail and green-spotted pufferfish was similar to that between yellowtail and three-spined sticklebacks (Figure 4). However, in the Oxford grid between green-spotted pufferfish and three-spined sticklebacks, the chromosome groups were not paired one-to-one (Additional file 5). Yellowtail SQ11 and 14 correspond to linkage group 1 of three-spined sticklebacks, SQ5 and 18 correspond to linkage group 4, and SQ19 and 22 correspond to linkage group 7, respectively. Then, yellowtail SQ4 and 18 correspond to chromosome 1 of green-spotted pufferfish, SQ6 and 7 correspond to chromosome 2, and SQ11 and 12 correspond to chromosome 3, respectively. These chromosomes in three-spined sticklebacks and green-spotted pufferfish could be paired one-to-two with those of yellowtail. The number of chromosomes in three-spined sticklebacks and green-spotted pufferfish is N=21 and that of yellowtail is N=24. Thus, three-spined sticklebacks and green-spotted pufferfish have three fewer chromosomes than yellowtail; each chromosome is thought to have merged after the divergence from medaka about 191.8 M years ago. By analysis of the whole genome sequence, conserved segments and/or conserved segment orders will be distinguished using the RH map, and will aid studies of the dynamics of chromosome evolution between yellowtail and model fish species.

A putative sex determination locus of yellowtail was located in the Squ12 linkage group [40]; however, its related gene has not been identified. The sex determination gene in fish varies according to fish species: *Dmy* in medaka [41], *Amhy* in Patagonian pejerrey [42], *Amhr2* in fugu [43] and *sdY* in the rainbow trout [44]. Therefore, the identification of sex determination genes is difficult, especially when the conservation of these loci is not high. Detailed chromosome information is provided by analysis of the RH map and whole genome sequences. We are interested in whether yellowtail appeared earlier in evolution than medaka, and when yellowtail diverged from medaka after the major rearrangements of the chromosomes. Currently, we are studying SNPs at the genome level and performing DNA chip analyses using SNPs in ESTs.

## Conclusions

In the transcriptome analysis, 9,356 SNPs were identified in 6,025 contigs. A linkage map for yellowtail was then constructed, which comprised 24 linkage groups including 601 SNP markers, and an RH map was constructed with 1,532 markers. Our synteny results indicated conserved synteny between yellowtail and medaka, and that the syntenic relationship between yellowtail and zebrafish would be rather low: these results reflect the known taxonomic relationships of these fish. The high-density maps will help the assembly of the genome sequence in the comparative analysis of synteny and will aid the search for candidate genes using quantitative trait locus (QTL) analysis.

## Methods

### Ethics statement

Field permits are not required for this species in Japan. The Institute Animal Care and Use Committee of the National Research Institute of Aquaculture (IACUC-NRIA No. 3) approved the fish handling, husbandry and sampling methods.

### Preparation of a cDNA library for transcriptome *de novo* assembly of a single individual

A single adult yellowtail was captured in the Eastern China Sea off Nagasaki prefecture. Total RNAs were extracted from 11 tissues (muscle, brain, heart, liver, intestine, kidney, spleen, gonad, gill, fin and bladder) using RNAiso Plus (Takara, Shiga, Japan). Eurofins MWG Operon (Hamburg, Germany) performed the cDNA library construction and 454-pyrosequencing. After purification of poly (A)^+^ RNA, first-strand cDNA synthesis was primed with an N6 randomized primer. The 454 adapters A and B were ligated to the 5′ and 3′ ends of the cDNA. These fragments were finally amplified with 11 cycles of PCR using a proofreading enzyme. Normalization was carried out by one cycle of denaturation and re-association of the cDNA. The re-associated ds-cDNA was separated from the remaining ss-cDNA by passing the mixture over a hydroxyl apatite column. After hydroxyl apatite chromatography, the ss-cDNA was amplified by seven cycles of PCR. One sequencing run was performed on the Roche/454 GS FLX Titanium platform. All reagents and protocols used were from Roche 454 Life Sciences, USA.

### Preparation of cDNAs for SNP discovery

Full-length and 3′-anchored cDNA libraries were prepared from a pool of 500 whole juveniles, and the gills and kidneys from 100 adult yellowtails. All fish were captured in the East China Sea off Nagasaki prefecture. The full-length cDNA library was constructed using the SMART technology (Takara), with slight modifications. First-strand cDNA synthesis was primed with the SMART-Sfi IA oligonucleotide and the CDS-Sfi IB primer using ReverTra Ace® (Toyobo, Osaka, Japan). The first-strand cDNA was initially amplified for 20 cycles by Long-Distance PCR using Advantage 2 polymerase (Takara). The products were purified using the NucleoSpin purification kit (Macherey-Nagel, Duren, Germany). The cDNA was then normalized with the TRIMMER-DIRECT Kit (Evrogen, Moscow Russia), which uses duplex-specific nuclease treatment. The normalized cDNA samples were diluted and used for PCR amplification with 18 cycles using Advantage 2 polymerase (Clontech, Shiga, Japan). The library was finally purified using a NucleoSpin Extract II (Macherey-Nagel). The 3′-anchored cDNA library was constructed according to the method of Eveland et al. [45]. First-strand cDNA synthesis was primed with 6 pmol of biotinylated (T_12_) B-adaptor modified from Margulies et al. [46]. After purification using a DNAclear Purification kit (Invitrogen, CA, USA), the cDNA was bound to Dynabeads M-270Streptavidin (Invitrogen) and digested with *Msp*I (Promega, WI, USA). An adaptor modified from Margulies et al. [46] was ligated to the restriction site of the cDNA. The template strand was eluted with 10 mM Tris–HCl (pH 8.5) and purified using the QIAquick column (Qiagen, Hilden, Germany). Each cDNA library was sequenced on the Roche/454 GS FLX Titanium platform by Takara Dragon Genomics Center in Japan.

### *De novo* assembly

The raw pyrosequencing data obtained from a cDNA library of a single individual were processed using CLC Genomics Workbench (CLC Bio, Denmark). Adaptor sequences were trimmed, and low-quality reads were filtered out using the following parameters: ambiguous limit of 2; quality limit of 0.05; minimum read length of 50 nt. *De novo* assembly of the processed reads was performed using the following parameters: word size of 22 (automated calculation); bubble size of 445 (automated calculation); mismatch cost of 2; insertion cost of 3; deletion cost of 3; length fraction of 0.8; and similarity of 0.8.

### Gene ontology analysis

Functional annotation of the assembled contigs was performed by BLASTx searching against the NCBI non-redundant (nr) protein database, using the Blast2GO suite [47] with a cutoff *E-*value of *e*^−6^. Gene ontology (GO) analysis of the assembled contigs provides an ontology of defined terms representing gene product properties. The ontology covers three functional groups: biological process, molecular function, and cellular component. To examine broad-level classifications of gene functions, we mapped the GO terms to the Generic GO-Slim database using the Blast2GO suite.

### SNPs discovery

The CLC Genomics Workbench (CLC Bio) was used to analyze the sequencing results of the cDNA libraries. CLC bio’s *de novo* assembly algorithm uses de Bruijn graphs to represent overlapping reads, which is a common approach for short read *de novo* assembly that allows a large number of reads to be handled efficiently [48-50]. Adaptor sequences were trimmed, and low-quality reads were filtered out using the following parameters: ambiguous limit of 2 and a quality limit of 0.05. The processed reads were mapped to the reference sequence generated from the assembled contigs of a single individual yellowtail, using the following parameters: mismatch cost of 2; insertion cost of 3; deletion cost of 3; length fraction of 0.8; similarity of 0.8; and non-specific match handlin=ignore.

SNP detection based on the neighborhood quality standard algorithm [51,52] using the CLC Genomics Workbench was performed using the following parameters: neighborhood radius of 5; maximum gap and mismatch count of 0; minimum neighborhood base quality of 15; minimum central base quality of 20; non-specific match handling = ignore; minimum coverage of 10; minimum allele frequency (MAF) of 5%; and filter homopolymer indels = yes. Only biallelic SNPs were considered.

### Construction of a linkage map

To construct the second generation linkage map, a mapping population was obtained by artificial crossing, as described previously [25]. Direct sequencing of the PCR products and the SNPtype assay of BioMark™ HD system in Fluidigm (USA) were used to perform SNP genotyping.

For direct sequencing, Primer 3 software [53] was used to design primers flanking the SNP sites. PCR amplification was generated using Hot-Start Gene *Taq* (Nippon gene, Tokyo, Japan), and performed over 40 cycles: 15 sec at 95°C, 10 sec at 59°C, 40 sec at 72°C, in addition to a 5-min predenaturation at 95°C and a 3-min postcyle incubation at 72°C. The obtained PCR products of the parents and two offspring of one yellowtail family were sequenced directly using the ABI3130xl Genetic Analyzer (Applied Biosystems, CA, USA) or ABI3730xl DNA Analyzer (Applied Biosystems).

In the SNPtype assay, SNP genotyping of the parents and 94 offspring of one yellowtail family was carried out according to the protocol of the manufacturer. Specific-Target Amplification (STA) primers and Locus-Specific Primers (LSP) for each SNP locus were pooled, and a pre-amplification was performed using 2× Multiplex PCR Master Mix (Qiagen) with the following PCR protocol: 95°C for 15 min, followed by 14 cycles of 95°C for 15 sec and 60°C for 4 min. Pre-amplified DNA was then diluted 1:100 in DNA suspension buffer and 2.5 μL combined with 2× Fast Probe Master Mix (Biotium, CA, USA), 20× Sample Loading Reagent (Fluidigm), and the reference dye ROX (Invitrogen). In parallel, each SNPtype assay mix included allele-specific primers (ASP1 and 2) and LSP was mixed with 2× Assay Loading Reagent. These were then loaded onto 96.96 Dynamic Arrays IFC (Fluidigm), which uses nanofluidic circuitry to combine 96 loci with 96 samples in 9,216 reaction chambers. Thermo-cycling and fluorescence detection were conducted on the BioMark™ HD system (Fluidigm). Finally, linkage analysis was performed as described previously [25]. Linkage analysis was performed using genotype data converted to a backcross format. As the grandparent genotypes were unknown, pairwise analyses were performed, and markers were sorted into linkage groups at a minimum LOD score of 4.0. A goodness-of-fit for Mendelian segregation distortion was tested for all alleles using the chi-square test (p < 0.05, d.f. = 1). Finally, the marker order was determined and double recombination events were checked with MapManagerQTX version 2.0 [54]. The resultant genetic map was visualized using MapChart version 2.2 [55].

### Construction of an RH map

The RH panel, comprising 93 hybrid RH cell lines, and positive and negative controls, was published previously [32]; their 96 DNA samples were extracted. Genotyping reactions were carried out on the Fluidigm platform using a BioMark™ 96.96 nanofluidic dynamic array (Fluidigm) for gene expression analysis, as described previously [32].

The CarthaGene software [37,38] was used to perform two-point linkage analyses and to determine the marker order and inter-marker distances in cR. CarthaGene looks for multiple populations’ maximum likelihood consensus maps using a fast EM algorithm for maximum likelihood estimation and powerful ordering algorithms. The group command at a LOD threshold of 4.0 and a distance threshold of 50 determined the linkage groups, and referenced the linkage map of yellowtail.

### Synteny analysis

EST alignments of mapped markers in the RH map were performed using TBLASTX searches against the cDNA database of zebrafish (*Danio rerio*), medaka (*Oryzias latipes*), three-spined stickleback (*Gasterosteus aculeatus*) and green-spotted pufferfish (*Tetraodon nigroviridis*) at Ensembl [56]. The sequences with the lowest E-value (<1.0E-5) were adopted in each EST alignment.

## Additional files

Additional file 1:
**Linkage map of yellowtail.** Distances between markers are shown in centiMorgans (cM). Additional file 2:
**RH map of yellowtail.** Distances between markers are shown in centiRays (cR). Additional file 3:
**Comparison of the radiation hybrid (RH) map and the linkage map.** Distances between markers are shown in centiRays (cR) on the RH map and in 10^−1^ centiMorgans (cM) on the linkage map. Additional file 4:
**Synteny analysis between the yellowtail radiation hybrid (RH) map and four model fish.**
Additional file 5:
**Oxford grids showing conservation of synteny in** **green-spotted** **pufferfish and** **three-spined** **stickleback.** (BLASTN: E-value < 1.0E-10) Each box was highlighted as follows: 0–100: White square, more than 100: Blue square.
