# Supplementary material for: Second generation physical and linkage maps of yellowtail (Seriola quinqueradiata) and comparison of synteny with four model fish
Source: BMC Genomics. 2015 May 24;16(1):406. doi: 10.1186/s12864-015-1600-7 (PMC4493941; doi:10.1186/s12864-015-1600-7)
Supplement: Additional file 1: — Linkage map of yellowtail. Distances between markers are shown in centiMorgans (cM). [file 12864_2015_1600_MOESM1_ESM.pdf]

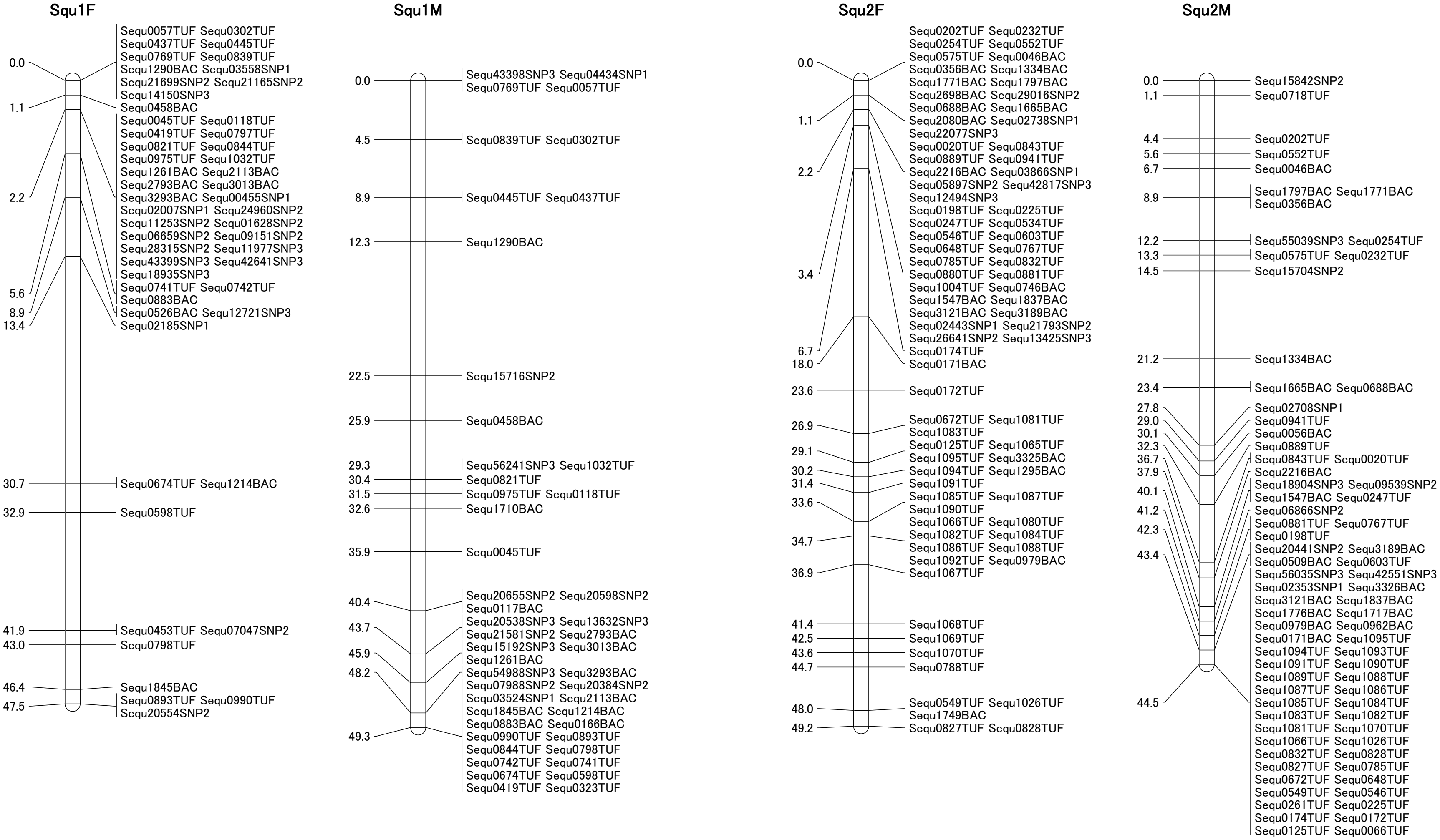
Squ1F
0.0
1.1
2.2
5.6
8.9
13.4
30.7
32.9
41.9
43.0
46.4
47.5
Sequ0057TUF Sequ0302TUF
Sequ0437TUF Sequ0445TUF
Sequ0769TUF Sequ0839TUF
Sequ1290BAC Sequ03558SNP1
Sequ21699SNP2 Sequ21165SNP2
Sequ14150SNP3
Sequ0458BAC
Sequ0045TUF Sequ0118TUF
Sequ0419TUF Sequ0797TUF
Sequ0821TUF Sequ0844TUF
Sequ0975TUF Sequ1032TUF
Sequ1261BAC Sequ2113BAC
Sequ2793BAC Sequ3013BAC
Sequ3293BAC Sequ00455SNP1
Sequ02007SNP1 Sequ24960SNP2
Sequ11253SNP2 Sequ01628SNP2
Sequ06659SNP2 Sequ09151SNP2
Sequ28315SNP2 Sequ11977SNP3
Sequ43399SNP3 Sequ42641SNP3
Sequ18935SNP3
Sequ0741TUF Sequ0742TUF
Sequ0883BAC
Sequ0526BAC Sequ12721SNP3
Sequ02185SNP1
Sequ0674TUF Sequ1214BAC
Sequ0598TUF
Sequ0453TUF Sequ07047SNP2
Sequ0798TUF
Sequ1845BAC
Sequ0893TUF Sequ0990TUF
Sequ20554SNP2
Squ1M
0.0
4.5
8.9
12.3
22.5
25.9
29.3
30.4
31.5
32.6
35.9
40.4
43.7
45.9
48.2
49.3
Sequ43398SNP3 Sequ04434SNP1
Sequ0769TUF Sequ0057TUF
Sequ0839TUF Sequ0302TUF
Sequ0445TUF Sequ0437TUF
Sequ1290BAC
Sequ15716SNP2
Sequ0458BAC
Sequ56241SNP3 Sequ1032TUF
Sequ0821TUF
Sequ0975TUF Sequ0118TUF
Sequ1710BAC
Sequ0045TUF
Sequ20655SNP2 Sequ20598SNP2
Sequ0117BAC
Sequ20538SNP3 Sequ13632SNP3
Sequ21581SNP2 Sequ2793BAC
Sequ15192SNP3 Sequ3013BAC
Sequ1261BAC
Sequ54988SNP3 Sequ3293BAC
Sequ07988SNP2 Sequ20384SNP2
Sequ03524SNP1 Sequ2113BAC
Sequ1845BAC Sequ1214BAC
Sequ0883BAC Sequ0166BAC
Sequ0990TUF Sequ0893TUF
Sequ0844TUF Sequ0798TUF
Sequ0742TUF Sequ0741TUF
Sequ0674TUF Sequ0598TUF
Sequ0419TUF Sequ0323TUF
Squ2F
0.0
1.1
2.2
3.4
6.7
18.0
23.6
26.9
29.1
30.2
31.4
33.6
34.7
36.9
41.4
42.5
43.6
44.7
48.0
49.2
Sequ0202TUF Sequ0232TUF
Sequ0254TUF Sequ0552TUF
Sequ0575TUF Sequ0046BAC
Sequ0356BAC Sequ1334BAC
Sequ1771BAC Sequ1797BAC
Sequ2698BAC Sequ29016SNP2
Sequ0688BAC Sequ1665BAC
Sequ2080BAC Sequ02738SNP1
Sequ22077SNP3
Sequ0020TUF Sequ0843TUF
Sequ0889TUF Sequ0941TUF
Sequ2216BAC Sequ03866SNP1
Sequ05897SNP2 Sequ42817SNP3
Sequ12494SNP3
Sequ0198TUF Sequ0225TUF
Sequ0247TUF Sequ0534TUF
Sequ0546TUF Sequ0603TUF
Sequ0648TUF Sequ0767TUF
Sequ0785TUF Sequ0832TUF
Sequ0880TUF Sequ0881TUF
Sequ1004TUF Sequ0746BAC
Sequ1547BAC Sequ1837BAC
Sequ3121BAC Sequ3189BAC
Sequ02443SNP1 Sequ21793SNP2
Sequ26641SNP2 Sequ13425SNP3
Sequ0174TUF
Sequ0171BAC
Sequ0172TUF
Sequ0672TUF Sequ1081TUF
Sequ1083TUF
Sequ0125TUF Sequ1065TUF
Sequ1095TUF Sequ3325BAC
Sequ1094TUF Sequ1295BAC
Sequ1091TUF
Sequ1085TUF Sequ1087TUF
Sequ1090TUF
Sequ1066TUF Sequ1080TUF
Sequ1082TUF Sequ1084TUF
Sequ1086TUF Sequ1088TUF
Sequ1092TUF Sequ0979BAC
Sequ1067TUF
Sequ1068TUF
Sequ1069TUF
Sequ1070TUF
Sequ0788TUF
Sequ0549TUF Sequ1026TUF
Sequ1749BAC
Sequ0827TUF Sequ0828TUF
Squ2M
0.0
1.1
4.4
5.6
6.7
8.9
12.2
13.3
14.5
21.2
23.4
27.8
29.0
30.1
32.3
36.7
37.9
40.1
41.2
42.3
43.4
44.5
Sequ15842SNP2
Sequ0718TUF
Sequ0202TUF
Sequ0552TUF
Sequ0046BAC
Sequ1797BAC Sequ1771BAC
Sequ0356BAC
Sequ55039SNP3 Sequ0254TUF
Sequ0575TUF Sequ0232TUF
Sequ15704SNP2
Sequ1334BAC
Sequ1665BAC Sequ0688BAC
Sequ02708SNP1
Sequ0941TUF
Sequ0056BAC
Sequ0889TUF
Sequ0843TUF Sequ0020TUF
Sequ2216BAC
Sequ18904SNP3 Sequ09539SNP2
Sequ1547BAC Sequ0247TUF
Sequ06866SNP2
Sequ0881TUF Sequ0767TUF
Sequ0198TUF
Sequ20441SNP2 Sequ3189BAC
Sequ0509BAC Sequ0603TUF
Sequ56035SNP3 Sequ42551SNP3
Sequ02353SNP1 Sequ3326BAC
Sequ3121BAC Sequ1837BAC
Sequ1776BAC Sequ1717BAC
Sequ0979BAC Sequ0962BAC
Sequ0171BAC Sequ1095TUF
Sequ1094TUF Sequ1093TUF
Sequ1091TUF Sequ1090TUF
Sequ1089TUF Sequ1088TUF
Sequ1087TUF Sequ1086TUF
Sequ1085TUF Sequ1084TUF
Sequ1083TUF Sequ1082TUF
Sequ1081TUF Sequ1070TUF
Sequ1066TUF Sequ1026TUF
Sequ0832TUF Sequ0828TUF
Sequ0827TUF Sequ0785TUF
Sequ0672TUF Sequ0648TUF
Sequ0549TUF Sequ0546TUF
Sequ0261TUF Sequ0225TUF
Sequ0174TUF Sequ0172TUF
Sequ0125TUF Sequ0066TUF

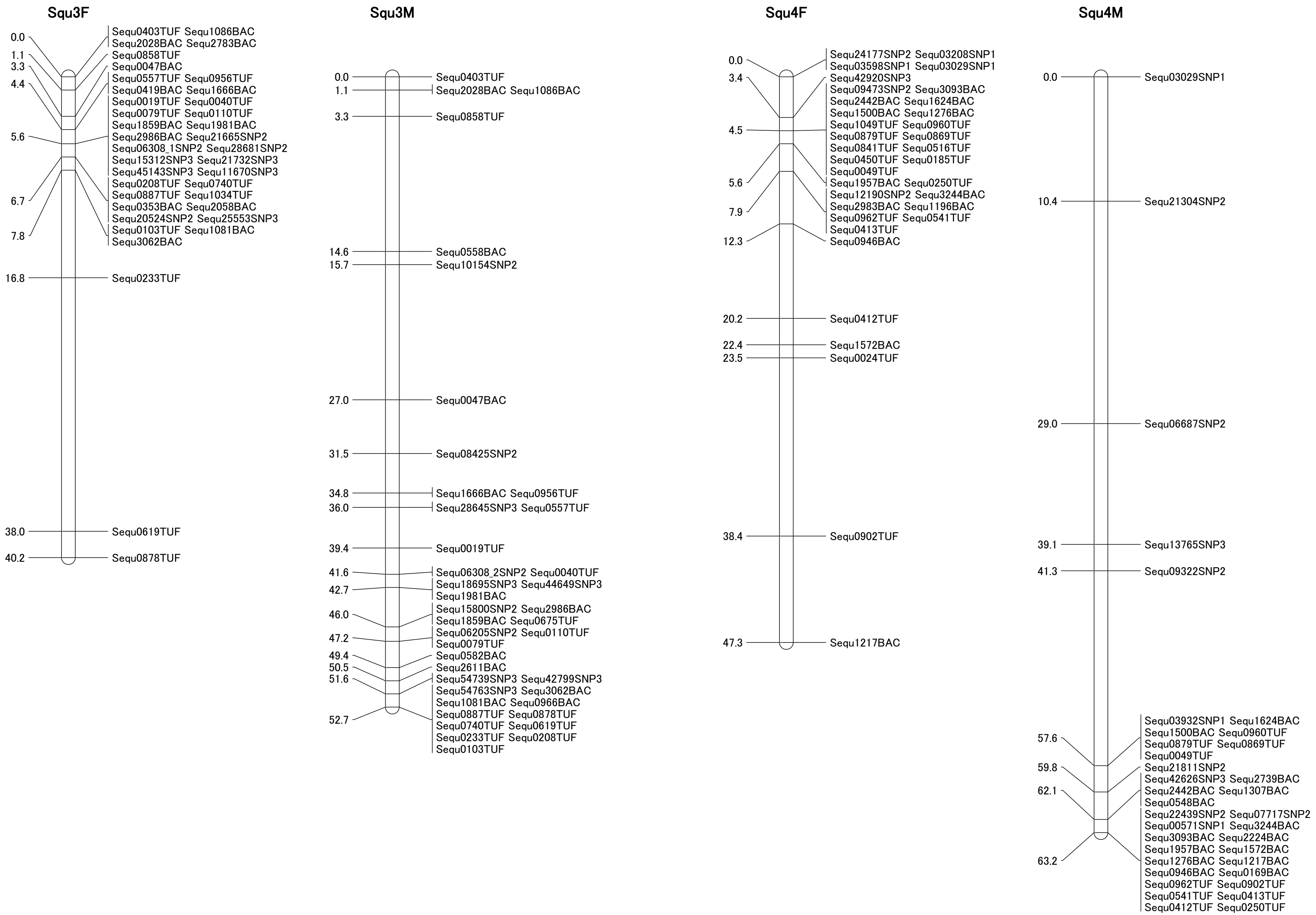

Squ3F
0.0
1.1
3.3
4.4
5.6
6.7
7.8
16.8
38.0
40.2
Sequ0403TUF Sequ1086BAC
Sequ2028BAC Sequ2783BAC
Sequ0858TUF
Sequ0047BAC
Sequ0557TUF Sequ0956TUF
Sequ0419BAC Sequ1666BAC
Sequ0019TUF Sequ0040TUF
Sequ0079TUF Sequ0110TUF
Sequ1859BAC Sequ1981BAC
Sequ2986BAC Sequ21665SNP2
Sequ06308_1SNP2 Sequ28681SNP2
Sequ15312SNP3 Sequ21732SNP3
Sequ45143SNP3 Sequ11670SNP3
Sequ0208TUF Sequ0740TUF
Sequ0887TUF Sequ1034TUF
Sequ0353BAC Sequ2058BAC
Sequ20524SNP2 Sequ25553SNP3
Sequ0103TUF Sequ1081BAC
Sequ3062BAC
Sequ0233TUF
Sequ0619TUF
Sequ0878TUF
Squ3M
0.0 Sequ0403TUF
1.1 Sequ2028BAC Sequ1086BAC
3.3 Sequ0858TUF
14.6 Sequ0558BAC
15.7 Sequ10154SNP2
27.0 Sequ0047BAC
31.5 Sequ08425SNP2
34.8 Sequ1666BAC Sequ0956TUF
36.0 Sequ28645SNP3 Sequ0557TUF
39.4 Sequ0019TUF
41.6 Sequ06308_2SNP2 Sequ0040TUF
42.7 Sequ18695SNP3 Sequ44649SNP3
Sequ1981BAC
46.0 Sequ15800SNP2 Sequ2986BAC
Sequ1859BAC Sequ0675TUF
47.2 Sequ06205SNP2 Sequ0110TUF
Sequ0079TUF
49.4 Sequ0582BAC
50.5 Sequ2611BAC
51.6 Sequ54739SNP3 Sequ42799SNP3
Sequ54763SNP3 Sequ3062BAC
Sequ1081BAC Sequ0966BAC
52.7 Sequ0887TUF Sequ0878TUF
Sequ0740TUF Sequ0619TUF
Sequ0233TUF Sequ0208TUF
Sequ0103TUF
Squ4F
0.0
3.4
4.5
5.6
7.9
12.3
Sequ24177SNP2 Sequ03208SNP1
Sequ03598SNP1 Sequ03029SNP1
Sequ42920SNP3
Sequ09473SNP2 Sequ3093BAC
Sequ2442BAC Sequ1624BAC
Sequ1500BAC Sequ1276BAC
Sequ1049TUF Sequ0960TUF
Sequ0879TUF Sequ0869TUF
Sequ0841TUF Sequ0516TUF
Sequ0450TUF Sequ0185TUF
Sequ0049TUF
Sequ1957BAC Sequ0250TUF
Sequ12190SNP2 Sequ3244BAC
Sequ2983BAC Sequ1196BAC
Sequ0962TUF Sequ0541TUF
Sequ0413TUF
Sequ0946BAC
20.2 Sequ0412TUF
22.4 Sequ1572BAC
23.5 Sequ0024TUF
38.4 Sequ0902TUF
47.3 Sequ1217BAC
Squ4M
0.0 Sequ03029SNP1
10.4 Sequ21304SNP2
29.0 Sequ06687SNP2
39.1 Sequ13765SNP3
41.3 Sequ09322SNP2
57.6
59.8
62.1
63.2
Sequ03932SNP1 Sequ1624BAC
Sequ1500BAC Sequ0960TUF
Sequ0879TUF Sequ0869TUF
Sequ0049TUF
Sequ21811SNP2
Sequ42626SNP3 Sequ2739BAC
Sequ2442BAC Sequ1307BAC
Sequ0548BAC
Sequ22439SNP2 Sequ07717SNP2
Sequ00571SNP1 Sequ3244BAC
Sequ3093BAC Sequ2224BAC
Sequ1957BAC Sequ1572BAC
Sequ1276BAC Sequ1217BAC
Sequ0946BAC Sequ0169BAC
Sequ0962TUF Sequ0902TUF
Sequ0541TUF Sequ0413TUF
Sequ0412TUF Sequ0250TUF

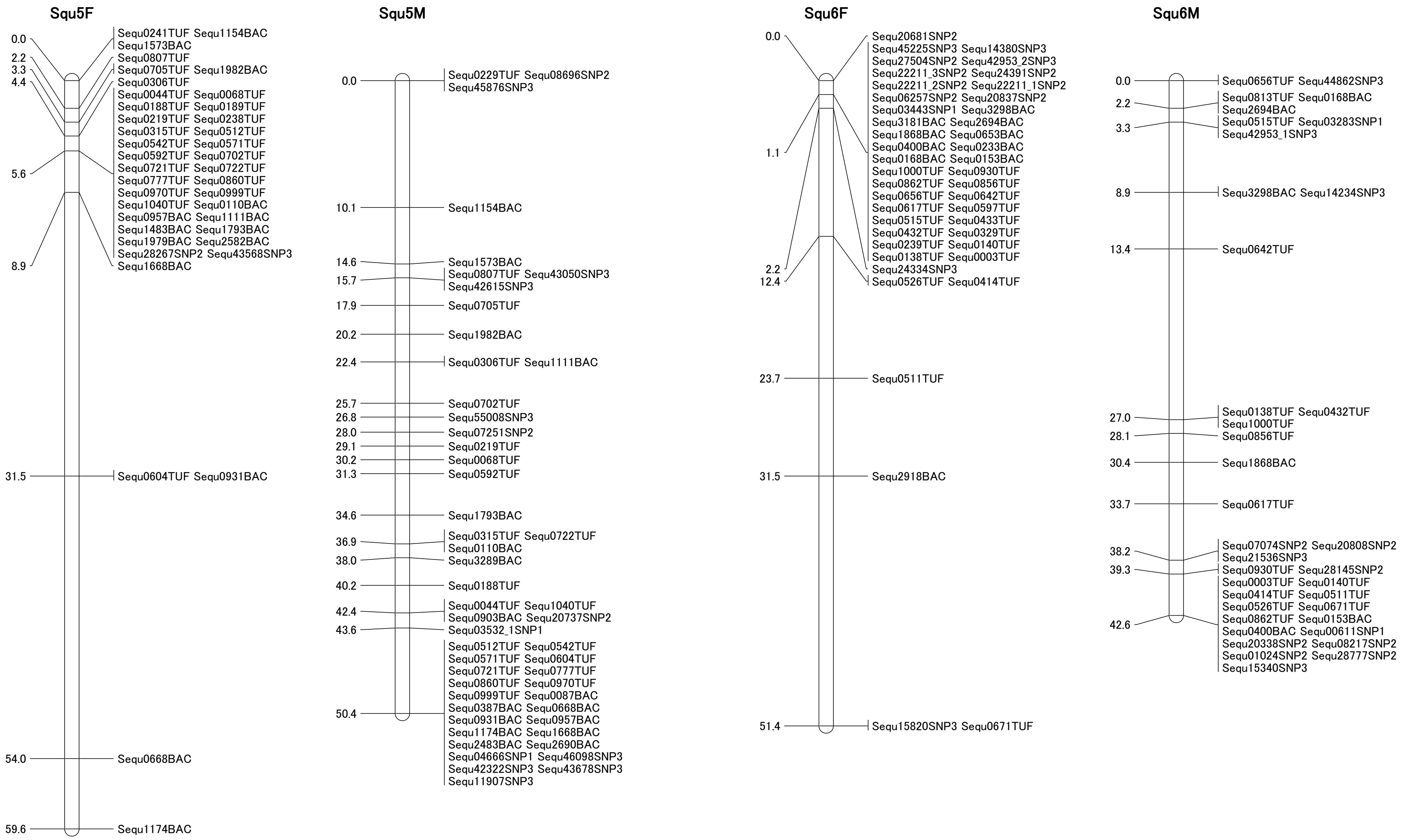
Squ5F
0.0
2.2
3.3
4.4
5.6
8.9
31.5
54.0
59.6
Sequ0241TUF Sequ1154BAC
Sequ1573BAC
Sequ0807TUF
Sequ0705TUF Sequ1982BAC
Sequ0306TUF
Sequ0044TUF Sequ0068TUF
Sequ0188TUF Sequ0189TUF
Sequ0219TUF Sequ0238TUF
Sequ0315TUF Sequ0512TUF
Sequ0542TUF Sequ0571TUF
Sequ0592TUF Sequ0702TUF
Sequ0721TUF Sequ0722TUF
Sequ0777TUF Sequ0860TUF
Sequ0970TUF Sequ0999TUF
Sequ1040TUF Sequ0110BAC
Sequ0957BAC Sequ1111BAC
Sequ1483BAC Sequ1793BAC
Sequ1979BAC Sequ2582BAC
Sequ28267SNP2 Sequ43568SNP3
Sequ1668BAC
Sequ0604TUF Sequ0931BAC
Sequ0668BAC
Sequ1174BAC
Squ5M
0.0
10.1
14.6
15.7
17.9
20.2
22.4
25.7
26.8
28.0
29.1
30.2
31.3
34.6
36.9
38.0
40.2
42.4
43.6
50.4
Sequ0229TUF Sequ08696SNP2
Sequ45876SNP3
Sequ1154BAC
Sequ1573BAC
Sequ0807TUF Sequ43050SNP3
Sequ42615SNP3
Sequ0705TUF
Sequ1982BAC
Sequ0306TUF Sequ1111BAC
Sequ0702TUF
Sequ55008SNP3
Sequ07251SNP2
Sequ0219TUF
Sequ0068TUF
Sequ0592TUF
Sequ1793BAC
Sequ0315TUF Sequ0722TUF
Sequ0110BAC
Sequ3289BAC
Sequ0188TUF
Sequ0044TUF Sequ1040TUF
Sequ0903BAC Sequ20737SNP2
Sequ03532_1SNP1
Sequ0512TUF Sequ0542TUF
Sequ0571TUF Sequ0604TUF
Sequ0721TUF Sequ0777TUF
Sequ0860TUF Sequ0970TUF
Sequ0999TUF Sequ0087BAC
Sequ0387BAC Sequ0668BAC
Sequ0931BAC Sequ0957BAC
Sequ1174BAC Sequ1668BAC
Sequ2483BAC Sequ2690BAC
Sequ04666SNP1 Sequ46098SNP3
Sequ42322SNP3 Sequ43678SNP3
Sequ11907SNP3
Squ6F
0.0
1.1
2.2
12.4
23.7
31.5
51.4
Sequ20681SNP2
Sequ45225SNP3 Sequ14380SNP3
Sequ27504SNP2 Sequ42953_2SNP3
Sequ22211_3SNP2 Sequ24391SNP2
Sequ22211_2SNP2 Sequ22211_1SNP2
Sequ06257SNP2 Sequ20837SNP2
Sequ03443SNP1 Sequ3298BAC
Sequ3181BAC Sequ2694BAC
Sequ1868BAC Sequ0653BAC
Sequ0400BAC Sequ0233BAC
Sequ0168BAC Sequ0153BAC
Sequ1000TUF Sequ0930TUF
Sequ0862TUF Sequ0856TUF
Sequ0656TUF Sequ0642TUF
Sequ0617TUF Sequ0597TUF
Sequ0515TUF Sequ0433TUF
Sequ0432TUF Sequ0329TUF
Sequ0239TUF Sequ0140TUF
Sequ0138TUF Sequ0003TUF
Sequ24334SNP3
Sequ0526TUF Sequ0414TUF
Sequ0511TUF
Sequ2918BAC
Sequ15820SNP3 Sequ0671TUF
Squ6M
0.0
2.2
3.3
8.9
13.4
27.0
28.1
30.4
33.7
38.2
39.3
42.6
Sequ0656TUF Sequ44862SNP3
Sequ0813TUF Sequ0168BAC
Sequ2694BAC
Sequ0515TUF Sequ03283SNP1
Sequ42953_1SNP3
Sequ3298BAC Sequ14234SNP3
Sequ0642TUF
Sequ0138TUF Sequ0432TUF
Sequ1000TUF
Sequ0856TUF
Sequ1868BAC
Sequ0617TUF
Sequ07074SNP2 Sequ20808SNP2
Sequ21536SNP3
Sequ0930TUF Sequ28145SNP2
Sequ0003TUF Sequ0140TUF
Sequ0414TUF Sequ0511TUF
Sequ0526TUF Sequ0671TUF
Sequ0862TUF Sequ0153BAC
Sequ0400BAC Sequ00611SNP1
Sequ20338SNP2 Sequ08217SNP2
Sequ01024SNP2 Sequ28777SNP2
Sequ15340SNP3

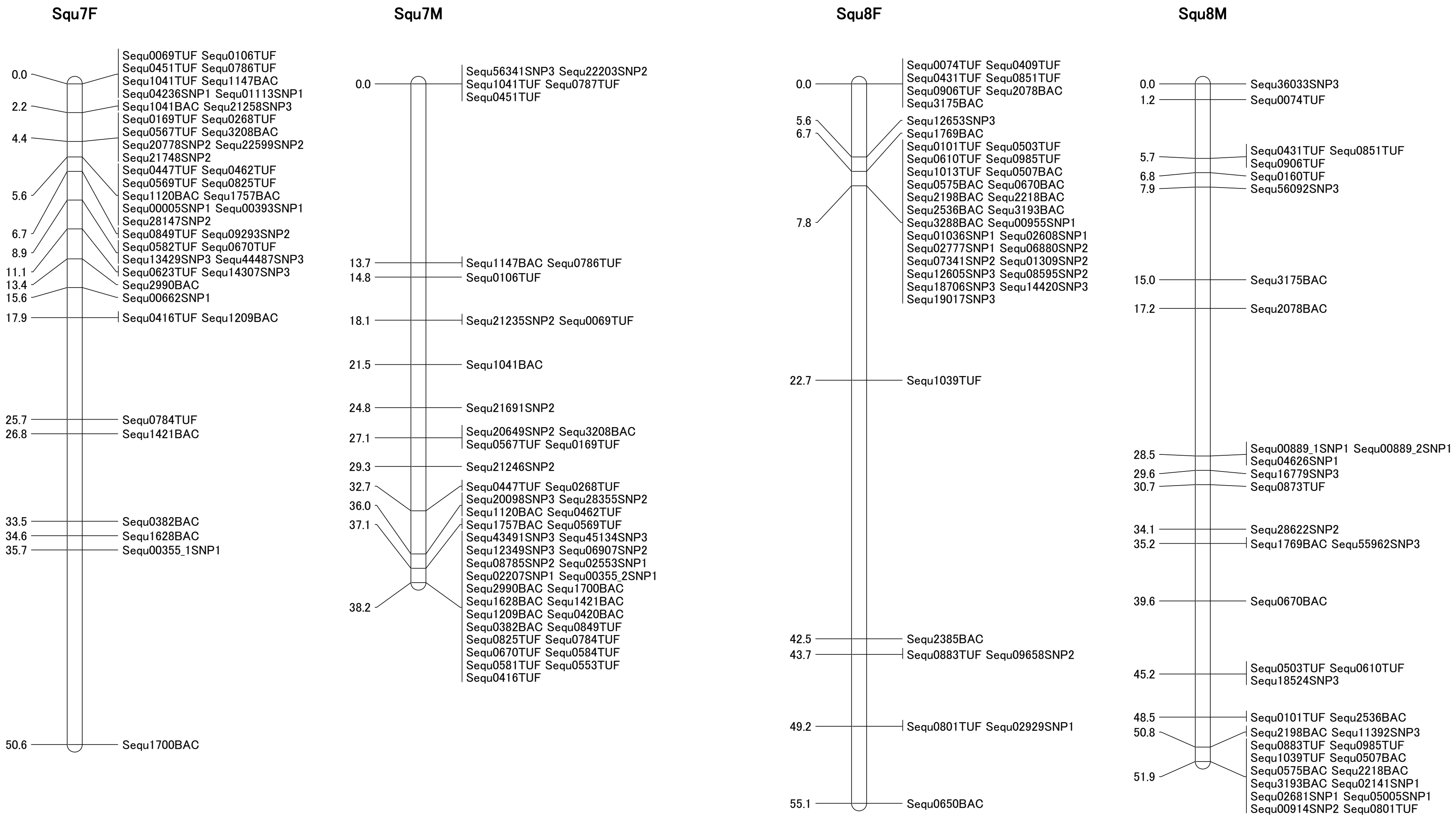
Squ7F
0.0 Sequ0069TUF Sequ0106TUF
Sequ0451TUF Sequ0786TUF
Sequ1041TUF Sequ1147BAC
Sequ04236SNP1 Sequ01113SNP1
2.2 Sequ1041BAC Sequ21258SNP3
Sequ0169TUF Sequ0268TUF
4.4 Sequ0567TUF Sequ3208BAC
Sequ20778SNP2 Sequ22599SNP2
Sequ21748SNP2
Sequ0447TUF Sequ0462TUF
Sequ0569TUF Sequ0825TUF
5.6 Sequ1120BAC Sequ1757BAC
Sequ00005SNP1 Sequ00393SNP1
Sequ28147SNP2
6.7 Sequ0849TUF Sequ09293SNP2
8.9 Sequ0582TUF Sequ0670TUF
Sequ13429SNP3 Sequ44487SNP3
11.1 Sequ0623TUF Sequ14307SNP3
13.4 Sequ2990BAC
15.6 Sequ00662SNP1
17.9 Sequ0416TUF Sequ1209BAC
25.7 Sequ0784TUF
26.8 Sequ1421BAC
33.5 Sequ0382BAC
34.6 Sequ1628BAC
35.7 Sequ00355_1SNP1
50.6 Sequ1700BAC
Squ7M
0.0 Sequ56341SNP3 Sequ22203SNP2
Sequ1041TUF Sequ0787TUF
Sequ0451TUF
13.7 Sequ1147BAC Sequ0786TUF
14.8 Sequ0106TUF
18.1 Sequ21235SNP2 Sequ0069TUF
21.5 Sequ1041BAC
24.8 Sequ21691SNP2
27.1 Sequ20649SNP2 Sequ3208BAC
Sequ0567TUF Sequ0169TUF
29.3 Sequ21246SNP2
32.7 Sequ0447TUF Sequ0268TUF
36.0 Sequ20098SNP3 Sequ28355SNP2
Sequ1120BAC Sequ0462TUF
37.1 Sequ1757BAC Sequ0569TUF
Sequ43491SNP3 Sequ45134SNP3
Sequ12349SNP3 Sequ06907SNP2
Sequ08785SNP2 Sequ02553SNP1
Sequ02207SNP1 Sequ00355_2SNP1
Sequ2990BAC Sequ1700BAC
38.2 Sequ1628BAC Sequ1421BAC
Sequ1209BAC Sequ0420BAC
Sequ0382BAC Sequ0849TUF
Sequ0825TUF Sequ0784TUF
Sequ0670TUF Sequ0584TUF
Sequ0581TUF Sequ0553TUF
Sequ0416TUF
Squ8F
0.0 Sequ0074TUF Sequ0409TUF
Sequ0431TUF Sequ0851TUF
Sequ0906TUF Sequ2078BAC
Sequ3175BAC
5.6 Sequ12653SNP3
6.7 Sequ1769BAC
Sequ0101TUF Sequ0503TUF
Sequ0610TUF Sequ0985TUF
Sequ1013TUF Sequ0507BAC
Sequ0575BAC Sequ0670BAC
Sequ2198BAC Sequ2218BAC
Sequ2536BAC Sequ3193BAC
7.8 Sequ3288BAC Sequ00955SNP1
Sequ01036SNP1 Sequ02608SNP1
Sequ02777SNP1 Sequ06880SNP2
Sequ07341SNP2 Sequ01309SNP2
Sequ12605SNP3 Sequ08595SNP2
Sequ18706SNP3 Sequ14420SNP3
Sequ19017SNP3
22.7 Sequ1039TUF
42.5 Sequ2385BAC
43.7 Sequ0883TUF Sequ09658SNP2
49.2 Sequ0801TUF Sequ02929SNP1
55.1 Sequ0650BAC
Squ8M
0.0 Sequ36033SNP3
1.2 Sequ0074TUF
5.7 Sequ0431TUF Sequ0851TUF
Sequ0906TUF
6.8 Sequ0160TUF
7.9 Sequ56092SNP3
15.0 Sequ3175BAC
17.2 Sequ2078BAC
28.5 Sequ00889_1SNP1 Sequ00889_2SNP1
Sequ04626SNP1
29.6 Sequ16779SNP3
30.7 Sequ0873TUF
34.1 Sequ28622SNP2
35.2 Sequ1769BAC Sequ55962SNP3
39.6 Sequ0670BAC
45.2 Sequ0503TUF Sequ0610TUF
Sequ18524SNP3
48.5 Sequ0101TUF Sequ2536BAC
50.8 Sequ2198BAC Sequ11392SNP3
Sequ0883TUF Sequ0985TUF
Sequ1039TUF Sequ0507BAC
Sequ0575BAC Sequ2218BAC
51.9 Sequ3193BAC Sequ02141SNP1
Sequ02681SNP1 Sequ05005SNP1
Sequ00914SNP2 Sequ0801TUF

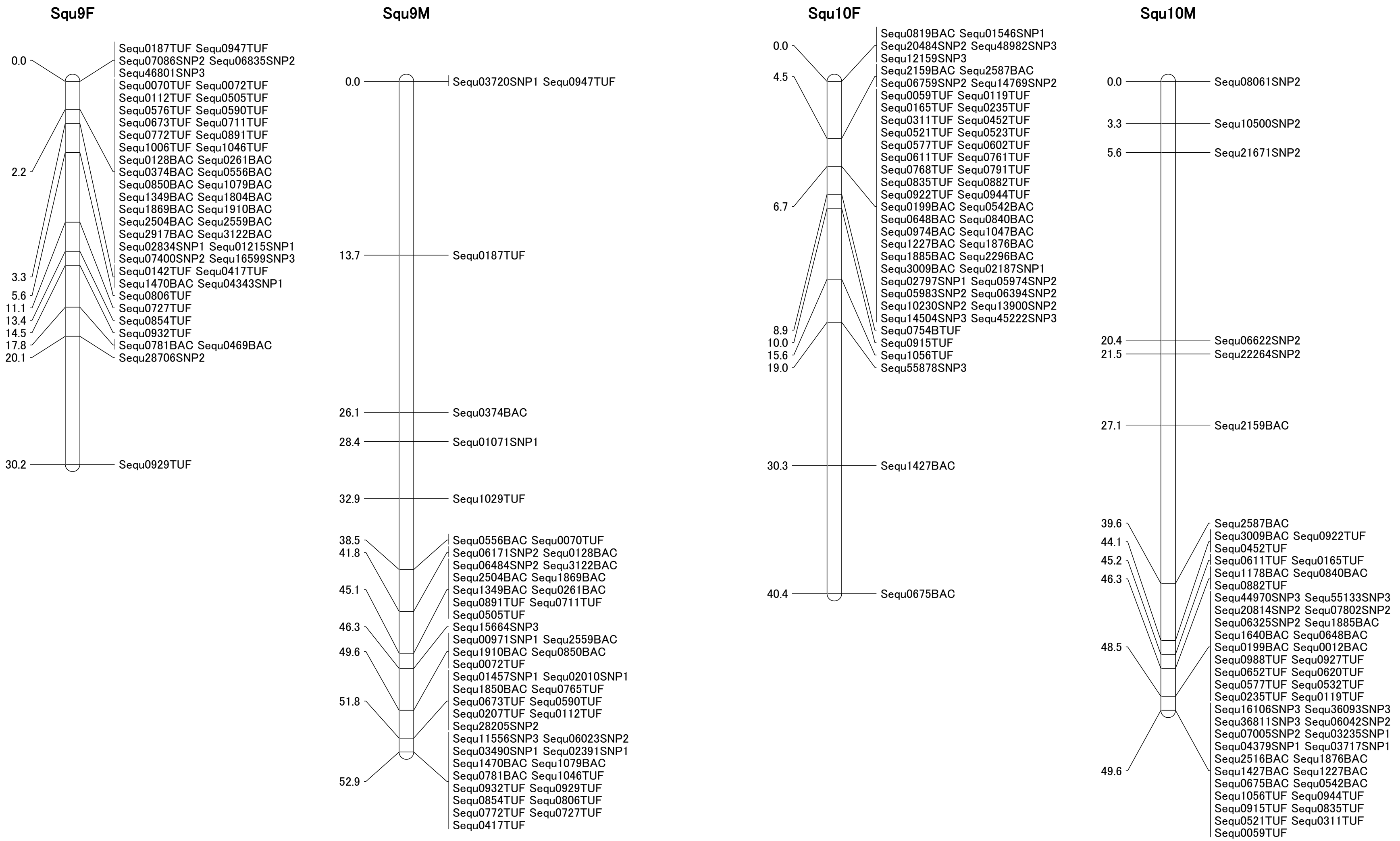
Squ9F
0.0
2.2
3.3
5.6
11.1
13.4
14.5
17.8
20.1
30.2
Sequ0187TUF Sequ0947TUF
Sequ07086SNP2 Sequ06835SNP2
Sequ46801SNP3
Sequ0070TUF Sequ0072TUF
Sequ0112TUF Sequ0505TUF
Sequ0576TUF Sequ0590TUF
Sequ0673TUF Sequ0711TUF
Sequ0772TUF Sequ0891TUF
Sequ1006TUF Sequ1046TUF
Sequ0128BAC Sequ0261BAC
Sequ0374BAC Sequ0556BAC
Sequ0850BAC Sequ1079BAC
Sequ1349BAC Sequ1804BAC
Sequ1869BAC Sequ1910BAC
Sequ2504BAC Sequ2559BAC
Sequ2917BAC Sequ3122BAC
Sequ02834SNP1 Sequ01215SNP1
Sequ07400SNP2 Sequ16599SNP3
Sequ0142TUF Sequ0417TUF
Sequ1470BAC Sequ04343SNP1
Sequ0806TUF
Sequ0727TUF
Sequ0854TUF
Sequ0932TUF
Sequ0781BAC Sequ0469BAC
Sequ28706SNP2
Sequ0929TUF
Squ9M
0.0
13.7
26.1
28.4
32.9
38.5
41.8
45.1
46.3
49.6
51.8
52.9
Sequ03720SNP1 Sequ0947TUF
Sequ0187TUF
Sequ0374BAC
Sequ01071SNP1
Sequ1029TUF
Sequ0556BAC Sequ0070TUF
Sequ06171SNP2 Sequ0128BAC
Sequ06484SNP2 Sequ3122BAC
Sequ2504BAC Sequ1869BAC
Sequ1349BAC Sequ0261BAC
Sequ0891TUF Sequ0711TUF
Sequ0505TUF
Sequ15664SNP3
Sequ00971SNP1 Sequ2559BAC
Sequ1910BAC Sequ0850BAC
Sequ0072TUF
Sequ01457SNP1 Sequ02010SNP1
Sequ1850BAC Sequ0765TUF
Sequ0673TUF Sequ0590TUF
Sequ0207TUF Sequ0112TUF
Sequ28205SNP2
Sequ11556SNP3 Sequ06023SNP2
Sequ03490SNP1 Sequ02391SNP1
Sequ1470BAC Sequ1079BAC
Sequ0781BAC Sequ1046TUF
Sequ0932TUF Sequ0929TUF
Sequ0854TUF Sequ0806TUF
Sequ0772TUF Sequ0727TUF
Sequ0417TUF
Squ10F
0.0
4.5
6.7
8.9
10.0
15.6
19.0
30.3
40.4
Sequ0819BAC Sequ01546SNP1
Sequ20484SNP2 Sequ48982SNP3
Sequ12159SNP3
Sequ2159BAC Sequ2587BAC
Sequ06759SNP2 Sequ14769SNP2
Sequ0059TUF Sequ0119TUF
Sequ0165TUF Sequ0235TUF
Sequ0311TUF Sequ0452TUF
Sequ0521TUF Sequ0523TUF
Sequ0577TUF Sequ0602TUF
Sequ0611TUF Sequ0761TUF
Sequ0768TUF Sequ0791TUF
Sequ0835TUF Sequ0882TUF
Sequ0922TUF Sequ0944TUF
Sequ0199BAC Sequ0542BAC
Sequ0648BAC Sequ0840BAC
Sequ0974BAC Sequ1047BAC
Sequ1227BAC Sequ1876BAC
Sequ1885BAC Sequ2296BAC
Sequ3009BAC Sequ02187SNP1
Sequ02797SNP1 Sequ05974SNP2
Sequ05983SNP2 Sequ06394SNP2
Sequ10230SNP2 Sequ13900SNP2
Sequ14504SNP3 Sequ45222SNP3
Sequ0754BTUF
Sequ0915TUF
Sequ1056TUF
Sequ55878SNP3
Sequ1427BAC
Sequ0675BAC
Squ10M
0.0
3.3
5.6
20.4
21.5
27.1
39.6
44.1
45.2
46.3
48.5
49.6
Sequ08061SNP2
Sequ10500SNP2
Sequ21671SNP2
Sequ06622SNP2
Sequ22264SNP2
Sequ2159BAC
Sequ2587BAC
Sequ3009BAC Sequ0922TUF
Sequ0452TUF
Sequ0611TUF Sequ0165TUF
Sequ1178BAC Sequ0840BAC
Sequ0882TUF
Sequ44970SNP3 Sequ55133SNP3
Sequ20814SNP2 Sequ07802SNP2
Sequ06325SNP2 Sequ1885BAC
Sequ1640BAC Sequ0648BAC
Sequ0199BAC Sequ0012BAC
Sequ0988TUF Sequ0927TUF
Sequ0652TUF Sequ0620TUF
Sequ0577TUF Sequ0532TUF
Sequ0235TUF Sequ0119TUF
Sequ16106SNP3 Sequ36093SNP3
Sequ36811SNP3 Sequ06042SNP2
Sequ07005SNP2 Sequ03235SNP1
Sequ04379SNP1 Sequ03717SNP1
Sequ2516BAC Sequ1876BAC
Sequ1427BAC Sequ1227BAC
Sequ0675BAC Sequ0542BAC
Sequ1056TUF Sequ0944TUF
Sequ0915TUF Sequ0835TUF
Sequ0521TUF Sequ0311TUF
Sequ0059TUF

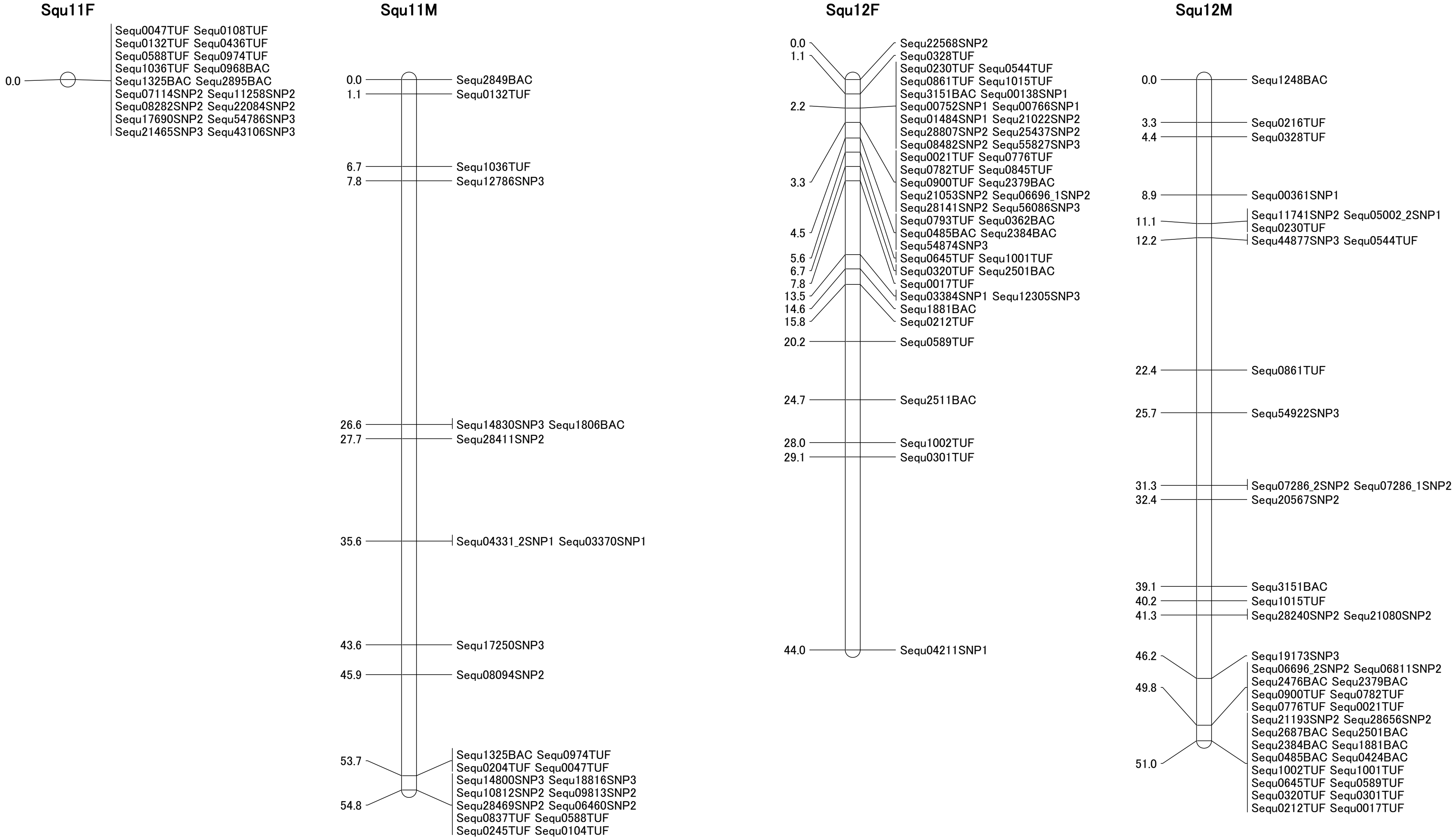
Squ11F
0.0
Sequ0047TUF Sequ0108TUF
Sequ0132TUF Sequ0436TUF
Sequ0588TUF Sequ0974TUF
Sequ1036TUF Sequ0968BAC
Sequ1325BAC Sequ2895BAC
Sequ07114SNP2 Sequ11258SNP2
Sequ08282SNP2 Sequ22084SNP2
Sequ17690SNP2 Sequ54786SNP3
Sequ21465SNP3 Sequ43106SNP3
Squ11M
0.0 Sequ2849BAC
1.1 Sequ0132TUF
6.7 Sequ1036TUF
7.8 Sequ12786SNP3
26.6 Sequ14830SNP3 Sequ1806BAC
27.7 Sequ28411SNP2
35.6 Sequ04331_2SNP1 Sequ03370SNP1
43.6 Sequ17250SNP3
45.9 Sequ08094SNP2
53.7
54.8
Sequ1325BAC Sequ0974TUF
Sequ0204TUF Sequ0047TUF
Sequ14800SNP3 Sequ18816SNP3
Sequ10812SNP2 Sequ09813SNP2
Sequ28469SNP2 Sequ06460SNP2
Sequ0837TUF Sequ0588TUF
Sequ0245TUF Sequ0104TUF
Squ12F
0.0
1.1
2.2
3.3
4.5
5.6
6.7
7.8
13.5
14.6
15.8
Sequ22568SNP2
Sequ0328TUF
Sequ0230TUF Sequ0544TUF
Sequ0861TUF Sequ1015TUF
Sequ3151BAC Sequ00138SNP1
Sequ00752SNP1 Sequ00766SNP1
Sequ01484SNP1 Sequ21022SNP2
Sequ28807SNP2 Sequ25437SNP2
Sequ08482SNP2 Sequ55827SNP3
Sequ0021TUF Sequ0776TUF
Sequ0782TUF Sequ0845TUF
Sequ0900TUF Sequ2379BAC
Sequ21053SNP2 Sequ06696_1SNP2
Sequ28141SNP2 Sequ56086SNP3
Sequ0793TUF Sequ0362BAC
Sequ0485BAC Sequ2384BAC
Sequ54874SNP3
Sequ0645TUF Sequ1001TUF
Sequ0320TUF Sequ2501BAC
Sequ0017TUF
Sequ03384SNP1 Sequ12305SNP3
Sequ1881BAC
Sequ0212TUF
20.2 Sequ0589TUF
24.7 Sequ2511BAC
28.0 Sequ1002TUF
29.1 Sequ0301TUF
44.0 Sequ04211SNP1
Squ12M
0.0 Sequ1248BAC
3.3 Sequ0216TUF
4.4 Sequ0328TUF
8.9 Sequ00361SNP1
11.1 Sequ11741SNP2 Sequ05002_2SNP1
Sequ0230TUF
12.2 Sequ44877SNP3 Sequ0544TUF
22.4 Sequ0861TUF
25.7 Sequ54922SNP3
31.3 Sequ07286_2SNP2 Sequ07286_1SNP2
32.4 Sequ20567SNP2
39.1 Sequ3151BAC
40.2 Sequ1015TUF
41.3 Sequ28240SNP2 Sequ21080SNP2
46.2
49.8
51.0
Sequ19173SNP3
Sequ06696_2SNP2 Sequ06811SNP2
Sequ2476BAC Sequ2379BAC
Sequ0900TUF Sequ0782TUF
Sequ0776TUF Sequ0021TUF
Sequ21193SNP2 Sequ28656SNP2
Sequ2687BAC Sequ2501BAC
Sequ2384BAC Sequ1881BAC
Sequ0485BAC Sequ0424BAC
Sequ1002TUF Sequ1001TUF
Sequ0645TUF Sequ0589TUF
Sequ0320TUF Sequ0301TUF
Sequ0212TUF Sequ0017TUF

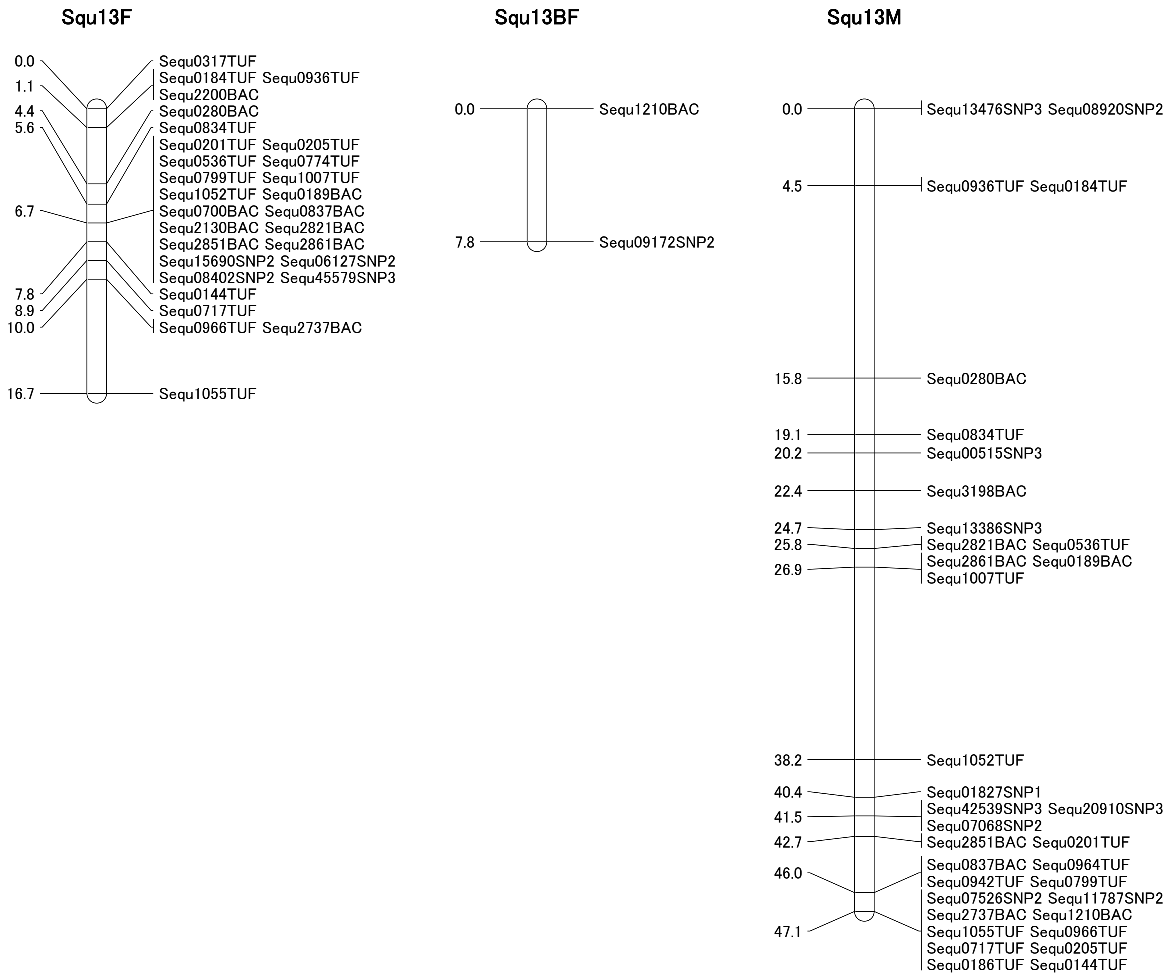

Squ13F
0.0 Sequ0317TUF
1.1 Sequ0184TUF Sequ0936TUF
Sequ2200BAC
4.4 Sequ0280BAC
5.6 Sequ0834TUF
Sequ0201TUF Sequ0205TUF
Sequ0536TUF Sequ0774TUF
Sequ0799TUF Sequ1007TUF
Sequ1052TUF Sequ0189BAC
6.7 Sequ0700BAC Sequ0837BAC
Sequ2130BAC Sequ2821BAC
Sequ2851BAC Sequ2861BAC
Sequ15690SNP2 Sequ06127SNP2
Sequ08402SNP2 Sequ45579SNP3
7.8 Sequ0144TUF
8.9 Sequ0717TUF
10.0 Sequ0966TUF Sequ2737BAC
16.7 Sequ1055TUF
Squ13BF
0.0 Sequ1210BAC
7.8 Sequ09172SNP2
Squ13M
0.0 Sequ13476SNP3 Sequ08920SNP2
4.5 Sequ0936TUF Sequ0184TUF
15.8 Sequ0280BAC
19.1 Sequ0834TUF
20.2 Sequ00515SNP3
22.4 Sequ3198BAC
24.7 Sequ13386SNP3
25.8 Sequ2821BAC Sequ0536TUF
Sequ2861BAC Sequ0189BAC
26.9 Sequ1007TUF
38.2 Sequ1052TUF
40.4 Sequ01827SNP1
Sequ42539SNP3 Sequ20910SNP3
41.5 Sequ07068SNP2
42.7 Sequ2851BAC Sequ0201TUF
Sequ0837BAC Sequ0964TUF
46.0 Sequ0942TUF Sequ0799TUF
Sequ07526SNP2 Sequ11787SNP2
Sequ2737BAC Sequ1210BAC
47.1 Sequ1055TUF Sequ0966TUF
Sequ0717TUF Sequ0205TUF
Sequ0186TUF Sequ0144TUF

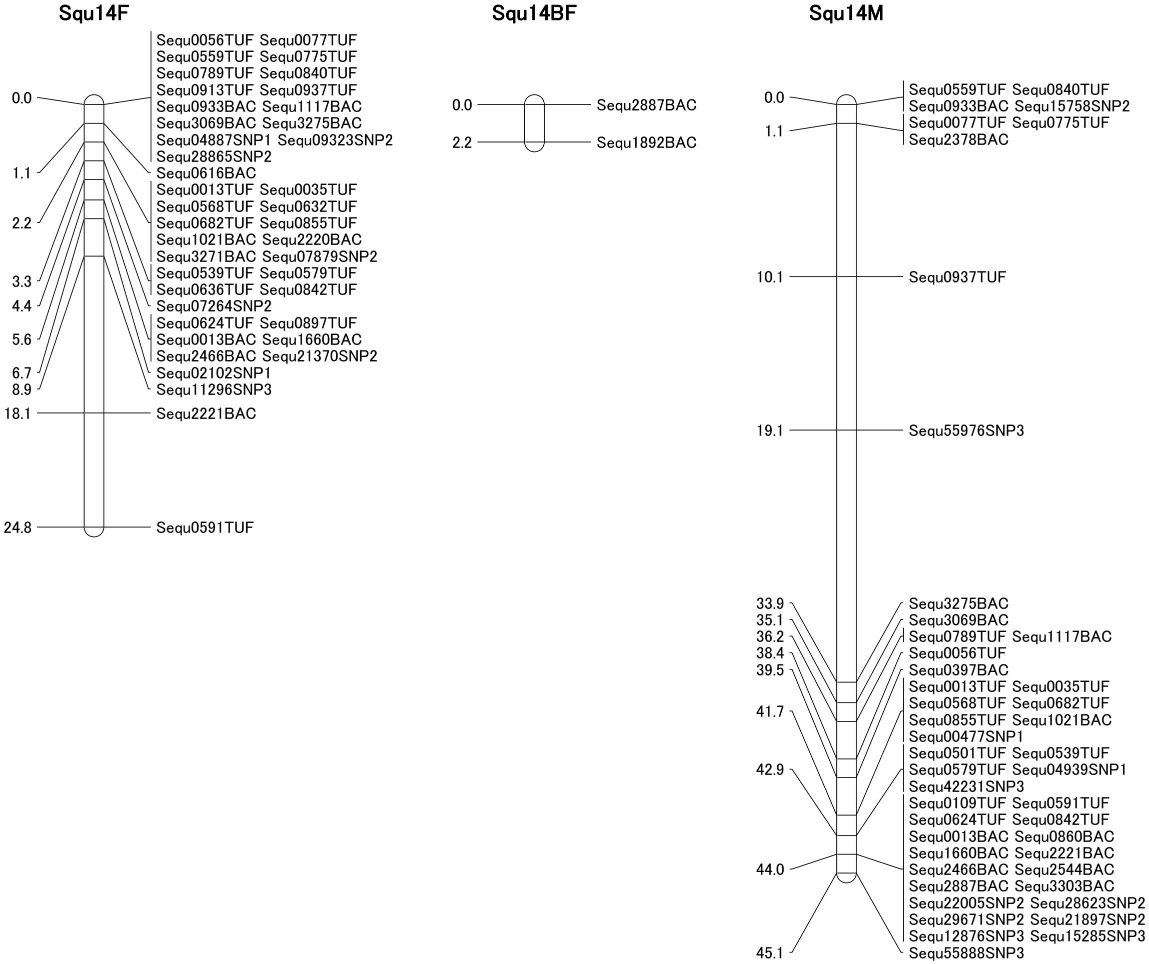
Squ14F
Squ14BF
Squ14M
0.0
1.1
2.2
3.3
4.4
5.6
6.7
8.9
18.1
24.8
Sequ0056TUF Sequ0077TUF
Sequ0559TUF Sequ0775TUF
Sequ0789TUF Sequ0840TUF
Sequ0913TUF Sequ0937TUF
Sequ0933BAC Sequ1117BAC
Sequ3069BAC Sequ3275BAC
Sequ04887SNP1 Sequ09323SNP2
Sequ28865SNP2
Sequ0616BAC
Sequ0013TUF Sequ0035TUF
Sequ0568TUF Sequ0632TUF
Sequ0682TUF Sequ0855TUF
Sequ1021BAC Sequ2220BAC
Sequ3271BAC Sequ07879SNP2
Sequ0539TUF Sequ0579TUF
Sequ0636TUF Sequ0842TUF
Sequ07264SNP2
Sequ0624TUF Sequ0897TUF
Sequ0013BAC Sequ1660BAC
Sequ2466BAC Sequ21370SNP2
Sequ02102SNP1
Sequ11296SNP3
Sequ2221BAC
Sequ0591TUF
0.0
2.2
Sequ2887BAC
Sequ1892BAC
0.0
1.1
10.1
19.1
33.9
35.1
36.2
38.4
39.5
41.7
42.9
44.0
45.1
Sequ0559TUF Sequ0840TUF
Sequ0933BAC Sequ15758SNP2
Sequ0077TUF Sequ0775TUF
Sequ2378BAC
Sequ0937TUF
Sequ55976SNP3
Sequ3275BAC
Sequ3069BAC
Sequ0789TUF Sequ1117BAC
Sequ0056TUF
Sequ0397BAC
Sequ0013TUF Sequ0035TUF
Sequ0568TUF Sequ0682TUF
Sequ0855TUF Sequ1021BAC
Sequ00477SNP1
Sequ0501TUF Sequ0539TUF
Sequ0579TUF Sequ04939SNP1
Sequ42231SNP3
Sequ0109TUF Sequ0591TUF
Sequ0624TUF Sequ0842TUF
Sequ0013BAC Sequ0860BAC
Sequ1660BAC Sequ2221BAC
Sequ2466BAC Sequ2544BAC
Sequ2887BAC Sequ3303BAC
Sequ22005SNP2 Sequ28623SNP2
Sequ29671SNP2 Sequ21897SNP2
Sequ12876SNP3 Sequ15285SNP3
Sequ55888SNP3

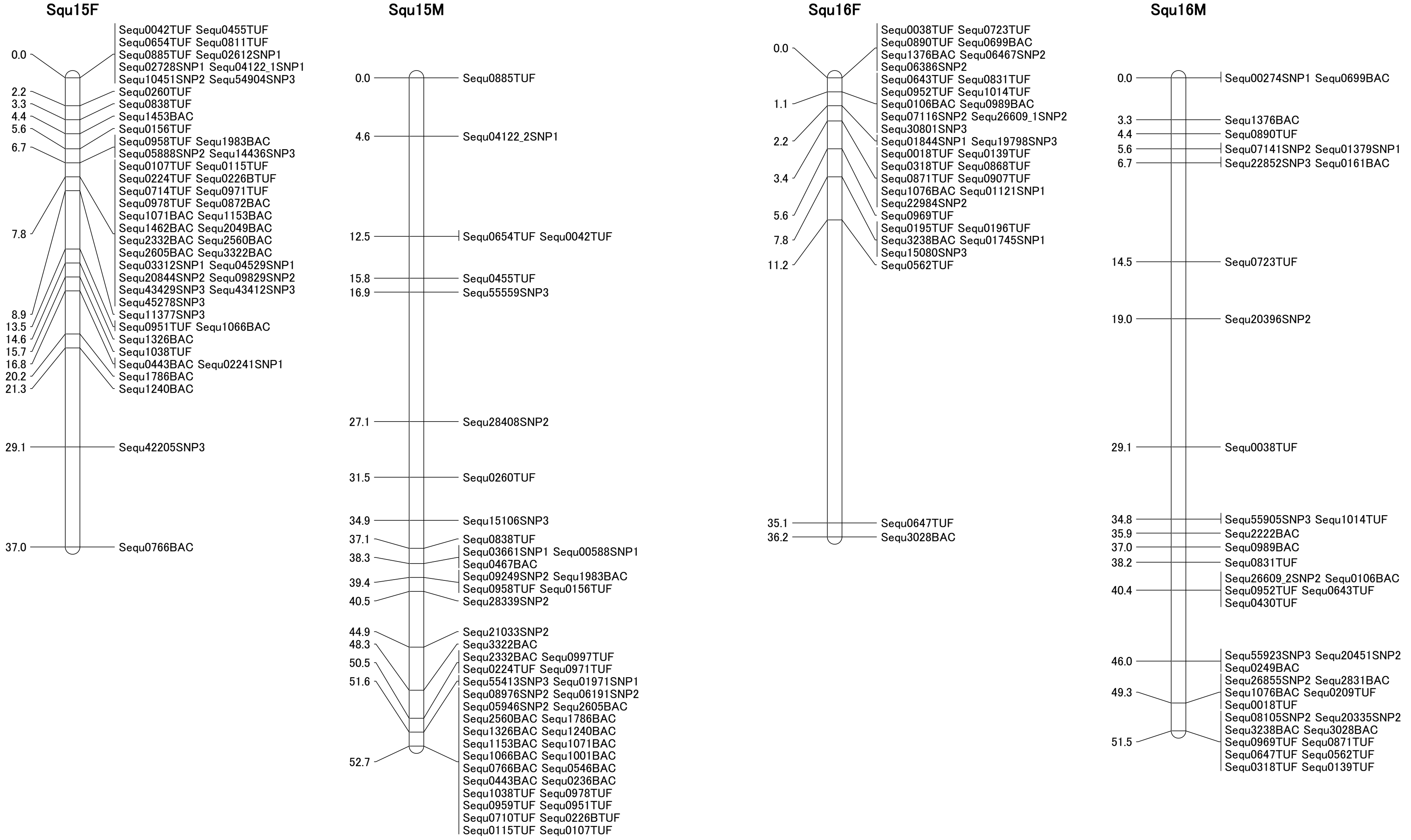
Squ15F
0.0
Sequ0042TUF Sequ0455TUF
Sequ0654TUF Sequ0811TUF
Sequ0885TUF Sequ02612SNP1
Sequ02728SNP1 Sequ04122_1SNP1
Sequ10451SNP2 Sequ54904SNP3
2.2
Sequ0260TUF
3.3
Sequ0838TUF
4.4
Sequ1453BAC
5.6
Sequ0156TUF
6.7
Sequ0958TUF Sequ1983BAC
Sequ05888SNP2 Sequ14436SNP3
Sequ0107TUF Sequ0115TUF
Sequ0224TUF Sequ0226BTUF
Sequ0714TUF Sequ0971TUF
Sequ0978TUF Sequ0872BAC
Sequ1071BAC Sequ1153BAC
7.8
Sequ1462BAC Sequ2049BAC
Sequ2332BAC Sequ2560BAC
Sequ2605BAC Sequ3322BAC
Sequ03312SNP1 Sequ04529SNP1
Sequ20844SNP2 Sequ09829SNP2
Sequ43429SNP3 Sequ43412SNP3
Sequ45278SNP3
8.9
Sequ11377SNP3
13.5
Sequ0951TUF Sequ1066BAC
14.6
Sequ1326BAC
15.7
Sequ1038TUF
16.8
Sequ0443BAC Sequ02241SNP1
20.2
Sequ1786BAC
21.3
Sequ1240BAC
29.1
Sequ42205SNP3
37.0
Sequ0766BAC
Squ15M
0.0
Sequ0885TUF
4.6
Sequ04122_2SNP1
12.5
Sequ0654TUF Sequ0042TUF
15.8
Sequ0455TUF
16.9
Sequ55559SNP3
27.1
Sequ28408SNP2
31.5
Sequ0260TUF
34.9
Sequ15106SNP3
37.1
Sequ0838TUF
38.3
Sequ03661SNP1 Sequ00588SNP1
Sequ0467BAC
39.4
Sequ09249SNP2 Sequ1983BAC
Sequ0958TUF Sequ0156TUF
40.5
Sequ28339SNP2
44.9
Sequ21033SNP2
48.3
Sequ3322BAC
50.5
Sequ2332BAC Sequ0997TUF
Sequ0224TUF Sequ0971TUF
51.6
Sequ55413SNP3 Sequ01971SNP1
Sequ08976SNP2 Sequ06191SNP2
Sequ05946SNP2 Sequ2605BAC
Sequ2560BAC Sequ1786BAC
Sequ1326BAC Sequ1240BAC
Sequ1153BAC Sequ1071BAC
52.7
Sequ1066BAC Sequ1001BAC
Sequ0766BAC Sequ0546BAC
Sequ0443BAC Sequ0236BAC
Sequ1038TUF Sequ0978TUF
Sequ0959TUF Sequ0951TUF
Sequ0710TUF Sequ0226BTUF
Sequ0115TUF Sequ0107TUF
Squ16F
0.0
Sequ0038TUF Sequ0723TUF
Sequ0890TUF Sequ0699BAC
Sequ1376BAC Sequ06467SNP2
Sequ06386SNP2
Sequ0643TUF Sequ0831TUF
1.1
Sequ0952TUF Sequ1014TUF
Sequ0106BAC Sequ0989BAC
Sequ07116SNP2 Sequ26609_1SNP2
Sequ30801SNP3
2.2
Sequ01844SNP1 Sequ19798SNP3
Sequ0018TUF Sequ0139TUF
Sequ0318TUF Sequ0868TUF
3.4
Sequ0871TUF Sequ0907TUF
Sequ1076BAC Sequ01121SNP1
Sequ22984SNP2
5.6
Sequ0969TUF
7.8
Sequ0195TUF Sequ0196TUF
Sequ3238BAC Sequ01745SNP1
Sequ15080SNP3
11.2
Sequ0562TUF
35.1
Sequ0647TUF
36.2
Sequ3028BAC
Squ16M
0.0
Sequ00274SNP1 Sequ0699BAC
3.3
Sequ1376BAC
4.4
Sequ0890TUF
5.6
Sequ07141SNP2 Sequ01379SNP1
6.7
Sequ22852SNP3 Sequ0161BAC
14.5
Sequ0723TUF
19.0
Sequ20396SNP2
29.1
Sequ0038TUF
34.8
Sequ55905SNP3 Sequ1014TUF
35.9
Sequ2222BAC
37.0
Sequ0989BAC
38.2
Sequ0831TUF
40.4
Sequ26609_2SNP2 Sequ0106BAC
Sequ0952TUF Sequ0643TUF
Sequ0430TUF
46.0
Sequ55923SNP3 Sequ20451SNP2
Sequ0249BAC
49.3
Sequ26855SNP2 Sequ2831BAC
Sequ1076BAC Sequ0209TUF
Sequ0018TUF
51.5
Sequ08105SNP2 Sequ20335SNP2
Sequ3238BAC Sequ3028BAC
Sequ0969TUF Sequ0871TUF
Sequ0647TUF Sequ0562TUF
Sequ0318TUF Sequ0139TUF

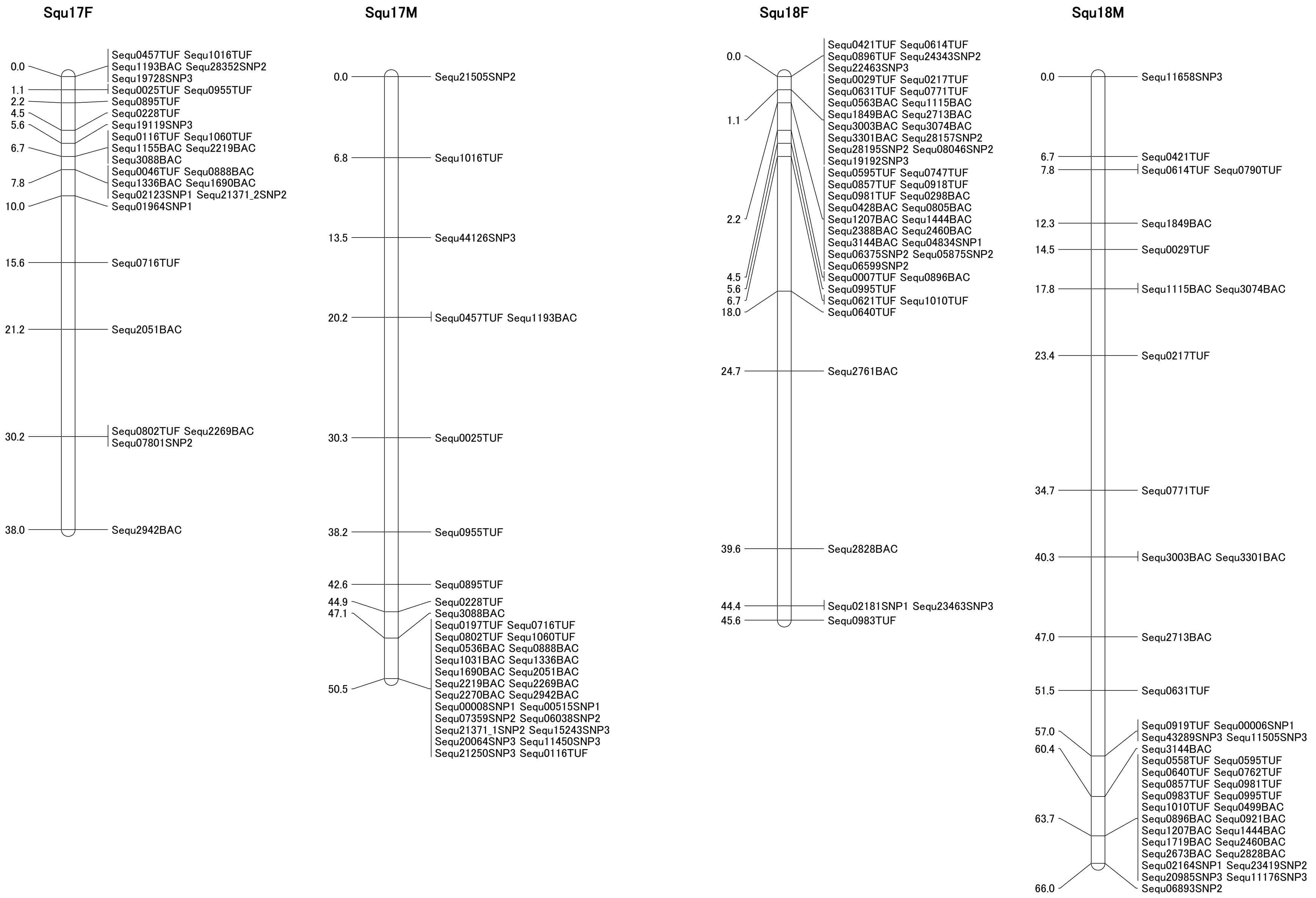

Squ17F
0.0 Sequ0457TUF Sequ1016TUF
Sequ1193BAC Sequ28352SNP2
Sequ19728SNP3
1.1 Sequ0025TUF Sequ0955TUF
2.2 Sequ0895TUF
4.5 Sequ0228TUF
5.6 Sequ19119SNP3
6.7 Sequ0116TUF Sequ1060TUF
Sequ1155BAC Sequ2219BAC
Sequ3088BAC
7.8 Sequ0046TUF Sequ0888BAC
Sequ1336BAC Sequ1690BAC
Sequ02123SNP1 Sequ21371_2SNP2
10.0 Sequ01964SNP1
15.6 Sequ0716TUF
21.2 Sequ2051BAC
30.2 Sequ0802TUF Sequ2269BAC
Sequ07801SNP2
38.0 Sequ2942BAC
Squ17M
0.0 Sequ21505SNP2
6.8 Sequ1016TUF
13.5 Sequ44126SNP3
20.2 Sequ0457TUF Sequ1193BAC
30.3 Sequ0025TUF
38.2 Sequ0955TUF
42.6 Sequ0895TUF
44.9 Sequ0228TUF
47.1 Sequ3088BAC
Sequ0197TUF Sequ0716TUF
Sequ0802TUF Sequ1060TUF
Sequ0536BAC Sequ0888BAC
Sequ1031BAC Sequ1336BAC
Sequ1690BAC Sequ2051BAC
Sequ2219BAC Sequ2269BAC
50.5 Sequ2270BAC Sequ2942BAC
Sequ00008SNP1 Sequ00515SNP1
Sequ07359SNP2 Sequ06038SNP2
Sequ21371_1SNP2 Sequ15243SNP3
Sequ20064SNP3 Sequ11450SNP3
Sequ21250SNP3 Sequ0116TUF
Squ18F
0.0 Sequ0421TUF Sequ0614TUF
Sequ0896TUF Sequ24343SNP2
Sequ22463SNP3
1.1 Sequ0029TUF Sequ0217TUF
Sequ0631TUF Sequ0771TUF
Sequ0563BAC Sequ1115BAC
Sequ1849BAC Sequ2713BAC
Sequ3003BAC Sequ3074BAC
Sequ3301BAC Sequ28157SNP2
Sequ28195SNP2 Sequ08046SNP2
Sequ19192SNP3
2.2 Sequ0595TUF Sequ0747TUF
Sequ0857TUF Sequ0918TUF
Sequ0981TUF Sequ0298BAC
Sequ0428BAC Sequ0805BAC
Sequ1207BAC Sequ1444BAC
Sequ2388BAC Sequ2460BAC
Sequ3144BAC Sequ04834SNP1
Sequ06375SNP2 Sequ05875SNP2
Sequ06599SNP2
4.5 Sequ0007TUF Sequ0896BAC
5.6 Sequ0995TUF
6.7 Sequ0621TUF Sequ1010TUF
18.0 Sequ0640TUF
24.7 Sequ2761BAC
39.6 Sequ2828BAC
44.4 Sequ02181SNP1 Sequ23463SNP3
45.6 Sequ0983TUF
Squ18M
0.0 Sequ11658SNP3
6.7 Sequ0421TUF
7.8 Sequ0614TUF Sequ0790TUF
12.3 Sequ1849BAC
14.5 Sequ0029TUF
17.8 Sequ1115BAC Sequ3074BAC
23.4 Sequ0217TUF
34.7 Sequ0771TUF
40.3 Sequ3003BAC Sequ3301BAC
47.0 Sequ2713BAC
51.5 Sequ0631TUF
57.0 Sequ0919TUF Sequ00006SNP1
Sequ43289SNP3 Sequ11505SNP3
60.4 Sequ3144BAC
Sequ0558TUF Sequ0595TUF
Sequ0640TUF Sequ0762TUF
Sequ0857TUF Sequ0981TUF
Sequ0983TUF Sequ0995TUF
Sequ1010TUF Sequ0499BAC
63.7 Sequ0896BAC Sequ0921BAC
Sequ1207BAC Sequ1444BAC
Sequ1719BAC Sequ2460BAC
Sequ2673BAC Sequ2828BAC
Sequ02164SNP1 Sequ23419SNP2
Sequ20985SNP3 Sequ11176SNP3
66.0 Sequ06893SNP2

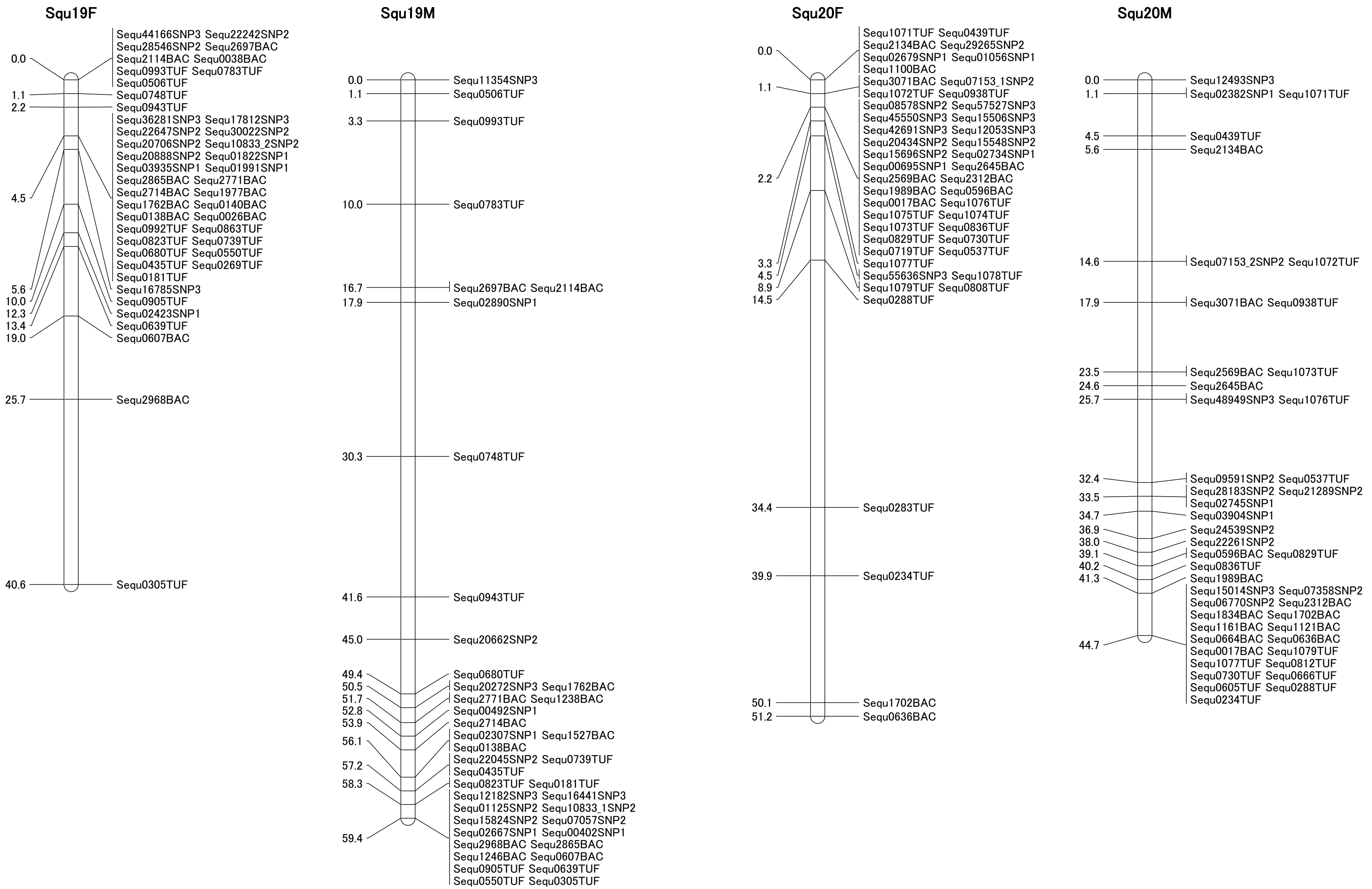
Squ19F
0.0
Sequ44166SNP3 Sequ22242SNP2
Sequ28546SNP2 Sequ2697BAC
Sequ2114BAC Sequ0038BAC
Sequ0993TUF Sequ0783TUF
Sequ0506TUF
1.1
Sequ0748TUF
2.2
Sequ0943TUF
4.5
Sequ36281SNP3 Sequ17812SNP3
Sequ22647SNP2 Sequ30022SNP2
Sequ20706SNP2 Sequ10833_2SNP2
Sequ20888SNP2 Sequ01822SNP1
Sequ03935SNP1 Sequ01991SNP1
Sequ2865BAC Sequ2771BAC
Sequ2714BAC Sequ1977BAC
Sequ1762BAC Sequ0140BAC
Sequ0138BAC Sequ0026BAC
Sequ0992TUF Sequ0863TUF
Sequ0823TUF Sequ0739TUF
Sequ0680TUF Sequ0550TUF
Sequ0435TUF Sequ0269TUF
Sequ0181TUF
5.6
Sequ16785SNP3
10.0
Sequ0905TUF
12.3
Sequ02423SNP1
13.4
Sequ0639TUF
19.0
Sequ0607BAC
25.7
Sequ2968BAC
40.6
Sequ0305TUF
Squ19M
0.0
Sequ11354SNP3
1.1
Sequ0506TUF
3.3
Sequ0993TUF
10.0
Sequ0783TUF
16.7
Sequ2697BAC Sequ2114BAC
17.9
Sequ02890SNP1
30.3
Sequ0748TUF
41.6
Sequ0943TUF
45.0
Sequ20662SNP2
49.4
Sequ0680TUF
50.5
Sequ20272SNP3 Sequ1762BAC
51.7
Sequ2771BAC Sequ1238BAC
52.8
Sequ00492SNP1
53.9
Sequ2714BAC
56.1
Sequ02307SNP1 Sequ1527BAC
Sequ0138BAC
57.2
Sequ22045SNP2 Sequ0739TUF
Sequ0435TUF
58.3
Sequ0823TUF Sequ0181TUF
59.4
Sequ12182SNP3 Sequ16441SNP3
Sequ01125SNP2 Sequ10833_1SNP2
Sequ15824SNP2 Sequ07057SNP2
Sequ02667SNP1 Sequ00402SNP1
Sequ2968BAC Sequ2865BAC
Sequ1246BAC Sequ0607BAC
Sequ0905TUF Sequ0639TUF
Sequ0550TUF Sequ0305TUF
Squ20F
0.0
Sequ1071TUF Sequ0439TUF
Sequ2134BAC Sequ29265SNP2
Sequ02679SNP1 Sequ01056SNP1
Sequ1100BAC
1.1
Sequ3071BAC Sequ07153_1SNP2
Sequ1072TUF Sequ0938TUF
2.2
Sequ08578SNP2 Sequ57527SNP3
Sequ45550SNP3 Sequ15506SNP3
Sequ42691SNP3 Sequ12053SNP3
Sequ20434SNP2 Sequ15548SNP2
Sequ15696SNP2 Sequ02734SNP1
Sequ00695SNP1 Sequ2645BAC
Sequ2569BAC Sequ2312BAC
Sequ1989BAC Sequ0596BAC
Sequ0017BAC Sequ1076TUF
Sequ1075TUF Sequ1074TUF
Sequ1073TUF Sequ0836TUF
Sequ0829TUF Sequ0730TUF
Sequ0719TUF Sequ0537TUF
3.3
Sequ1077TUF
4.5
Sequ55636SNP3 Sequ1078TUF
8.9
Sequ1079TUF Sequ0808TUF
14.5
Sequ0288TUF
34.4
Sequ0283TUF
39.9
Sequ0234TUF
50.1
Sequ1702BAC
51.2
Sequ0636BAC
Squ20M
0.0
Sequ12493SNP3
1.1
Sequ02382SNP1 Sequ1071TUF
4.5
Sequ0439TUF
5.6
Sequ2134BAC
14.6
Sequ07153_2SNP2 Sequ1072TUF
17.9
Sequ3071BAC Sequ0938TUF
23.5
Sequ2569BAC Sequ1073TUF
24.6
Sequ2645BAC
25.7
Sequ48949SNP3 Sequ1076TUF
32.4
Sequ09591SNP2 Sequ0537TUF
33.5
Sequ28183SNP2 Sequ21289SNP2
Sequ02745SNP1
34.7
Sequ03904SNP1
36.9
Sequ24539SNP2
38.0
Sequ22261SNP2
39.1
Sequ0596BAC Sequ0829TUF
40.2
Sequ0836TUF
41.3
Sequ1989BAC
44.7
Sequ15014SNP3 Sequ07358SNP2
Sequ06770SNP2 Sequ2312BAC
Sequ1834BAC Sequ1702BAC
Sequ1161BAC Sequ1121BAC
Sequ0664BAC Sequ0636BAC
Sequ0017BAC Sequ1079TUF
Sequ1077TUF Sequ0812TUF
Sequ0730TUF Sequ0666TUF
Sequ0605TUF Sequ0288TUF
Sequ0234TUF

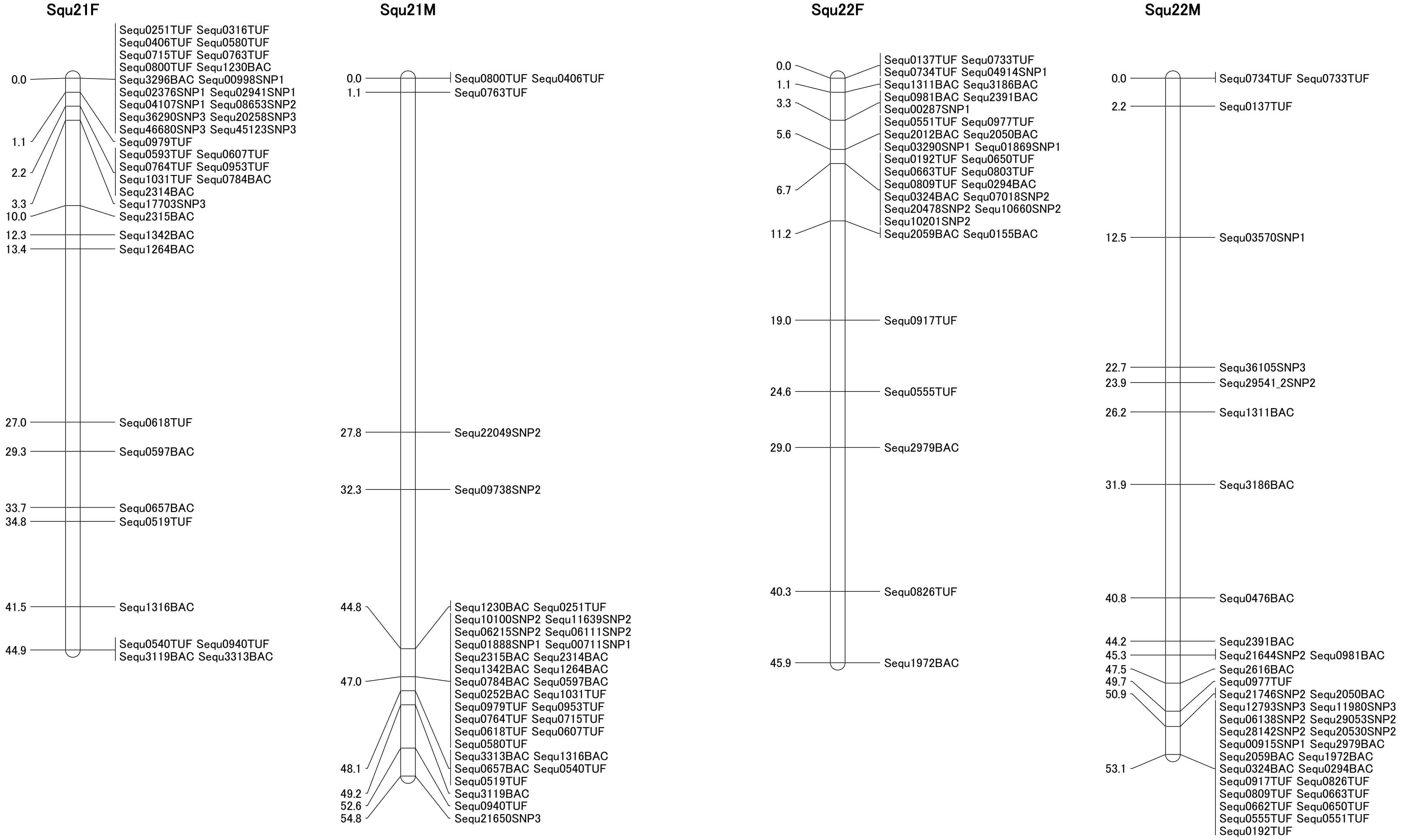
Squ21F
0.0 Sequ0251TUF Sequ0316TUF
Sequ0406TUF Sequ0580TUF
Sequ0715TUF Sequ0763TUF
Sequ0800TUF Sequ1230BAC
Sequ3296BAC Sequ00998SNP1
Sequ02376SNP1 Sequ02941SNP1
Sequ04107SNP1 Sequ08653SNP2
Sequ36290SNP3 Sequ20258SNP3
Sequ46680SNP3 Sequ45123SNP3
1.1 Sequ0979TUF
2.2 Sequ0593TUF Sequ0607TUF
Sequ0764TUF Sequ0953TUF
Sequ1031TUF Sequ0784BAC
Sequ2314BAC
3.3 Sequ17703SNP3
10.0 Sequ2315BAC
12.3 Sequ1342BAC
13.4 Sequ1264BAC
27.0 Sequ0618TUF
29.3 Sequ0597BAC
33.7 Sequ0657BAC
34.8 Sequ0519TUF
41.5 Sequ1316BAC
44.9 Sequ0540TUF Sequ0940TUF
Sequ3119BAC Sequ3313BAC
Squ21M
0.0 Sequ0800TUF Sequ0406TUF
1.1 Sequ0763TUF
27.8 Sequ22049SNP2
32.3 Sequ09738SNP2
44.8 Sequ1230BAC Sequ0251TUF
Sequ10100SNP2 Sequ11639SNP2
Sequ06215SNP2 Sequ06111SNP2
Sequ01888SNP1 Sequ00711SNP1
Sequ2315BAC Sequ2314BAC
Sequ1342BAC Sequ1264BAC
47.0 Sequ0784BAC Sequ0597BAC
Sequ0252BAC Sequ1031TUF
Sequ0979TUF Sequ0953TUF
Sequ0764TUF Sequ0715TUF
Sequ0618TUF Sequ0607TUF
Sequ0580TUF
48.1 Sequ3313BAC Sequ1316BAC
Sequ0657BAC Sequ0540TUF
Sequ0519TUF
49.2 Sequ3119BAC
52.6 Sequ0940TUF
54.8 Sequ21650SNP3
Squ22F
0.0 Sequ0137TUF Sequ0733TUF
Sequ0734TUF Sequ04914SNP1
1.1 Sequ1311BAC Sequ3186BAC
3.3 Sequ0981BAC Sequ2391BAC
Sequ00287SNP1
5.6 Sequ0551TUF Sequ0977TUF
Sequ2012BAC Sequ2050BAC
Sequ03290SNP1 Sequ01869SNP1
6.7 Sequ0192TUF Sequ0650TUF
Sequ0663TUF Sequ0803TUF
Sequ0809TUF Sequ0294BAC
Sequ0324BAC Sequ07018SNP2
Sequ20478SNP2 Sequ10660SNP2
Sequ10201SNP2
11.2 Sequ2059BAC Sequ0155BAC
19.0 Sequ0917TUF
24.6 Sequ0555TUF
29.0 Sequ2979BAC
40.3 Sequ0826TUF
45.9 Sequ1972BAC
Squ22M
0.0 Sequ0734TUF Sequ0733TUF
2.2 Sequ0137TUF
12.5 Sequ03570SNP1
22.7 Sequ36105SNP3
23.9 Sequ29541_2SNP2
26.2 Sequ1311BAC
31.9 Sequ3186BAC
40.8 Sequ0476BAC
44.2 Sequ2391BAC
45.3 Sequ21644SNP2 Sequ0981BAC
47.5 Sequ2616BAC
49.7 Sequ0977TUF
50.9 Sequ21746SNP2 Sequ2050BAC
Sequ12793SNP3 Sequ11980SNP3
Sequ06138SNP2 Sequ29053SNP2
Sequ28142SNP2 Sequ20530SNP2
Sequ00915SNP1 Sequ2979BAC
53.1 Sequ2059BAC Sequ1972BAC
Sequ0324BAC Sequ0294BAC
Sequ0917TUF Sequ0826TUF
Sequ0809TUF Sequ0663TUF
Sequ0662TUF Sequ0650TUF
Sequ0555TUF Sequ0551TUF
Sequ0192TUF

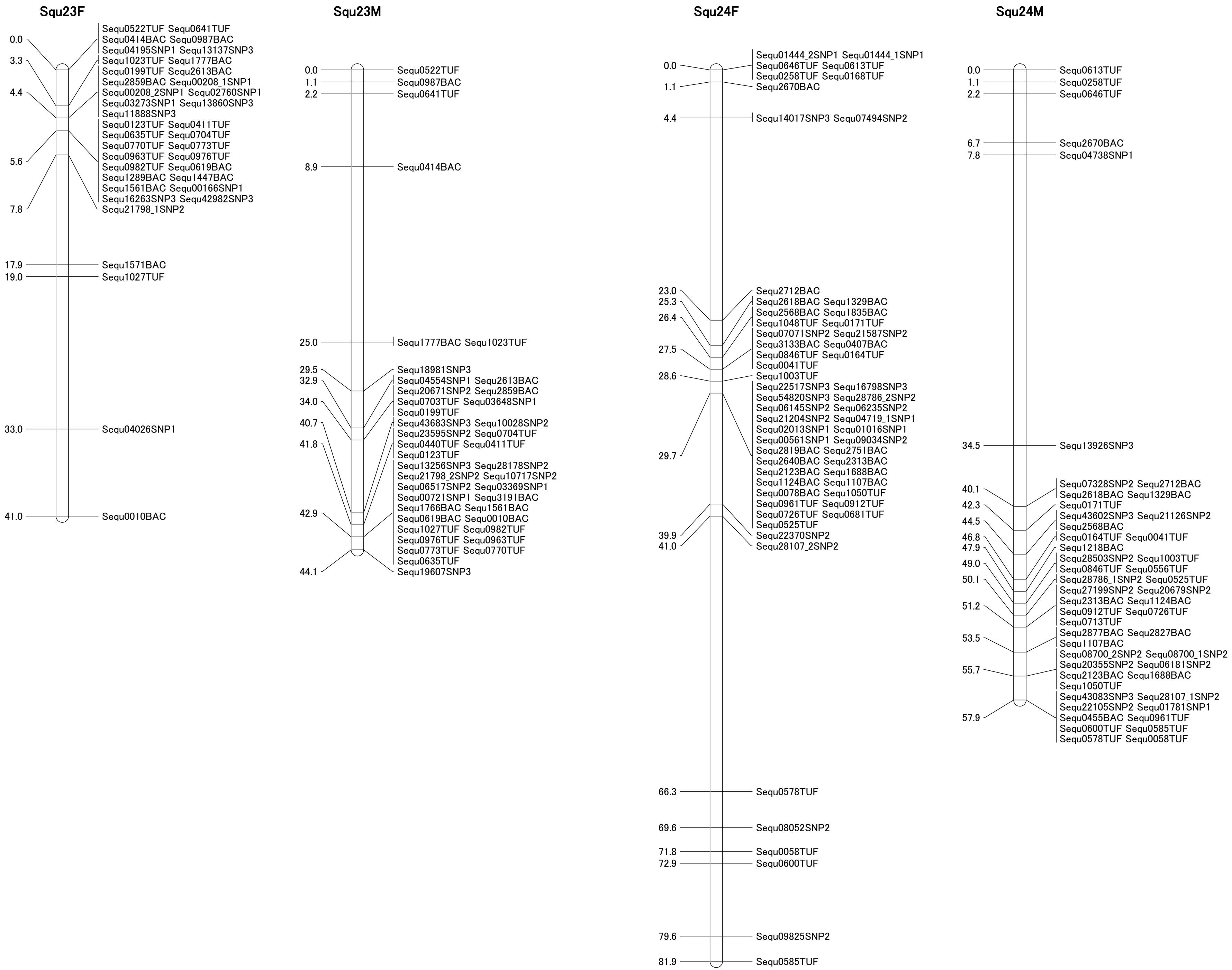
Squ23F
0.0
3.3
4.4
5.6
7.8
Sequ0522TUF Sequ0641TUF
Sequ0414BAC Sequ0987BAC
Sequ04195SNP1 Sequ13137SNP3
Sequ1023TUF Sequ1777BAC
Sequ0199TUF Sequ2613BAC
Sequ2859BAC Sequ00208_1SNP1
Sequ00208_2SNP1 Sequ02760SNP1
Sequ03273SNP1 Sequ13860SNP3
Sequ11888SNP3
Sequ0123TUF Sequ0411TUF
Sequ0635TUF Sequ0704TUF
Sequ0770TUF Sequ0773TUF
Sequ0963TUF Sequ0976TUF
Sequ0982TUF Sequ0619BAC
Sequ1289BAC Sequ1447BAC
Sequ1561BAC Sequ00166SNP1
Sequ16263SNP3 Sequ42982SNP3
Sequ21798_1SNP2
17.9 Sequ1571BAC
19.0 Sequ1027TUF
33.0 Sequ04026SNP1
41.0 Sequ0010BAC
Squ23M
0.0 Sequ0522TUF
1.1 Sequ0987BAC
2.2 Sequ0641TUF
8.9 Sequ0414BAC
25.0 Sequ1777BAC Sequ1023TUF
29.5
32.9
34.0
40.7
41.8
42.9
44.1
Sequ18981SNP3
Sequ04554SNP1 Sequ2613BAC
Sequ20671SNP2 Sequ2859BAC
Sequ0703TUF Sequ03648SNP1
Sequ0199TUF
Sequ43683SNP3 Sequ10028SNP2
Sequ23595SNP2 Sequ0704TUF
Sequ0440TUF Sequ0411TUF
Sequ0123TUF
Sequ13256SNP3 Sequ28178SNP2
Sequ21798_2SNP2 Sequ10717SNP2
Sequ06517SNP2 Sequ03369SNP1
Sequ00721SNP1 Sequ3191BAC
Sequ1766BAC Sequ1561BAC
Sequ0619BAC Sequ0010BAC
Sequ1027TUF Sequ0982TUF
Sequ0976TUF Sequ0963TUF
Sequ0773TUF Sequ0770TUF
Sequ0635TUF
Sequ19607SNP3
Squ24F
0.0
1.1
Sequ01444_2SNP1 Sequ01444_1SNP1
Sequ0646TUF Sequ0613TUF
Sequ0258TUF Sequ0168TUF
Sequ2670BAC
4.4 Sequ14017SNP3 Sequ07494SNP2
23.0
25.3
26.4
27.5
28.6
29.7
39.9
41.0
Sequ2712BAC
Sequ2618BAC Sequ1329BAC
Sequ2568BAC Sequ1835BAC
Sequ1048TUF Sequ0171TUF
Sequ07071SNP2 Sequ21587SNP2
Sequ3133BAC Sequ0407BAC
Sequ0846TUF Sequ0164TUF
Sequ0041TUF
Sequ1003TUF
Sequ22517SNP3 Sequ16798SNP3
Sequ54820SNP3 Sequ28786_2SNP2
Sequ06145SNP2 Sequ06235SNP2
Sequ21204SNP2 Sequ04719_1SNP1
Sequ02013SNP1 Sequ01016SNP1
Sequ00561SNP1 Sequ09034SNP2
Sequ2819BAC Sequ2751BAC
Sequ2640BAC Sequ2313BAC
Sequ2123BAC Sequ1688BAC
Sequ1124BAC Sequ1107BAC
Sequ0078BAC Sequ1050TUF
Sequ0961TUF Sequ0912TUF
Sequ0726TUF Sequ0681TUF
Sequ0525TUF
Sequ22370SNP2
Sequ28107_2SNP2
66.3 Sequ0578TUF
69.6 Sequ08052SNP2
71.8 Sequ0058TUF
72.9 Sequ0600TUF
79.6 Sequ09825SNP2
81.9 Sequ0585TUF
Squ24M
0.0 Sequ0613TUF
1.1 Sequ0258TUF
2.2 Sequ0646TUF
6.7 Sequ2670BAC
7.8 Sequ04738SNP1
34.5 Sequ13926SNP3
40.1
42.3
44.5
46.8
47.9
49.0
50.1
51.2
53.5
55.7
57.9
Sequ07328SNP2 Sequ2712BAC
Sequ2618BAC Sequ1329BAC
Sequ0171TUF
Sequ43602SNP3 Sequ21126SNP2
Sequ2568BAC
Sequ0164TUF Sequ0041TUF
Sequ1218BAC
Sequ28503SNP2 Sequ1003TUF
Sequ0846TUF Sequ0556TUF
Sequ28786_1SNP2 Sequ0525TUF
Sequ27199SNP2 Sequ20679SNP2
Sequ2313BAC Sequ1124BAC
Sequ0912TUF Sequ0726TUF
Sequ0713TUF
Sequ2877BAC Sequ2827BAC
Sequ1107BAC
Sequ08700_2SNP2 Sequ08700_1SNP2
Sequ20355SNP2 Sequ06181SNP2
Sequ2123BAC Sequ1688BAC
Sequ1050TUF
Sequ43083SNP3 Sequ28107_1SNP2
Sequ22105SNP2 Sequ01781SNP1
Sequ0455BAC Sequ0961TUF
Sequ0600TUF Sequ0585TUF
Sequ0578TUF Sequ0058TUF
