# Supplementary figures and images for: Second generation physical and linkage maps of yellowtail (Seriola quinqueradiata) and comparison of synteny with four model fish
Source: BMC Genomics. 2015 May 24;16(1):406. doi: 10.1186/s12864-015-1600-7 (PMC4493941; doi:10.1186/s12864-015-1600-7)

## SQ1

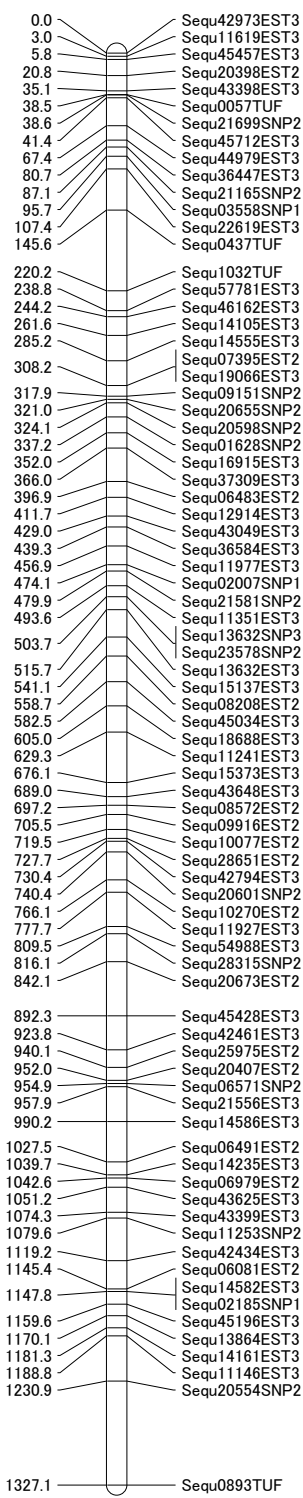

## SQ2

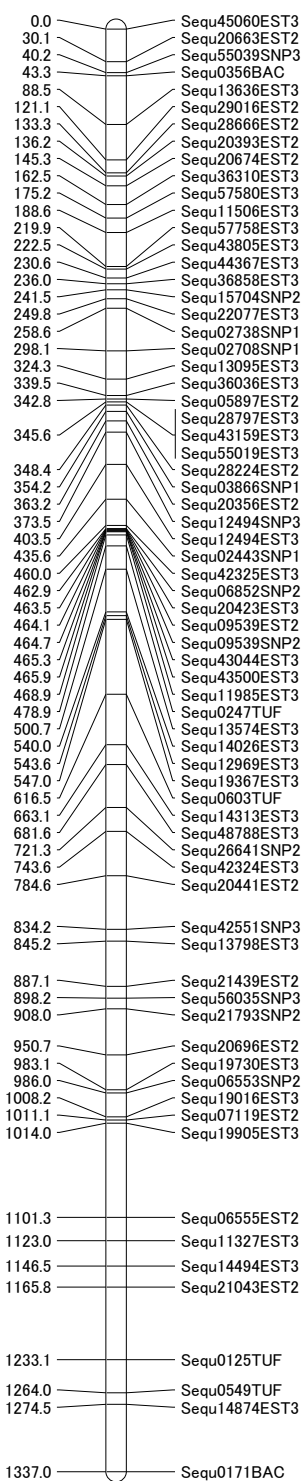

## SQ3

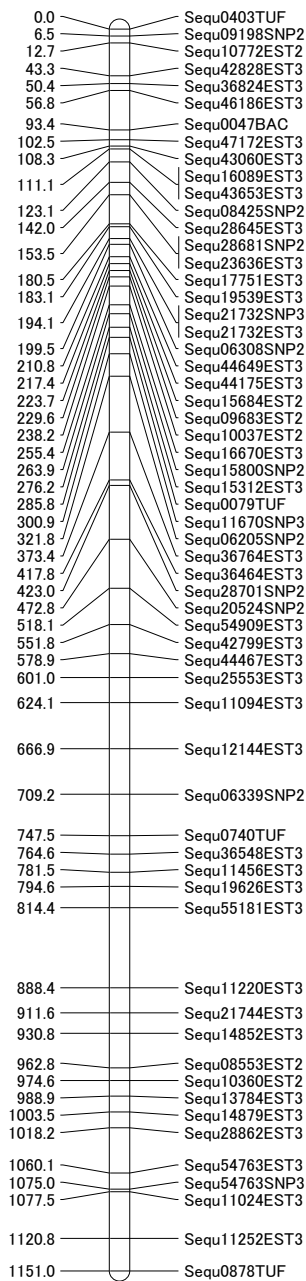

## SQ4

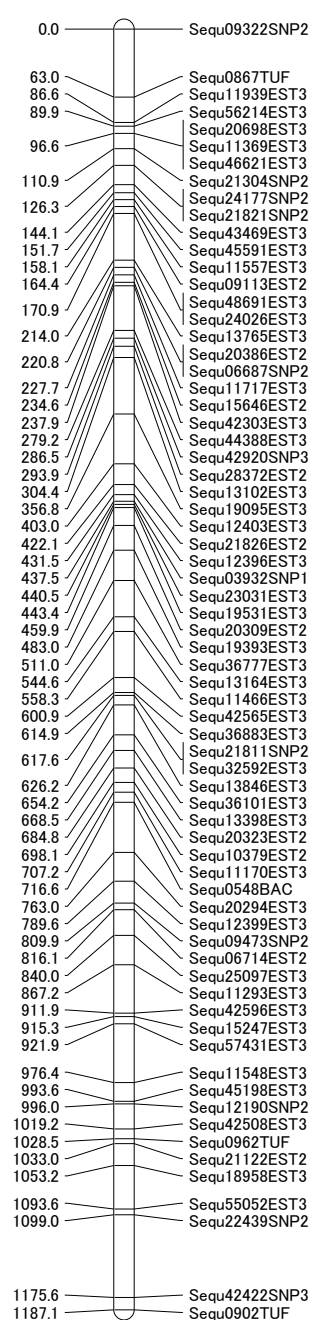

SQ5

SQ6

SQ7

SQ8

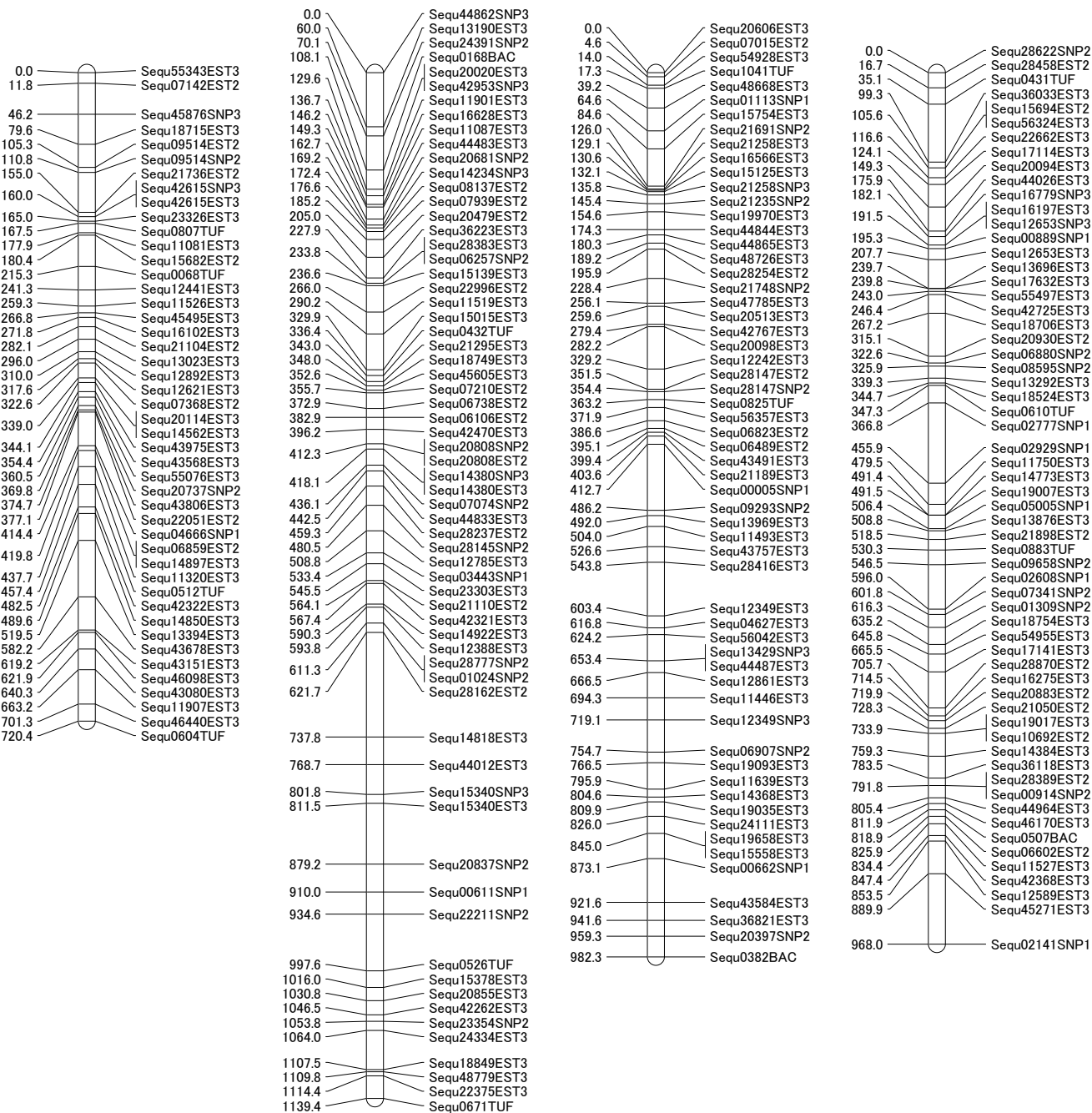

## SQ9

## SQ10

## SQ11

## SQ12

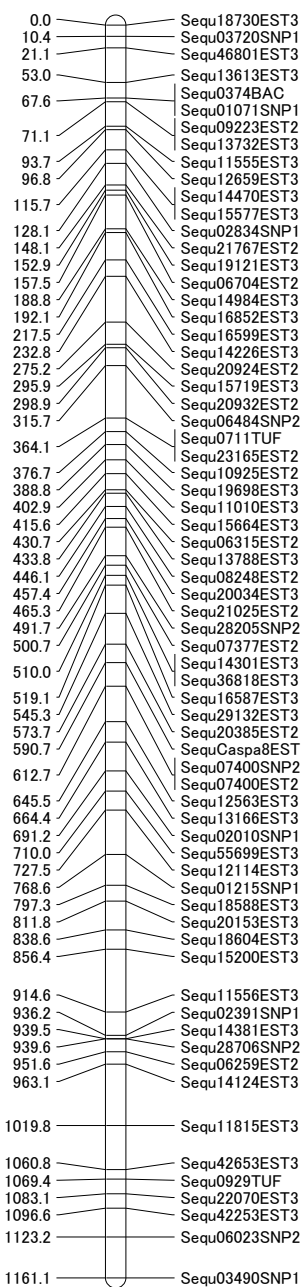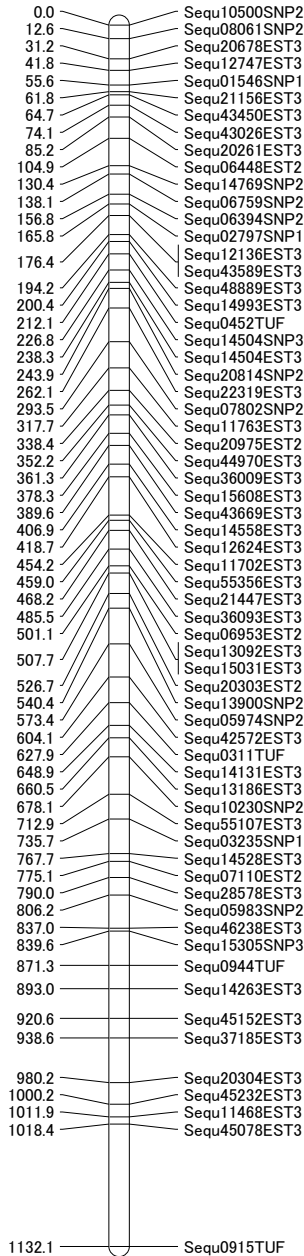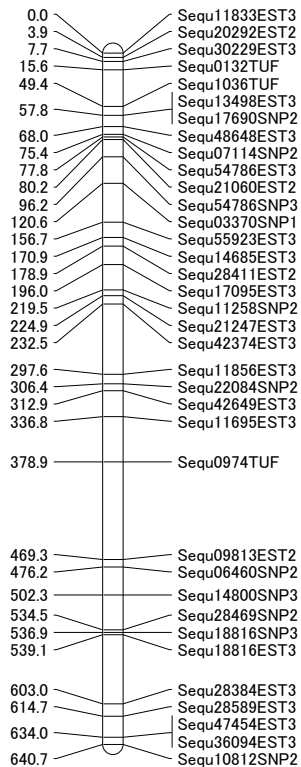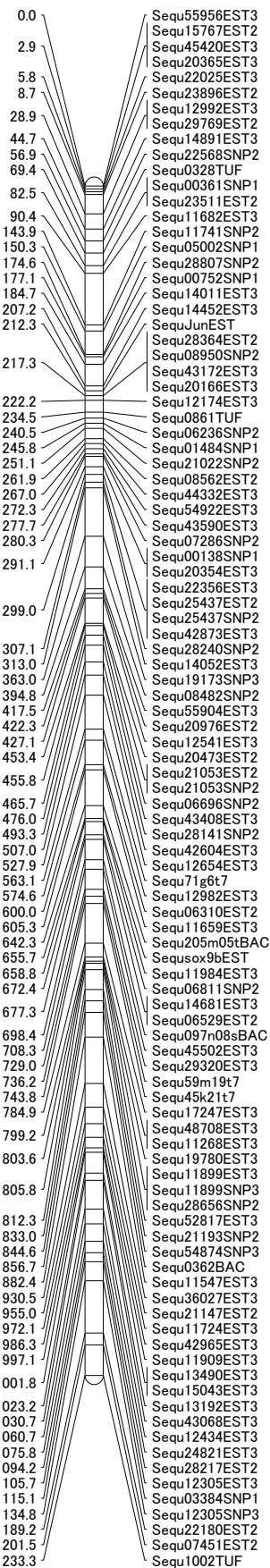

SQ13

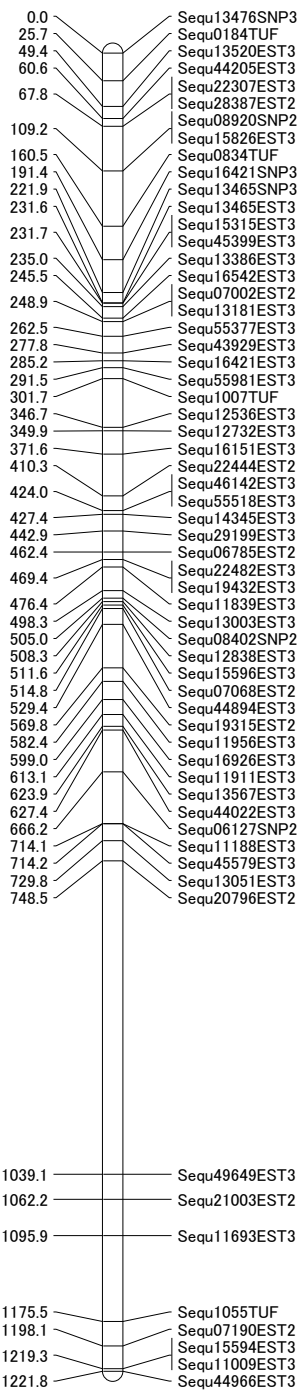

SQ14

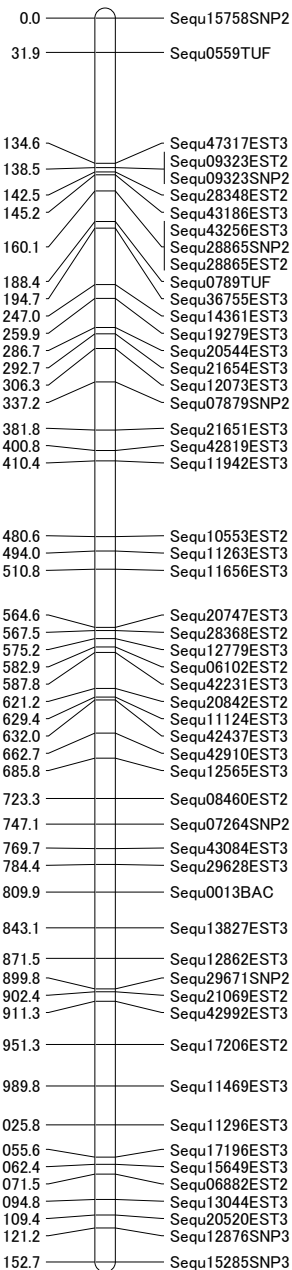

SQ15

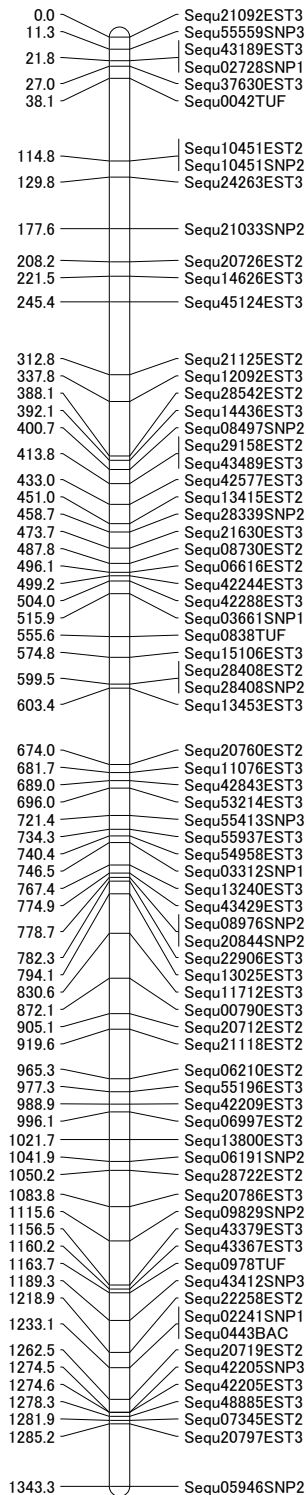

SQ16

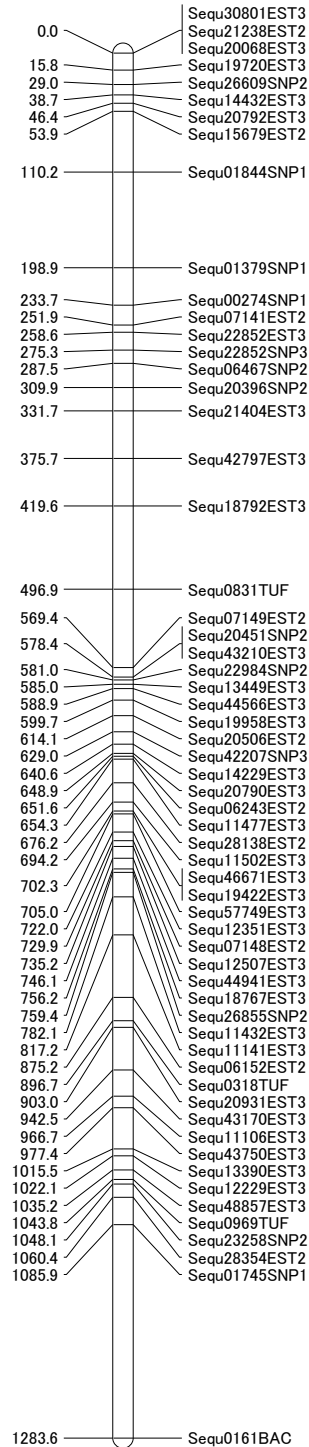

SQ17

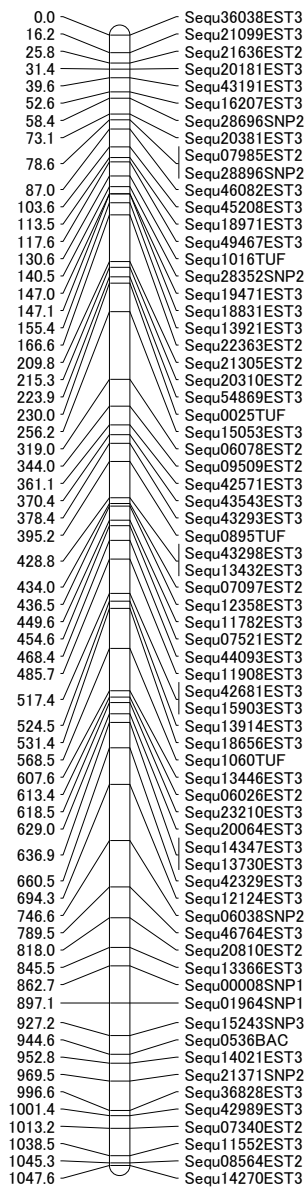

SQ18

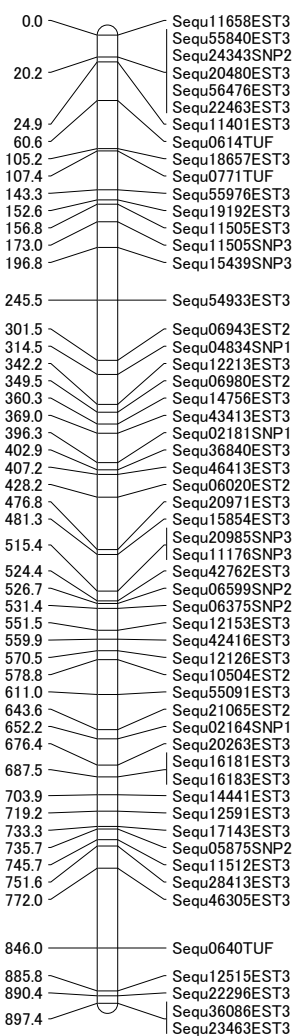

SQ19

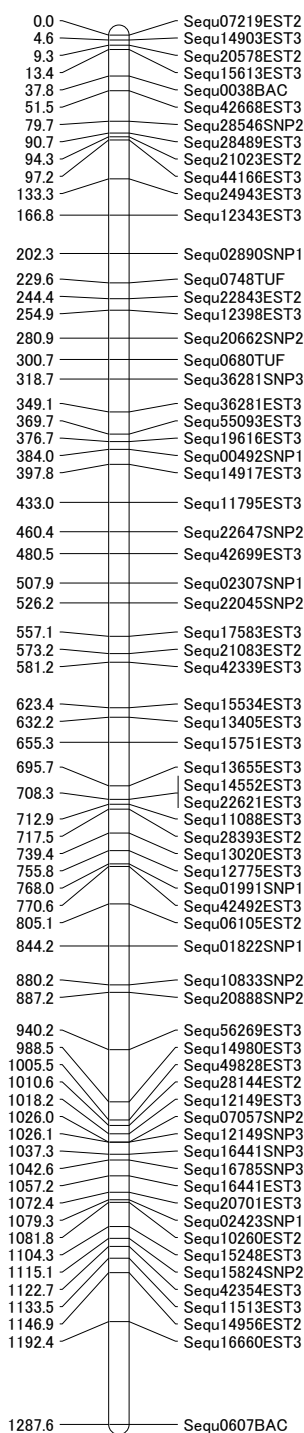

SQ20

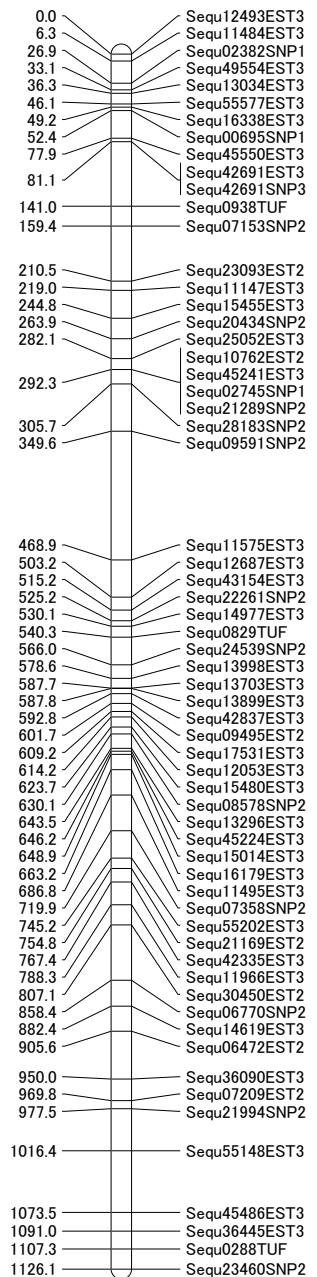

SQ21

SQ22

SQ23

SQ24

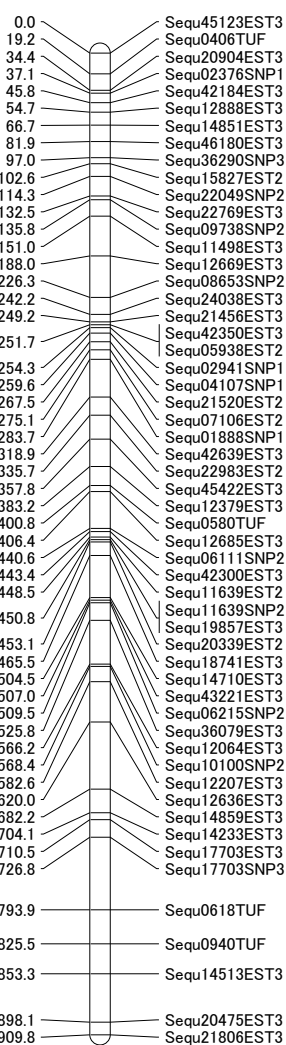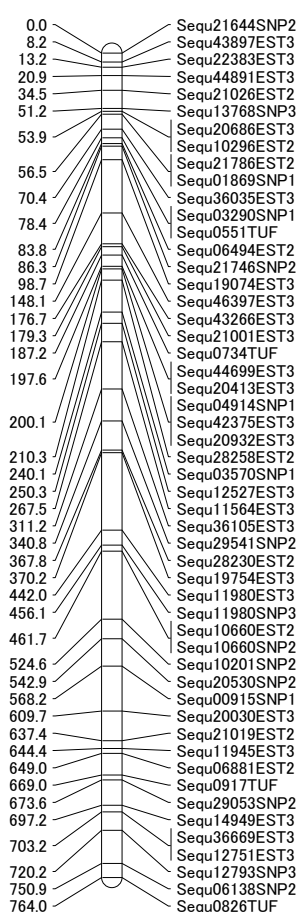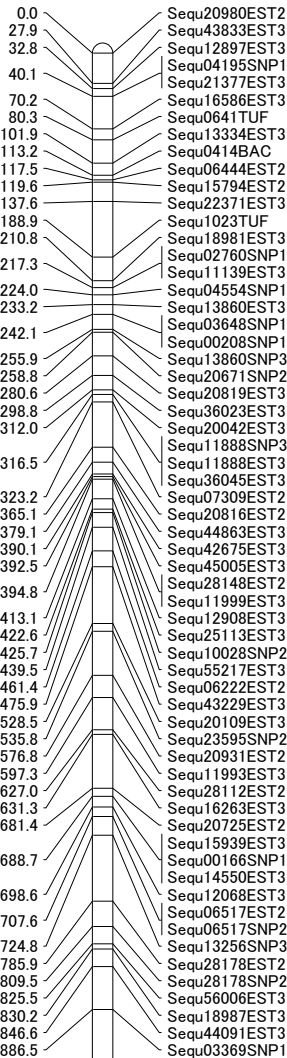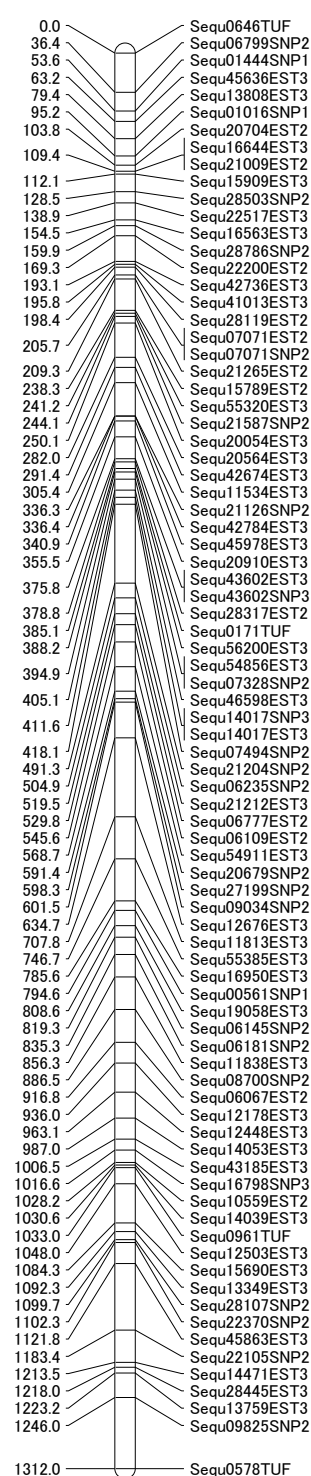

Supplement: Additional file 2: — RH map of yellowtail. Distances between markers are shown in centiRays (cR). [file 12864_2015_1600_MOESM2_ESM.pdf]
