# Supplementary material for: Second generation physical and linkage maps of yellowtail (Seriola quinqueradiata) and comparison of synteny with four model fish
Source: BMC Genomics. 2015 May 24;16(1):406. doi: 10.1186/s12864-015-1600-7 (PMC4493941; doi:10.1186/s12864-015-1600-7)
Supplement: Additional file 3: — Comparison of the radiation hybrid (RH) map and the linkage map. Distances between markers are shown in centiRays (cR) on the RH map and in 10−1 centiMorgans (cM) on the linkage map. [file 12864_2015_1600_MOESM3_ESM.pdf]

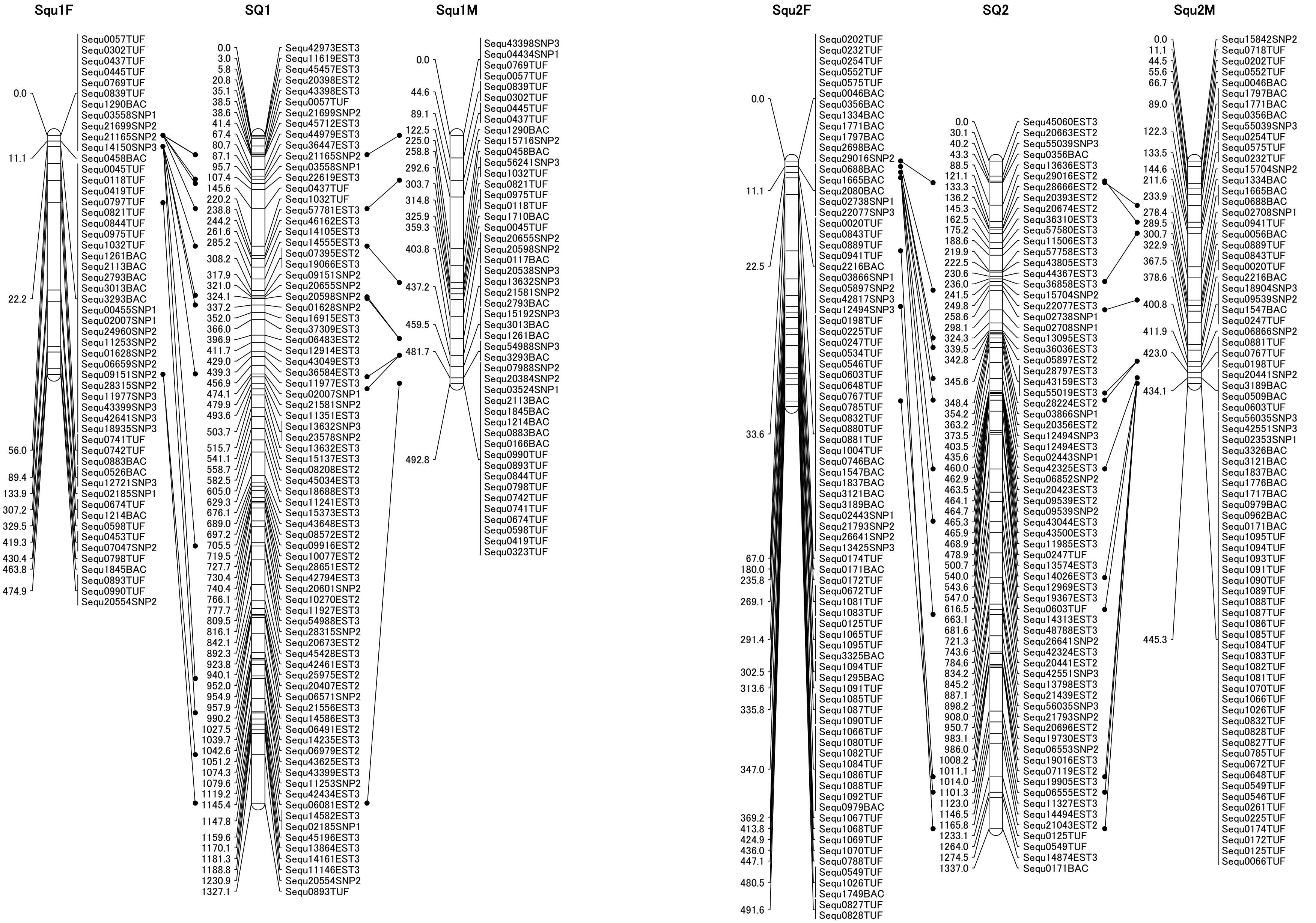
Squ1F
SQ1
Squ1M
Squ2F
SQ2
Squ2M
0.0
11.1
22.2
56.0
89.4
133.9
307.2
329.5
419.3
430.4
463.8
474.9
Sequ0057TUF
Sequ0302TUF
Sequ0437TUF
Sequ0445TUF
Sequ0769TUF
Sequ0839TUF
Sequ1290BAC
Sequ03558SNP1
Sequ21699SNP2
Sequ21165SNP2
Sequ14150SNP3
Sequ0458BAC
Sequ0045TUF
Sequ0118TUF
Sequ0419TUF
Sequ0797TUF
Sequ0821TUF
Sequ0844TUF
Sequ0975TUF
Sequ1032TUF
Sequ1261BAC
Sequ2113BAC
Sequ2793BAC
Sequ3013BAC
Sequ3293BAC
Sequ00455SNP1
Sequ02007SNP1
Sequ24960SNP2
Sequ11253SNP2
Sequ01628SNP2
Sequ06659SNP2
Sequ09151SNP2
Sequ28315SNP2
Sequ11977SNP3
Sequ43399SNP3
Sequ42641SNP3
Sequ18935SNP3
Sequ0741TUF
Sequ0742TUF
Sequ0883BAC
Sequ0526BAC
Sequ12721SNP3
Sequ02185SNP1
Sequ0674TUF
Sequ1214BAC
Sequ0598TUF
Sequ0453TUF
Sequ07047SNP2
Sequ0798TUF
Sequ1845BAC
Sequ0893TUF
Sequ0990TUF
Sequ20554SNP2
0.0
3.0
5.8
20.8
35.1
38.5
38.6
41.4
67.4
80.7
87.1
95.7
107.4
145.6
220.2
238.8
244.2
261.6
285.2
308.2
317.9
321.0
324.1
337.2
352.0
366.0
396.9
411.7
429.0
439.3
456.9
474.1
479.9
493.6
503.7
515.7
541.1
558.7
582.5
605.0
629.3
676.1
689.0
697.2
705.5
719.5
727.7
730.4
740.4
766.1
777.7
809.5
816.1
842.1
892.3
923.8
940.1
952.0
954.9
957.9
990.2
1027.5
1039.7
1042.6
1051.2
1074.3
1079.6
1119.2
1145.4
1147.8
1159.6
1170.1
1181.3
1188.8
1230.9
1327.1
Sequ42973EST3
Sequ11619EST3
Sequ45457EST3
Sequ20398EST2
Sequ43398EST3
Sequ0057TUF
Sequ21699SNP2
Sequ45712EST3
Sequ44979EST3
Sequ36447EST3
Sequ21165SNP2
Sequ03558SNP1
Sequ22619EST3
Sequ0437TUF
Sequ1032TUF
Sequ57781EST3
Sequ46162EST3
Sequ14105EST3
Sequ14555EST3
Sequ07395EST2
Sequ19066EST3
Sequ09151SNP2
Sequ20655SNP2
Sequ20598SNP2
Sequ01628SNP2
Sequ16915EST3
Sequ37309EST3
Sequ06483EST2
Sequ12914EST3
Sequ43049EST3
Sequ36584EST3
Sequ11977EST3
Sequ02007SNP1
Sequ21581SNP2
Sequ11351EST3
Sequ13632SNP3
Sequ23578SNP2
Sequ13632EST3
Sequ15137EST3
Sequ08208EST2
Sequ45034EST3
Sequ18688EST3
Sequ11241EST3
Sequ15373EST3
Sequ43648EST3
Sequ08572EST2
Sequ09916EST2
Sequ10077EST2
Sequ28651EST2
Sequ42794EST3
Sequ20601SNP2
Sequ10270EST2
Sequ11927EST3
Sequ54988EST3
Sequ28315SNP2
Sequ20673EST2
Sequ45428EST3
Sequ42461EST3
Sequ25975EST2
Sequ20407EST2
Sequ06571SNP2
Sequ21556EST3
Sequ14586EST3
Sequ06491EST2
Sequ14235EST3
Sequ06979EST2
Sequ43625EST3
Sequ43399EST3
Sequ11253SNP2
Sequ42434EST3
Sequ06081EST2
Sequ14582EST3
Sequ02185SNP1
Sequ45196EST3
Sequ13864EST3
Sequ14161EST3
Sequ11146EST3
Sequ20554SNP2
Sequ0893TUF
0.0
44.6
89.1
122.5
225.0
258.8
292.6
303.7
314.8
325.9
359.3
403.8
437.2
459.5
481.7
492.8
Sequ43398SNP3
Sequ04434SNP1
Sequ0769TUF
Sequ0057TUF
Sequ0839TUF
Sequ0302TUF
Sequ0445TUF
Sequ0437TUF
Sequ1290BAC
Sequ15716SNP2
Sequ0458BAC
Sequ56241SNP3
Sequ1032TUF
Sequ0821TUF
Sequ0975TUF
Sequ0118TUF
Sequ1710BAC
Sequ0045TUF
Sequ20655SNP2
Sequ20598SNP2
Sequ0117BAC
Sequ20538SNP3
Sequ13632SNP3
Sequ21581SNP2
Sequ2793BAC
Sequ15192SNP3
Sequ3013BAC
Sequ1261BAC
Sequ54988SNP3
Sequ3293BAC
Sequ07988SNP2
Sequ20384SNP2
Sequ03524SNP1
Sequ2113BAC
Sequ1845BAC
Sequ1214BAC
Sequ0883BAC
Sequ0166BAC
Sequ0990TUF
Sequ0893TUF
Sequ0844TUF
Sequ0798TUF
Sequ0742TUF
Sequ0741TUF
Sequ0674TUF
Sequ0598TUF
Sequ0419TUF
Sequ0323TUF
0.0
11.1
22.5
33.6
67.0
180.0
235.8
269.1
291.4
302.5
313.6
335.8
347.0
369.2
413.8
424.9
436.0
447.1
480.5
491.6
Sequ0202TUF
Sequ0232TUF
Sequ0254TUF
Sequ0552TUF
Sequ0575TUF
Sequ0046BAC
Sequ0356BAC
Sequ1334BAC
Sequ1771BAC
Sequ1797BAC
Sequ2698BAC
Sequ29016SNP2
Sequ0688BAC
Sequ1665BAC
Sequ2080BAC
Sequ02738SNP1
Sequ22077SNP3
Sequ0020TUF
Sequ0843TUF
Sequ0889TUF
Sequ0941TUF
Sequ2216BAC
Sequ03866SNP1
Sequ05897SNP2
Sequ42817SNP3
Sequ12494SNP3
Sequ0198TUF
Sequ0225TUF
Sequ0247TUF
Sequ0534TUF
Sequ0546TUF
Sequ0603TUF
Sequ0648TUF
Sequ0767TUF
Sequ0785TUF
Sequ0832TUF
Sequ0880TUF
Sequ0881TUF
Sequ1004TUF
Sequ0746BAC
Sequ1547BAC
Sequ1837BAC
Sequ3121BAC
Sequ3189BAC
Sequ02443SNP1
Sequ21793SNP2
Sequ26641SNP2
Sequ13425SNP3
Sequ0174TUF
Sequ0171BAC
Sequ0172TUF
Sequ0672TUF
Sequ1081TUF
Sequ1083TUF
Sequ0125TUF
Sequ1065TUF
Sequ1095TUF
Sequ3325BAC
Sequ1094TUF
Sequ1295BAC
Sequ1091TUF
Sequ1085TUF
Sequ1087TUF
Sequ1090TUF
Sequ1066TUF
Sequ1080TUF
Sequ1082TUF
Sequ1084TUF
Sequ1086TUF
Sequ1088TUF
Sequ1092TUF
Sequ0979BAC
Sequ1067TUF
Sequ1068TUF
Sequ1069TUF
Sequ1070TUF
Sequ0788TUF
Sequ0549TUF
Sequ1026TUF
Sequ1749BAC
Sequ0827TUF
Sequ0828TUF
0.0
30.1
40.2
43.3
88.5
121.1
133.3
136.2
145.3
162.5
175.2
188.6
219.9
222.5
230.6
236.0
241.5
249.8
258.6
298.1
324.3
339.5
342.8
345.6
348.4
354.2
363.2
373.5
403.5
435.6
460.0
462.9
463.5
464.1
464.7
465.3
465.9
468.9
478.9
500.7
540.0
543.6
547.0
616.5
663.1
681.6
721.3
743.6
784.6
834.2
845.2
887.1
898.2
908.0
950.7
983.1
986.0
1008.2
1011.1
1014.0
1101.3
1123.0
1146.5
1165.8
1233.1
1264.0
1274.5
1337.0
Sequ45060EST3
Sequ20663EST2
Sequ55039SNP3
Sequ0356BAC
Sequ13636EST3
Sequ29016EST2
Sequ28666EST2
Sequ20393EST2
Sequ20674EST2
Sequ36310EST3
Sequ57580EST3
Sequ11506EST3
Sequ57758EST3
Sequ43805EST3
Sequ44367EST3
Sequ36858EST3
Sequ15704SNP2
Sequ22077EST3
Sequ02738SNP1
Sequ02708SNP1
Sequ13095EST3
Sequ36036EST3
Sequ05897EST2
Sequ28797EST3
Sequ43159EST3
Sequ55019EST3
Sequ28224EST2
Sequ03866SNP1
Sequ20356EST2
Sequ12494SNP3
Sequ12494EST3
Sequ02443SNP1
Sequ42325EST3
Sequ06852SNP2
Sequ20423EST3
Sequ09539EST2
Sequ09539SNP2
Sequ43044EST3
Sequ43500EST3
Sequ11985EST3
Sequ0247TUF
Sequ13574EST3
Sequ14026EST3
Sequ12969EST3
Sequ19367EST3
Sequ0603TUF
Sequ14313EST3
Sequ48788EST3
Sequ26641SNP2
Sequ42324EST3
Sequ20441EST2
Sequ42551SNP3
Sequ13798EST3
Sequ21439EST2
Sequ56035SNP3
Sequ21793SNP2
Sequ20696EST2
Sequ19730EST3
Sequ06553SNP2
Sequ19016EST3
Sequ07119EST2
Sequ19905EST3
Sequ06555EST2
Sequ11327EST3
Sequ14494EST3
Sequ21043EST2
Sequ0125TUF
Sequ0549TUF
Sequ14874EST3
Sequ0171BAC
0.0
11.1
44.5
55.6
66.7
89.0
122.3
133.5
144.6
211.6
233.9
278.4
289.5
300.7
322.9
367.5
378.6
400.8
411.9
423.0
434.1
445.3
Sequ15842SNP2
Sequ0718TUF
Sequ0202TUF
Sequ0552TUF
Sequ0046BAC
Sequ1797BAC
Sequ1771BAC
Sequ0356BAC
Sequ55039SNP3
Sequ0254TUF
Sequ0575TUF
Sequ0232TUF
Sequ15704SNP2
Sequ1334BAC
Sequ1665BAC
Sequ0688BAC
Sequ02708SNP1
Sequ0941TUF
Sequ0056BAC
Sequ0889TUF
Sequ0843TUF
Sequ0020TUF
Sequ2216BAC
Sequ18904SNP3
Sequ09539SNP2
Sequ1547BAC
Sequ0247TUF
Sequ06866SNP2
Sequ0881TUF
Sequ0767TUF
Sequ0198TUF
Sequ20441SNP2
Sequ3189BAC
Sequ0509BAC
Sequ0603TUF
Sequ56035SNP3
Sequ42551SNP3
Sequ02353SNP1
Sequ3326BAC
Sequ3121BAC
Sequ1837BAC
Sequ1776BAC
Sequ1717BAC
Sequ0979BAC
Sequ0962BAC
Sequ0171BAC
Sequ1095TUF
Sequ1094TUF
Sequ1093TUF
Sequ1091TUF
Sequ1090TUF
Sequ1089TUF
Sequ1088TUF
Sequ1087TUF
Sequ1086TUF
Sequ1085TUF
Sequ1084TUF
Sequ1083TUF
Sequ1082TUF
Sequ1081TUF
Sequ1070TUF
Sequ1066TUF
Sequ1026TUF
Sequ0832TUF
Sequ0828TUF
Sequ0827TUF
Sequ0785TUF
Sequ0672TUF
Sequ0648TUF
Sequ0549TUF
Sequ0546TUF
Sequ0261TUF
Sequ0225TUF
Sequ0174TUF
Sequ0172TUF
Sequ0125TUF
Sequ0066TUF

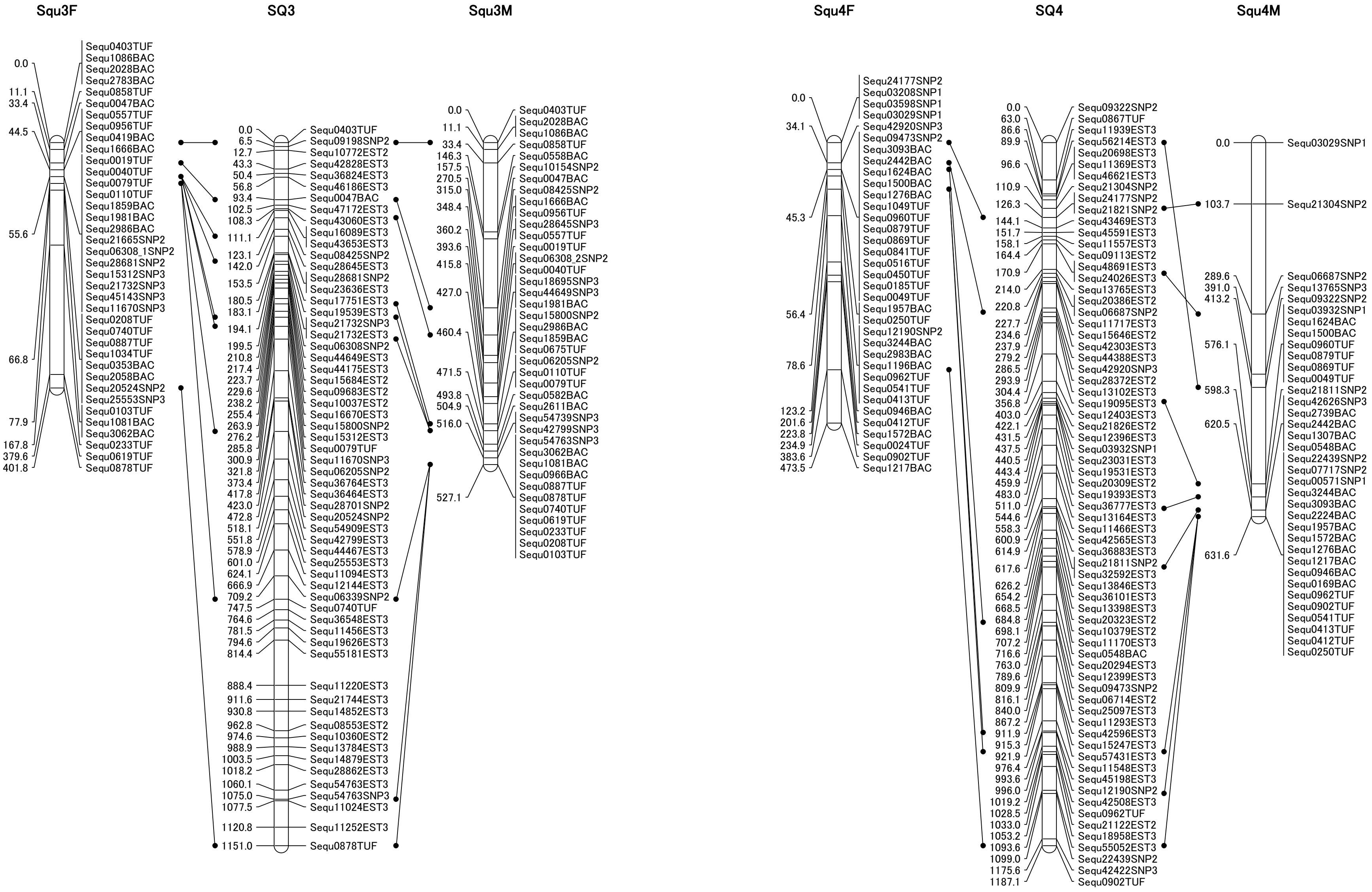
Squ3F
0.0
11.1
33.4
44.5
55.6
66.8
77.9
167.8
379.6
401.8
Sequ0403TUF
Sequ1086BAC
Sequ2028BAC
Sequ2783BAC
Sequ0858TUF
Sequ0047BAC
Sequ0557TUF
Sequ0956TUF
Sequ0419BAC
Sequ1666BAC
Sequ0019TUF
Sequ0040TUF
Sequ0079TUF
Sequ0110TUF
Sequ1859BAC
Sequ1981BAC
Sequ2986BAC
Sequ21665SNP2
Sequ06308_1SNP2
Sequ28681SNP2
Sequ15312SNP3
Sequ21732SNP3
Sequ45143SNP3
Sequ11670SNP3
Sequ0208TUF
Sequ0740TUF
Sequ0887TUF
Sequ1034TUF
Sequ0353BAC
Sequ2058BAC
Sequ20524SNP2
Sequ25553SNP3
Sequ0103TUF
Sequ1081BAC
Sequ3062BAC
Sequ0233TUF
Sequ0619TUF
Sequ0878TUF
SQ3
0.0
6.5
12.7
43.3
50.4
56.8
93.4
102.5
108.3
111.1
123.1
142.0
153.5
180.5
183.1
194.1
199.5
210.8
217.4
223.7
229.6
238.2
255.4
263.9
276.2
285.8
300.9
321.8
373.4
417.8
423.0
472.8
518.1
551.8
578.9
601.0
624.1
666.9
709.2
747.5
764.6
781.5
794.6
814.4
888.4
911.6
930.8
962.8
974.6
988.9
1003.5
1018.2
1060.1
1075.0
1077.5
1120.8
1151.0
Sequ0403TUF
Sequ09198SNP2
Sequ10772EST2
Sequ42828EST3
Sequ36824EST3
Sequ46186EST3
Sequ0047BAC
Sequ47172EST3
Sequ43060EST3
Sequ16089EST3
Sequ43653EST3
Sequ08425SNP2
Sequ28645EST3
Sequ28681SNP2
Sequ23636EST3
Sequ17751EST3
Sequ19539EST3
Sequ21732SNP3
Sequ21732EST3
Sequ06308SNP2
Sequ44649EST3
Sequ44175EST3
Sequ15684EST2
Sequ09683EST2
Sequ10037EST2
Sequ16670EST3
Sequ15800SNP2
Sequ15312EST3
Sequ0079TUF
Sequ11670SNP3
Sequ06205SNP2
Sequ36764EST3
Sequ36464EST3
Sequ28701SNP2
Sequ20524SNP2
Sequ54909EST3
Sequ42799EST3
Sequ44467EST3
Sequ25553EST3
Sequ11094EST3
Sequ12144EST3
Sequ06339SNP2
Sequ0740TUF
Sequ36548EST3
Sequ11456EST3
Sequ19626EST3
Sequ55181EST3
Sequ11220EST3
Sequ21744EST3
Sequ14852EST3
Sequ08553EST2
Sequ10360EST2
Sequ13784EST3
Sequ14879EST3
Sequ28862EST3
Sequ54763EST3
Sequ54763SNP3
Sequ11024EST3
Sequ11252EST3
Sequ0878TUF
Squ3M
0.0
11.1
33.4
146.3
157.5
270.5
315.0
348.4
360.2
393.6
415.8
427.0
460.4
471.5
493.8
504.9
516.0
527.1
Sequ0403TUF
Sequ2028BAC
Sequ1086BAC
Sequ0858TUF
Sequ0558BAC
Sequ10154SNP2
Sequ0047BAC
Sequ08425SNP2
Sequ1666BAC
Sequ0956TUF
Sequ28645SNP3
Sequ0557TUF
Sequ0019TUF
Sequ06308_2SNP2
Sequ0040TUF
Sequ18695SNP3
Sequ44649SNP3
Sequ1981BAC
Sequ15800SNP2
Sequ2986BAC
Sequ1859BAC
Sequ0675TUF
Sequ06205SNP2
Sequ0110TUF
Sequ0079TUF
Sequ0582BAC
Sequ2611BAC
Sequ54739SNP3
Sequ42799SNP3
Sequ54763SNP3
Sequ3062BAC
Sequ1081BAC
Sequ0966BAC
Sequ0887TUF
Sequ0878TUF
Sequ0740TUF
Sequ0619TUF
Sequ0233TUF
Sequ0208TUF
Sequ0103TUF
Squ4F
0.0
34.1
45.3
56.4
78.6
123.2
201.6
223.8
234.9
383.6
473.5
Sequ24177SNP2
Sequ03208SNP1
Sequ03598SNP1
Sequ03029SNP1
Sequ42920SNP3
Sequ09473SNP2
Sequ3093BAC
Sequ2442BAC
Sequ1624BAC
Sequ1500BAC
Sequ1276BAC
Sequ1049TUF
Sequ0960TUF
Sequ0879TUF
Sequ0869TUF
Sequ0841TUF
Sequ0516TUF
Sequ0450TUF
Sequ0185TUF
Sequ0049TUF
Sequ1957BAC
Sequ0250TUF
Sequ12190SNP2
Sequ3244BAC
Sequ2983BAC
Sequ1196BAC
Sequ0962TUF
Sequ0541TUF
Sequ0413TUF
Sequ0946BAC
Sequ0412TUF
Sequ1572BAC
Sequ0024TUF
Sequ0902TUF
Sequ1217BAC
SQ4
0.0
63.0
86.6
89.9
96.6
110.9
126.3
144.1
151.7
158.1
164.4
170.9
214.0
220.8
227.7
234.6
237.9
279.2
286.5
293.9
304.4
356.8
403.0
422.1
431.5
437.5
440.5
443.4
459.9
483.0
511.0
544.6
558.3
600.9
614.9
617.6
626.2
654.2
668.5
684.8
698.1
707.2
716.6
763.0
789.6
809.9
816.1
840.0
867.2
911.9
915.3
921.9
976.4
993.6
996.0
1019.2
1028.5
1033.0
1053.2
1093.6
1099.0
1175.6
1187.1
Sequ09322SNP2
Sequ0867TUF
Sequ11939EST3
Sequ56214EST3
Sequ20698EST3
Sequ11369EST3
Sequ46621EST3
Sequ21304SNP2
Sequ24177SNP2
Sequ21821SNP2
Sequ43469EST3
Sequ45591EST3
Sequ11557EST3
Sequ09113EST2
Sequ48691EST3
Sequ24026EST3
Sequ13765EST3
Sequ20386EST2
Sequ06687SNP2
Sequ11717EST3
Sequ15646EST2
Sequ42303EST3
Sequ44388EST3
Sequ42920SNP3
Sequ28372EST2
Sequ13102EST3
Sequ19095EST3
Sequ12403EST3
Sequ21826EST2
Sequ12396EST3
Sequ03932SNP1
Sequ23031EST3
Sequ19531EST3
Sequ20309EST2
Sequ19393EST3
Sequ36777EST3
Sequ13164EST3
Sequ11466EST3
Sequ42565EST3
Sequ36883EST3
Sequ21811SNP2
Sequ32592EST3
Sequ13846EST3
Sequ36101EST3
Sequ13398EST3
Sequ20323EST2
Sequ10379EST2
Sequ11170EST3
Sequ0548BAC
Sequ20294EST3
Sequ12399EST3
Sequ09473SNP2
Sequ06714EST2
Sequ25097EST3
Sequ11293EST3
Sequ42596EST3
Sequ15247EST3
Sequ57431EST3
Sequ11548EST3
Sequ45198EST3
Sequ12190SNP2
Sequ42508EST3
Sequ0962TUF
Sequ21122EST2
Sequ18958EST3
Sequ55052EST3
Sequ22439SNP2
Sequ42422SNP3
Sequ0902TUF
Squ4M
0.0
103.7
289.6
391.0
413.2
576.1
598.3
620.5
631.6
Sequ03029SNP1
Sequ21304SNP2
Sequ06687SNP2
Sequ13765SNP3
Sequ09322SNP2
Sequ03932SNP1
Sequ1624BAC
Sequ1500BAC
Sequ0960TUF
Sequ0879TUF
Sequ0869TUF
Sequ0049TUF
Sequ21811SNP2
Sequ42626SNP3
Sequ2739BAC
Sequ2442BAC
Sequ1307BAC
Sequ0548BAC
Sequ22439SNP2
Sequ07717SNP2
Sequ00571SNP1
Sequ3244BAC
Sequ3093BAC
Sequ2224BAC
Sequ1957BAC
Sequ1572BAC
Sequ1276BAC
Sequ1217BAC
Sequ0946BAC
Sequ0169BAC
Sequ0962TUF
Sequ0902TUF
Sequ0541TUF
Sequ0413TUF
Sequ0412TUF
Sequ0250TUF

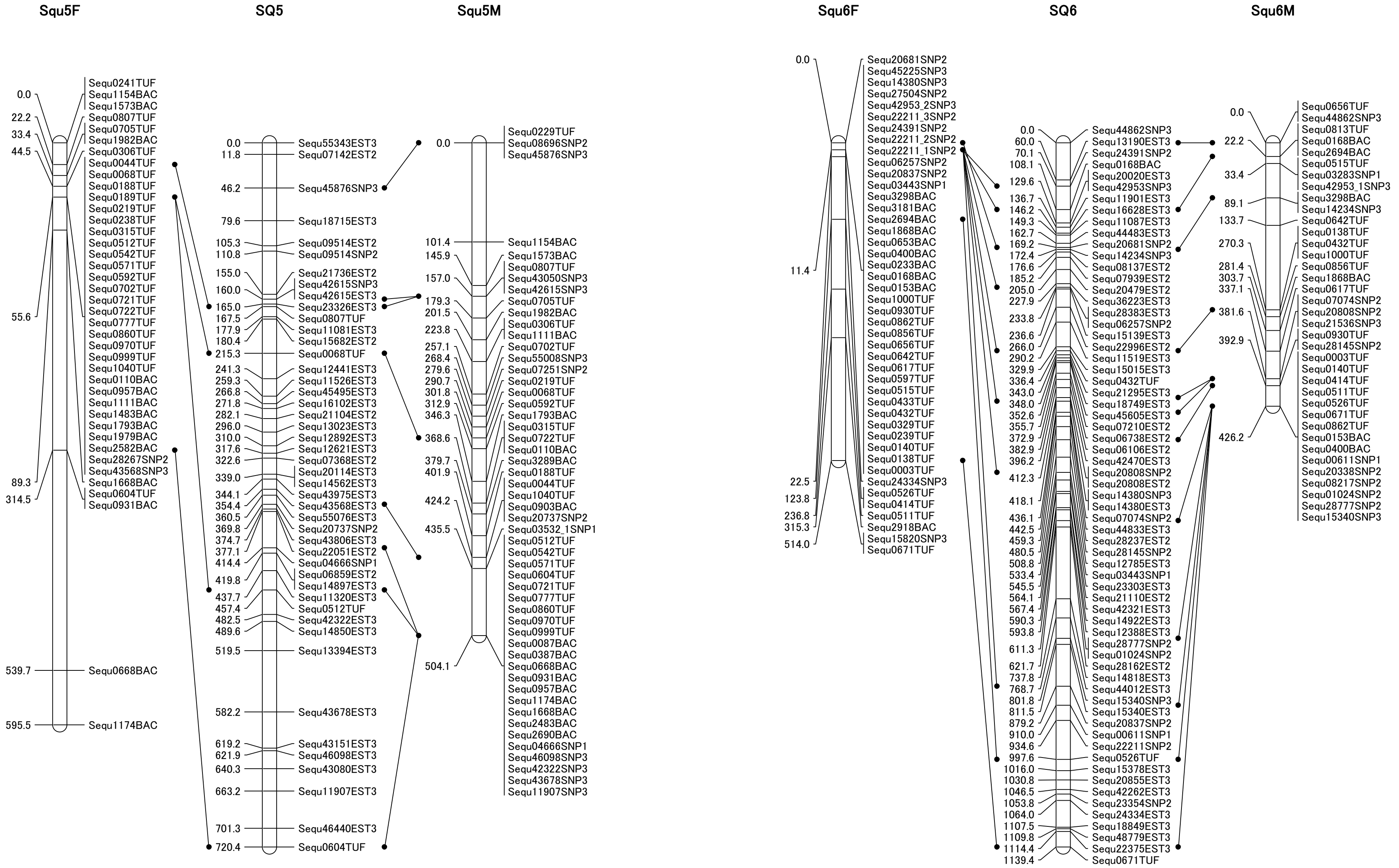
Squ5F
0.0
22.2
33.4
44.5
55.6
89.3
314.5
539.7
595.5
Sequ0241TUF
Sequ1154BAC
Sequ1573BAC
Sequ0807TUF
Sequ0705TUF
Sequ1982BAC
Sequ0306TUF
Sequ0044TUF
Sequ0068TUF
Sequ0188TUF
Sequ0189TUF
Sequ0219TUF
Sequ0238TUF
Sequ0315TUF
Sequ0512TUF
Sequ0542TUF
Sequ0571TUF
Sequ0592TUF
Sequ0702TUF
Sequ0721TUF
Sequ0722TUF
Sequ0777TUF
Sequ0860TUF
Sequ0970TUF
Sequ0999TUF
Sequ1040TUF
Sequ0110BAC
Sequ0957BAC
Sequ1111BAC
Sequ1483BAC
Sequ1793BAC
Sequ1979BAC
Sequ2582BAC
Sequ28267SNP2
Sequ43568SNP3
Sequ1668BAC
Sequ0604TUF
Sequ0931BAC
Sequ0668BAC
Sequ1174BAC
SQ5
0.0 Sequ55343EST3
11.8 Sequ07142EST2
46.2 Sequ45876SNP3
79.6 Sequ18715EST3
105.3 Sequ09514EST2
110.8 Sequ09514SNP2
155.0 Sequ21736EST2
Sequ42615SNP3
160.0 Sequ42615EST3
165.0 Sequ23326EST3
167.5 Sequ0807TUF
177.9 Sequ11081EST3
180.4 Sequ15682EST2
215.3 Sequ0068TUF
241.3 Sequ12441EST3
259.3 Sequ11526EST3
266.8 Sequ45495EST3
271.8 Sequ16102EST3
282.1 Sequ21104EST2
296.0 Sequ13023EST3
310.0 Sequ12892EST3
317.6 Sequ12621EST3
322.6 Sequ07368EST2
339.0 Sequ20114EST3
Sequ14562EST3
344.1 Sequ43975EST3
354.4 Sequ43568EST3
360.5 Sequ55076EST3
369.8 Sequ20737SNP2
374.7 Sequ43806EST3
377.1 Sequ22051EST2
414.4 Sequ04666SNP1
419.8 Sequ06859EST2
Sequ14897EST3
437.7 Sequ11320EST3
457.4 Sequ0512TUF
482.5 Sequ42322EST3
489.6 Sequ14850EST3
519.5 Sequ13394EST3
582.2 Sequ43678EST3
619.2 Sequ43151EST3
621.9 Sequ46098EST3
640.3 Sequ43080EST3
663.2 Sequ11907EST3
701.3 Sequ46440EST3
720.4 Sequ0604TUF
Squ5M
0.0
101.4
145.9
157.0
179.3
201.5
223.8
257.1
268.4
279.6
290.7
301.8
312.9
346.3
368.6
379.7
401.9
424.2
435.5
504.1
Sequ0229TUF
Sequ08696SNP2
Sequ45876SNP3
Sequ1154BAC
Sequ1573BAC
Sequ0807TUF
Sequ43050SNP3
Sequ42615SNP3
Sequ0705TUF
Sequ1982BAC
Sequ0306TUF
Sequ1111BAC
Sequ0702TUF
Sequ55008SNP3
Sequ07251SNP2
Sequ0219TUF
Sequ0068TUF
Sequ0592TUF
Sequ1793BAC
Sequ0315TUF
Sequ0722TUF
Sequ0110BAC
Sequ3289BAC
Sequ0188TUF
Sequ0044TUF
Sequ1040TUF
Sequ0903BAC
Sequ20737SNP2
Sequ03532_1SNP1
Sequ0512TUF
Sequ0542TUF
Sequ0571TUF
Sequ0604TUF
Sequ0721TUF
Sequ0777TUF
Sequ0860TUF
Sequ0970TUF
Sequ0999TUF
Sequ0087BAC
Sequ0387BAC
Sequ0668BAC
Sequ0931BAC
Sequ0957BAC
Sequ1174BAC
Sequ1668BAC
Sequ2483BAC
Sequ2690BAC
Sequ04666SNP1
Sequ46098SNP3
Sequ42322SNP3
Sequ43678SNP3
Sequ11907SNP3
Squ6F
0.0
11.4
22.5
123.8
236.8
315.3
514.0
Sequ20681SNP2
Sequ45225SNP3
Sequ14380SNP3
Sequ27504SNP2
Sequ42953_2SNP3
Sequ22211_3SNP2
Sequ24391SNP2
Sequ22211_2SNP2
Sequ22211_1SNP2
Sequ06257SNP2
Sequ20837SNP2
Sequ03443SNP1
Sequ3298BAC
Sequ3181BAC
Sequ2694BAC
Sequ1868BAC
Sequ0653BAC
Sequ0400BAC
Sequ0233BAC
Sequ0168BAC
Sequ0153BAC
Sequ1000TUF
Sequ0930TUF
Sequ0862TUF
Sequ0856TUF
Sequ0656TUF
Sequ0642TUF
Sequ0617TUF
Sequ0597TUF
Sequ0515TUF
Sequ0433TUF
Sequ0432TUF
Sequ0329TUF
Sequ0239TUF
Sequ0140TUF
Sequ0138TUF
Sequ0003TUF
Sequ24334SNP3
Sequ0526TUF
Sequ0414TUF
Sequ0511TUF
Sequ2918BAC
Sequ15820SNP3
Sequ0671TUF
SQ6
0.0 Sequ44862SNP3
60.0 Sequ13190EST3
70.1 Sequ24391SNP2
108.1 Sequ0168BAC
Sequ20020EST3
129.6 Sequ42953SNP3
136.7 Sequ11901EST3
146.2 Sequ16628EST3
149.3 Sequ11087EST3
162.7 Sequ44483EST3
169.2 Sequ20681SNP2
172.4 Sequ14234SNP3
176.6 Sequ08137EST2
185.2 Sequ07939EST2
205.0 Sequ20479EST2
227.9 Sequ36223EST3
Sequ28383EST3
233.8 Sequ06257SNP2
236.6 Sequ15139EST3
266.0 Sequ22996EST2
290.2 Sequ11519EST3
329.9 Sequ15015EST3
336.4 Sequ0432TUF
343.0 Sequ21295EST3
348.0 Sequ18749EST3
352.6 Sequ45605EST3
355.7 Sequ07210EST2
372.9 Sequ06738EST2
382.9 Sequ06106EST2
396.2 Sequ42470EST3
Sequ20808SNP2
412.3 Sequ20808EST2
Sequ14380SNP3
418.1 Sequ14380EST3
436.1 Sequ07074SNP2
442.5 Sequ44833EST3
459.3 Sequ28237EST2
480.5 Sequ28145SNP2
508.8 Sequ12785EST3
533.4 Sequ03443SNP1
545.5 Sequ23303EST3
564.1 Sequ21110EST2
567.4 Sequ42321EST3
590.3 Sequ14922EST3
593.8 Sequ12388EST3
Sequ28777SNP2
611.3 Sequ01024SNP2
621.7 Sequ28162EST2
737.8 Sequ14818EST3
768.7 Sequ44012EST3
801.8 Sequ15340SNP3
811.5 Sequ15340EST3
879.2 Sequ20837SNP2
910.0 Sequ00611SNP1
934.6 Sequ22211SNP2
997.6 Sequ0526TUF
1016.0 Sequ15378EST3
1030.8 Sequ20855EST3
1046.5 Sequ42262EST3
1053.8 Sequ23354SNP2
1064.0 Sequ24334EST3
1107.5 Sequ18849EST3
1109.8 Sequ48779EST3
1114.4 Sequ22375EST3
1139.4 Sequ0671TUF
Squ6M
0.0
22.2
33.4
89.1
133.7
270.3
281.4
303.7
337.1
381.6
392.9
426.2
Sequ0656TUF
Sequ44862SNP3
Sequ0813TUF
Sequ0168BAC
Sequ2694BAC
Sequ0515TUF
Sequ03283SNP1
Sequ42953_1SNP3
Sequ3298BAC
Sequ14234SNP3
Sequ0642TUF
Sequ0138TUF
Sequ0432TUF
Sequ1000TUF
Sequ0856TUF
Sequ1868BAC
Sequ0617TUF
Sequ07074SNP2
Sequ20808SNP2
Sequ21536SNP3
Sequ0930TUF
Sequ28145SNP2
Sequ0003TUF
Sequ0140TUF
Sequ0414TUF
Sequ0511TUF
Sequ0526TUF
Sequ0671TUF
Sequ0862TUF
Sequ0153BAC
Sequ0400BAC
Sequ00611SNP1
Sequ20338SNP2
Sequ08217SNP2
Sequ01024SNP2
Sequ28777SNP2
Sequ15340SNP3

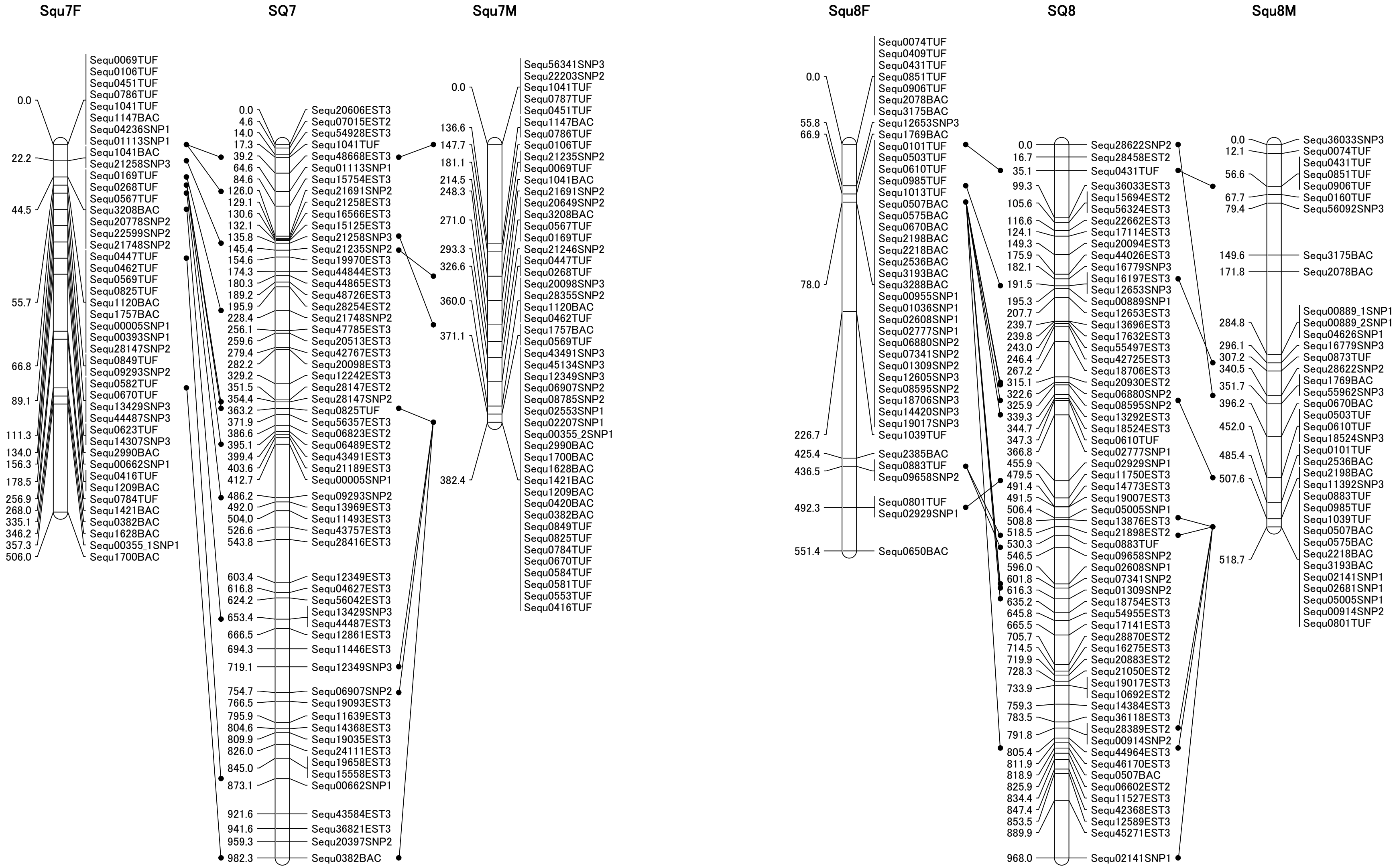
Squ7F
0.0
22.2
44.5
55.7
66.8
89.1
111.3
134.0
156.3
178.5
256.9
268.0
335.1
346.2
357.3
506.0
Sequ0069TUF
Sequ0106TUF
Sequ0451TUF
Sequ0786TUF
Sequ1041TUF
Sequ1147BAC
Sequ04236SNP1
Sequ01113SNP1
Sequ1041BAC
Sequ21258SNP3
Sequ0169TUF
Sequ0268TUF
Sequ0567TUF
Sequ3208BAC
Sequ20778SNP2
Sequ22599SNP2
Sequ21748SNP2
Sequ0447TUF
Sequ0462TUF
Sequ0569TUF
Sequ0825TUF
Sequ1120BAC
Sequ1757BAC
Sequ00005SNP1
Sequ00393SNP1
Sequ28147SNP2
Sequ0849TUF
Sequ09293SNP2
Sequ0582TUF
Sequ0670TUF
Sequ13429SNP3
Sequ44487SNP3
Sequ0623TUF
Sequ14307SNP3
Sequ2990BAC
Sequ00662SNP1
Sequ0416TUF
Sequ1209BAC
Sequ0784TUF
Sequ1421BAC
Sequ0382BAC
Sequ1628BAC
Sequ00355_1SNP1
Sequ1700BAC
SQ7
0.0 Sequ20606EST3
4.6 Sequ07015EST2
14.0 Sequ54928EST3
17.3 Sequ1041TUF
39.2 Sequ48668EST3
64.6 Sequ01113SNP1
84.6 Sequ15754EST3
126.0 Sequ21691SNP2
129.1 Sequ21258EST3
130.6 Sequ16566EST3
132.1 Sequ15125EST3
135.8 Sequ21258SNP3
145.4 Sequ21235SNP2
154.6 Sequ19970EST3
174.3 Sequ44844EST3
180.3 Sequ44865EST3
189.2 Sequ48726EST3
195.9 Sequ28254EST2
228.4 Sequ21748SNP2
256.1 Sequ47785EST3
259.6 Sequ20513EST3
279.4 Sequ42767EST3
282.2 Sequ20098EST3
329.2 Sequ12242EST3
351.5 Sequ28147EST2
354.4 Sequ28147SNP2
363.2 Sequ0825TUF
371.9 Sequ56357EST3
386.6 Sequ06823EST2
395.1 Sequ06489EST2
399.4 Sequ43491EST3
403.6 Sequ21189EST3
412.7 Sequ00005SNP1
486.2 Sequ09293SNP2
492.0 Sequ13969EST3
504.0 Sequ11493EST3
526.6 Sequ43757EST3
543.8 Sequ28416EST3
603.4 Sequ12349EST3
616.8 Sequ04627EST3
624.2 Sequ56042EST3
653.4 Sequ13429SNP3
Sequ44487SNP3
666.5 Sequ12861EST3
694.3 Sequ11446EST3
719.1 Sequ12349SNP3
754.7 Sequ06907SNP2
766.5 Sequ19093EST3
795.9 Sequ11639EST3
804.6 Sequ14368EST3
809.9 Sequ19035EST3
826.0 Sequ24111EST3
845.0 Sequ19658EST3
Sequ15558EST3
873.1 Sequ00662SNP1
921.6 Sequ43584EST3
941.6 Sequ36821EST3
959.3 Sequ20397SNP2
982.3 Sequ0382BAC
Squ7M
0.0
136.6
147.7
181.1
214.5
248.3
271.0
293.3
326.6
360.0
371.1
382.4
Sequ56341SNP3
Sequ22203SNP2
Sequ1041TUF
Sequ0787TUF
Sequ0451TUF
Sequ1147BAC
Sequ0786TUF
Sequ0106TUF
Sequ21235SNP2
Sequ0069TUF
Sequ1041BAC
Sequ21691SNP2
Sequ20649SNP2
Sequ3208BAC
Sequ0567TUF
Sequ0169TUF
Sequ21246SNP2
Sequ0447TUF
Sequ0268TUF
Sequ20098SNP3
Sequ28355SNP2
Sequ1120BAC
Sequ0462TUF
Sequ1757BAC
Sequ0569TUF
Sequ43491SNP3
Sequ45134SNP3
Sequ12349SNP3
Sequ06907SNP2
Sequ08785SNP2
Sequ02553SNP1
Sequ02207SNP1
Sequ00355_2SNP1
Sequ2990BAC
Sequ1700BAC
Sequ1628BAC
Sequ1421BAC
Sequ1209BAC
Sequ0420BAC
Sequ0382BAC
Sequ0849TUF
Sequ0825TUF
Sequ0784TUF
Sequ0670TUF
Sequ0584TUF
Sequ0581TUF
Sequ0553TUF
Sequ0416TUF
Squ8F
0.0
55.8
66.9
78.0
226.7
425.4
436.5
492.3
551.4
Sequ0074TUF
Sequ0409TUF
Sequ0431TUF
Sequ0851TUF
Sequ0906TUF
Sequ2078BAC
Sequ3175BAC
Sequ12653SNP3
Sequ1769BAC
Sequ0101TUF
Sequ0503TUF
Sequ0610TUF
Sequ0985TUF
Sequ1013TUF
Sequ0507BAC
Sequ0575BAC
Sequ0670BAC
Sequ2198BAC
Sequ2218BAC
Sequ2536BAC
Sequ3193BAC
Sequ3288BAC
Sequ00955SNP1
Sequ01036SNP1
Sequ02608SNP1
Sequ02777SNP1
Sequ06880SNP2
Sequ07341SNP2
Sequ01309SNP2
Sequ12605SNP3
Sequ08595SNP2
Sequ18706SNP3
Sequ14420SNP3
Sequ19017SNP3
Sequ1039TUF
Sequ2385BAC
Sequ0883TUF
Sequ09658SNP2
Sequ0801TUF
Sequ02929SNP1
Sequ0650BAC
SQ8
0.0 Sequ28622SNP2
16.7 Sequ28458EST2
35.1 Sequ0431TUF
99.3 Sequ36033EST3
105.6 Sequ15694EST2
Sequ56324EST3
116.6 Sequ22662EST3
124.1 Sequ17114EST3
149.3 Sequ20094EST3
175.9 Sequ44026EST3
182.1 Sequ16779SNP3
191.5 Sequ16197EST3
Sequ12653SNP3
195.3 Sequ00889SNP1
207.7 Sequ12653EST3
239.7 Sequ13696EST3
239.8 Sequ17632EST3
243.0 Sequ55497EST3
246.4 Sequ42725EST3
267.2 Sequ18706EST3
315.1 Sequ20930EST2
322.6 Sequ06880SNP2
325.9 Sequ08595SNP2
339.3 Sequ13292EST3
344.7 Sequ18524EST3
347.3 Sequ0610TUF
366.8 Sequ02777SNP1
455.9 Sequ02929SNP1
479.5 Sequ11750EST3
491.4 Sequ14773EST3
491.5 Sequ19007EST3
506.4 Sequ05005SNP1
508.8 Sequ13876EST3
518.5 Sequ21898EST2
530.3 Sequ0883TUF
546.5 Sequ09658SNP2
596.0 Sequ02608SNP1
601.8 Sequ07341SNP2
616.3 Sequ01309SNP2
635.2 Sequ18754EST3
645.8 Sequ54955EST3
665.5 Sequ17141EST3
705.7 Sequ28870EST2
714.5 Sequ16275EST3
719.9 Sequ20883EST2
728.3 Sequ21050EST2
733.9 Sequ19017EST3
Sequ10692EST2
759.3 Sequ14384EST3
783.5 Sequ36118EST3
791.8 Sequ28389EST2
Sequ00914SNP2
805.4 Sequ44964EST3
811.9 Sequ46170EST3
818.9 Sequ0507BAC
825.9 Sequ06602EST2
834.4 Sequ11527EST3
847.4 Sequ42368EST3
853.5 Sequ12589EST3
889.9 Sequ45271EST3
968.0 Sequ02141SNP1
Squ8M
0.0 Sequ36033SNP3
12.1 Sequ0074TUF
56.6 Sequ0431TUF
Sequ0851TUF
Sequ0906TUF
67.7 Sequ0160TUF
79.4 Sequ56092SNP3
149.6 Sequ3175BAC
171.8 Sequ2078BAC
284.8 Sequ00889_1SNP1
Sequ00889_2SNP1
Sequ04626SNP1
296.1 Sequ16779SNP3
307.2 Sequ0873TUF
340.5 Sequ28622SNP2
351.7 Sequ1769BAC
Sequ55962SNP3
396.2 Sequ0670BAC
452.0 Sequ0503TUF
Sequ0610TUF
Sequ18524SNP3
485.4 Sequ0101TUF
Sequ2536BAC
507.6 Sequ2198BAC
Sequ11392SNP3
Sequ0883TUF
Sequ0985TUF
Sequ1039TUF
Sequ0507BAC
Sequ0575BAC
518.7 Sequ2218BAC
Sequ3193BAC
Sequ02141SNP1
Sequ02681SNP1
Sequ05005SNP1
Sequ00914SNP2
Sequ0801TUF

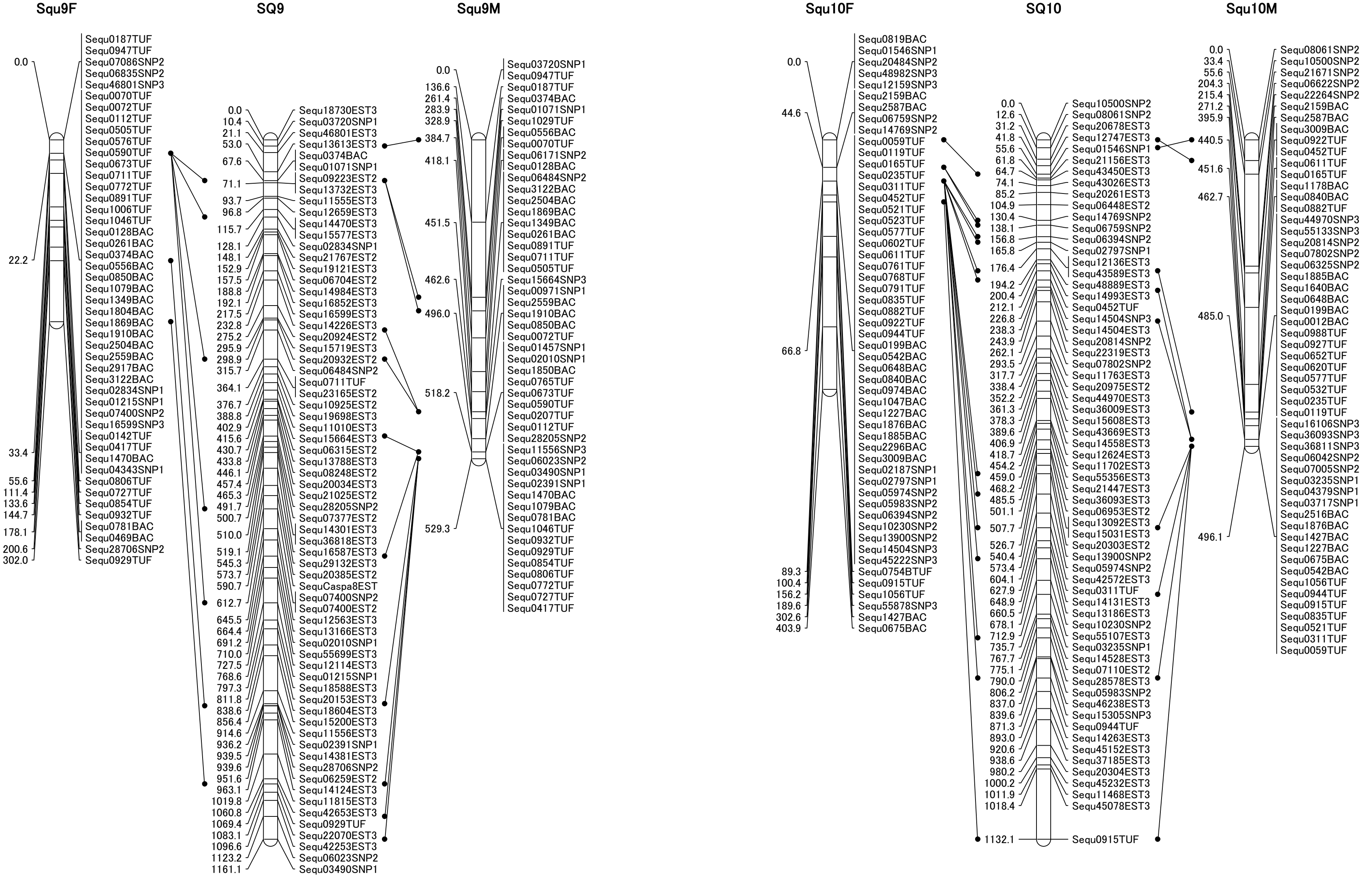
Squ9F
0.0
22.2
33.4
55.6
111.4
133.6
144.7
178.1
200.6
302.0
Sequ0187TUF
Sequ0947TUF
Sequ07086SNP2
Sequ06835SNP2
Sequ46801SNP3
Sequ0070TUF
Sequ0072TUF
Sequ0112TUF
Sequ0505TUF
Sequ0576TUF
Sequ0590TUF
Sequ0673TUF
Sequ0711TUF
Sequ0772TUF
Sequ0891TUF
Sequ1006TUF
Sequ1046TUF
Sequ0128BAC
Sequ0261BAC
Sequ0374BAC
Sequ0556BAC
Sequ0850BAC
Sequ1079BAC
Sequ1349BAC
Sequ1804BAC
Sequ1869BAC
Sequ1910BAC
Sequ2504BAC
Sequ2559BAC
Sequ2917BAC
Sequ3122BAC
Sequ02834SNP1
Sequ01215SNP1
Sequ07400SNP2
Sequ16599SNP3
Sequ0142TUF
Sequ0417TUF
Sequ1470BAC
Sequ04343SNP1
Sequ0806TUF
Sequ0727TUF
Sequ0854TUF
Sequ0932TUF
Sequ0781BAC
Sequ0469BAC
Sequ28706SNP2
Sequ0929TUF
SQ9
0.0 Sequ18730EST3
10.4 Sequ03720SNP1
21.1 Sequ46801EST3
53.0 Sequ13613EST3
67.6 Sequ0374BAC
Sequ01071SNP1
71.1 Sequ09223EST2
Sequ13732EST3
93.7 Sequ11555EST3
96.8 Sequ12659EST3
115.7 Sequ14470EST3
Sequ15577EST3
128.1 Sequ02834SNP1
148.1 Sequ21767EST2
152.9 Sequ19121EST3
157.5 Sequ06704EST2
188.8 Sequ14984EST3
192.1 Sequ16852EST3
217.5 Sequ16599EST3
232.8 Sequ14226EST3
275.2 Sequ20924EST2
295.9 Sequ15719EST3
298.9 Sequ20932EST2
315.7 Sequ06484SNP2
364.1 Sequ0711TUF
Sequ23165EST2
376.7 Sequ10925EST2
388.8 Sequ19698EST3
402.9 Sequ11010EST3
415.6 Sequ15664EST3
430.7 Sequ06315EST2
433.8 Sequ13788EST3
446.1 Sequ08248EST2
457.4 Sequ20034EST3
465.3 Sequ21025EST2
491.7 Sequ28205SNP2
500.7 Sequ07377EST2
510.0 Sequ14301EST3
Sequ36818EST3
519.1 Sequ16587EST3
545.3 Sequ29132EST3
573.7 Sequ20385EST2
590.7 SequCaspa8EST
Sequ07400SNP2
612.7 Sequ07400EST2
645.5 Sequ12563EST3
664.4 Sequ13166EST3
691.2 Sequ02010SNP1
710.0 Sequ55699EST3
727.5 Sequ12114EST3
768.6 Sequ01215SNP1
797.3 Sequ18588EST3
811.8 Sequ20153EST3
838.6 Sequ18604EST3
856.4 Sequ15200EST3
914.6 Sequ11556EST3
936.2 Sequ02391SNP1
939.5 Sequ14381EST3
939.6 Sequ28706SNP2
951.6 Sequ06259EST2
963.1 Sequ14124EST3
1019.8 Sequ11815EST3
1060.8 Sequ42653EST3
1069.4 Sequ0929TUF
1083.1 Sequ22070EST3
1096.6 Sequ42253EST3
1123.2 Sequ06023SNP2
1161.1 Sequ03490SNP1
Squ9M
0.0
136.6
261.4
283.9
328.9
384.7
418.1
451.5
462.6
496.0
518.2
529.3
Sequ03720SNP1
Sequ0947TUF
Sequ0187TUF
Sequ0374BAC
Sequ01071SNP1
Sequ1029TUF
Sequ0556BAC
Sequ0070TUF
Sequ06171SNP2
Sequ0128BAC
Sequ06484SNP2
Sequ3122BAC
Sequ2504BAC
Sequ1869BAC
Sequ1349BAC
Sequ0261BAC
Sequ0891TUF
Sequ0711TUF
Sequ0505TUF
Sequ15664SNP3
Sequ00971SNP1
Sequ2559BAC
Sequ1910BAC
Sequ0850BAC
Sequ0072TUF
Sequ01457SNP1
Sequ02010SNP1
Sequ1850BAC
Sequ0765TUF
Sequ0673TUF
Sequ0590TUF
Sequ0207TUF
Sequ0112TUF
Sequ28205SNP2
Sequ11556SNP3
Sequ06023SNP2
Sequ03490SNP1
Sequ02391SNP1
Sequ1470BAC
Sequ1079BAC
Sequ0781BAC
Sequ1046TUF
Sequ0932TUF
Sequ0929TUF
Sequ0854TUF
Sequ0806TUF
Sequ0772TUF
Sequ0727TUF
Sequ0417TUF
Squ10F
0.0
44.6
66.8
89.3
100.4
156.2
189.6
302.6
403.9
Sequ0819BAC
Sequ01546SNP1
Sequ20484SNP2
Sequ48982SNP3
Sequ12159SNP3
Sequ2159BAC
Sequ2587BAC
Sequ06759SNP2
Sequ14769SNP2
Sequ0059TUF
Sequ0119TUF
Sequ0165TUF
Sequ0235TUF
Sequ0311TUF
Sequ0452TUF
Sequ0521TUF
Sequ0523TUF
Sequ0577TUF
Sequ0602TUF
Sequ0611TUF
Sequ0761TUF
Sequ0768TUF
Sequ0791TUF
Sequ0835TUF
Sequ0882TUF
Sequ0922TUF
Sequ0944TUF
Sequ0199BAC
Sequ0542BAC
Sequ0648BAC
Sequ0840BAC
Sequ0974BAC
Sequ1047BAC
Sequ1227BAC
Sequ1876BAC
Sequ1885BAC
Sequ2296BAC
Sequ3009BAC
Sequ02187SNP1
Sequ02797SNP1
Sequ05974SNP2
Sequ05983SNP2
Sequ06394SNP2
Sequ10230SNP2
Sequ13900SNP2
Sequ14504SNP3
Sequ45222SNP3
Sequ0754BTUF
Sequ0915TUF
Sequ1056TUF
Sequ55878SNP3
Sequ1427BAC
Sequ0675BAC
SQ10
0.0 Sequ10500SNP2
12.6 Sequ08061SNP2
31.2 Sequ20678EST3
41.8 Sequ12747EST3
55.6 Sequ01546SNP1
61.8 Sequ21156EST3
64.7 Sequ43450EST3
74.1 Sequ43026EST3
85.2 Sequ20261EST3
104.9 Sequ06448EST2
130.4 Sequ14769SNP2
138.1 Sequ06759SNP2
156.8 Sequ06394SNP2
165.8 Sequ02797SNP1
176.4 Sequ12136EST3
Sequ43589EST3
194.2 Sequ48889EST3
200.4 Sequ14993EST3
212.1 Sequ0452TUF
226.8 Sequ14504SNP3
238.3 Sequ14504EST3
243.9 Sequ20814SNP2
262.1 Sequ22319EST3
293.5 Sequ07802SNP2
317.7 Sequ11763EST3
338.4 Sequ20975EST2
352.2 Sequ44970EST3
361.3 Sequ36009EST3
378.3 Sequ15608EST3
389.6 Sequ43669EST3
406.9 Sequ14558EST3
418.7 Sequ12624EST3
454.2 Sequ11702EST3
459.0 Sequ55356EST3
468.2 Sequ21447EST3
485.5 Sequ36093EST3
501.1 Sequ06953EST2
507.7 Sequ13092EST3
Sequ15031EST3
526.7 Sequ20303EST2
540.4 Sequ13900SNP2
573.4 Sequ05974SNP2
604.1 Sequ42572EST3
627.9 Sequ0311TUF
648.9 Sequ14131EST3
660.5 Sequ13186EST3
678.1 Sequ10230SNP2
712.9 Sequ55107EST3
735.7 Sequ03235SNP1
767.7 Sequ14528EST3
775.1 Sequ07110EST2
790.0 Sequ28578EST3
806.2 Sequ05983SNP2
837.0 Sequ46238EST3
839.6 Sequ15305SNP3
871.3 Sequ0944TUF
893.0 Sequ14263EST3
920.6 Sequ45152EST3
938.6 Sequ37185EST3
980.2 Sequ20304EST3
1000.2 Sequ45232EST3
1011.9 Sequ11468EST3
1018.4 Sequ45078EST3
1132.1 Sequ0915TUF
Squ10M
0.0
33.4
55.6
204.3
215.4
271.2
395.9
440.5
451.6
462.7
485.0
496.1
Sequ08061SNP2
Sequ10500SNP2
Sequ21671SNP2
Sequ06622SNP2
Sequ22264SNP2
Sequ2159BAC
Sequ2587BAC
Sequ3009BAC
Sequ0922TUF
Sequ0452TUF
Sequ0611TUF
Sequ0165TUF
Sequ1178BAC
Sequ0840BAC
Sequ0882TUF
Sequ44970SNP3
Sequ55133SNP3
Sequ20814SNP2
Sequ07802SNP2
Sequ06325SNP2
Sequ1885BAC
Sequ1640BAC
Sequ0648BAC
Sequ0199BAC
Sequ0012BAC
Sequ0988TUF
Sequ0927TUF
Sequ0652TUF
Sequ0620TUF
Sequ0577TUF
Sequ0532TUF
Sequ0235TUF
Sequ0119TUF
Sequ16106SNP3
Sequ36093SNP3
Sequ36811SNP3
Sequ06042SNP2
Sequ07005SNP2
Sequ03235SNP1
Sequ04379SNP1
Sequ03717SNP1
Sequ2516BAC
Sequ1876BAC
Sequ1427BAC
Sequ1227BAC
Sequ0675BAC
Sequ0542BAC
Sequ1056TUF
Sequ0944TUF
Sequ0915TUF
Sequ0835TUF
Sequ0521TUF
Sequ0311TUF
Sequ0059TUF

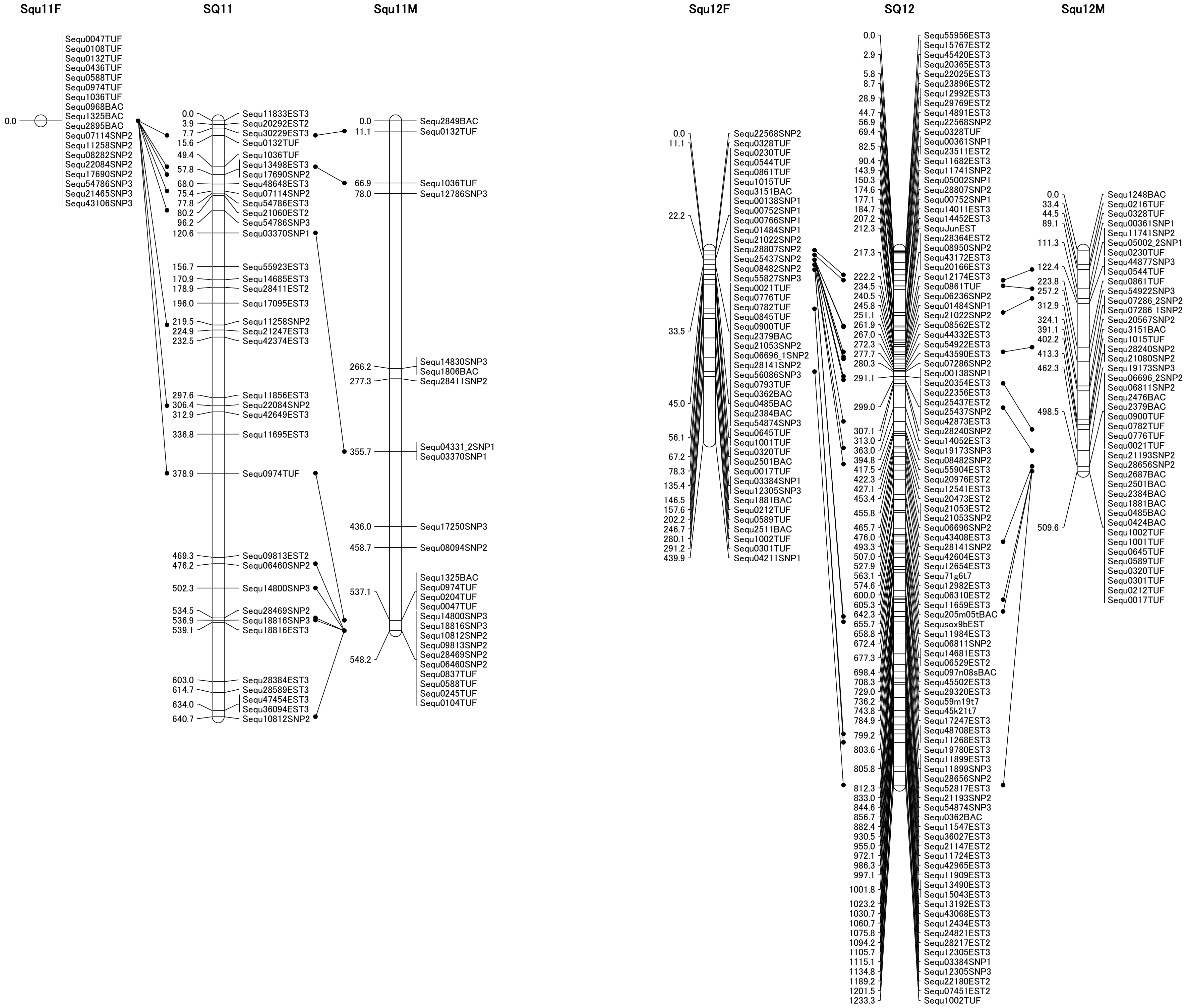
Squ11F
SQ11
Squ11M
Squ12F
SQ12
Squ12M
0.0
Sequ0047TUF
Sequ0108TUF
Sequ0132TUF
Sequ0436TUF
Sequ0588TUF
Sequ0974TUF
Sequ1036TUF
Sequ0968BAC
Sequ1325BAC
Sequ2895BAC
Sequ07114SNP2
Sequ11258SNP2
Sequ08282SNP2
Sequ22084SNP2
Sequ17690SNP2
Sequ54786SNP3
Sequ21465SNP3
Sequ43106SNP3
0.0 Sequ11833EST3
3.9 Sequ20292EST2
7.7 Sequ30229EST3
15.6 Sequ0132TUF
49.4 Sequ1036TUF
57.8 Sequ13498EST3
Sequ17690SNP2
68.0 Sequ48648EST3
75.4 Sequ07114SNP2
77.8 Sequ54786EST3
80.2 Sequ21060EST2
96.2 Sequ54786SNP3
120.6 Sequ03370SNP1
156.7 Sequ55923EST3
170.9 Sequ14685EST3
178.9 Sequ28411EST2
196.0 Sequ17095EST3
219.5 Sequ11258SNP2
224.9 Sequ21247EST3
232.5 Sequ42374EST3
297.6 Sequ11856EST3
306.4 Sequ22084SNP2
312.9 Sequ42649EST3
336.8 Sequ11695EST3
378.9 Sequ0974TUF
469.3 Sequ09813EST2
476.2 Sequ06460SNP2
502.3 Sequ14800SNP3
534.5 Sequ28469SNP2
536.9 Sequ18816SNP3
539.1 Sequ18816EST3
603.0 Sequ28384EST3
614.7 Sequ28589EST3
634.0 Sequ47454EST3
Sequ36094EST3
640.7 Sequ10812SNP2
0.0 Sequ2849BAC
11.1 Sequ0132TUF
66.9 Sequ1036TUF
78.0 Sequ12786SNP3
266.2 Sequ14830SNP3
Sequ1806BAC
277.3 Sequ28411SNP2
355.7 Sequ04331_2SNP1
Sequ03370SNP1
436.0 Sequ17250SNP3
458.7 Sequ08094SNP2
537.1
548.2
Sequ1325BAC
Sequ0974TUF
Sequ0204TUF
Sequ0047TUF
Sequ14800SNP3
Sequ18816SNP3
Sequ10812SNP2
Sequ09813SNP2
Sequ28469SNP2
Sequ06460SNP2
Sequ0837TUF
Sequ0588TUF
Sequ0245TUF
Sequ0104TUF
0.0
11.1
22.2
33.5
45.0
56.1
67.2
78.3
135.4
146.5
157.6
202.2
246.7
280.1
291.2
439.9
Sequ22568SNP2
Sequ0328TUF
Sequ0230TUF
Sequ0544TUF
Sequ0861TUF
Sequ1015TUF
Sequ3151BAC
Sequ00138SNP1
Sequ00752SNP1
Sequ00766SNP1
Sequ01484SNP1
Sequ21022SNP2
Sequ28807SNP2
Sequ25437SNP2
Sequ08482SNP2
Sequ55827SNP3
Sequ0021TUF
Sequ0776TUF
Sequ0782TUF
Sequ0845TUF
Sequ0900TUF
Sequ2379BAC
Sequ21053SNP2
Sequ06696_1SNP2
Sequ28141SNP2
Sequ56086SNP3
Sequ0793TUF
Sequ0362BAC
Sequ0485BAC
Sequ2384BAC
Sequ54874SNP3
Sequ0645TUF
Sequ1001TUF
Sequ0320TUF
Sequ2501BAC
Sequ0017TUF
Sequ03384SNP1
Sequ12305SNP3
Sequ1881BAC
Sequ0212TUF
Sequ0589TUF
Sequ2511BAC
Sequ1002TUF
Sequ0301TUF
Sequ04211SNP1
0.0
2.9
5.8
8.7
28.9
44.7
56.9
69.4
82.5
90.4
143.9
150.3
174.6
177.1
184.7
207.2
212.3
217.3
222.2
234.5
240.5
245.8
251.1
261.9
267.0
272.3
277.7
280.3
291.1
299.0
307.1
313.0
363.0
394.8
417.5
422.3
427.1
453.4
455.8
465.7
476.0
493.3
507.0
527.9
563.1
574.6
600.0
605.3
642.3
655.7
658.8
672.4
677.3
698.4
708.3
729.0
736.2
743.8
784.9
799.2
803.6
805.8
812.3
833.0
844.6
856.7
882.4
930.5
955.0
972.1
986.3
997.1
1001.8
1023.2
1030.7
1060.7
1075.8
1094.2
1105.7
1115.1
1134.8
1189.2
1201.5
1233.3
Sequ55956EST3
Sequ15767EST2
Sequ45420EST3
Sequ20365EST3
Sequ22025EST3
Sequ23896EST2
Sequ12992EST3
Sequ29769EST2
Sequ14891EST3
Sequ22568SNP2
Sequ0328TUF
Sequ00361SNP1
Sequ23511EST2
Sequ11682EST3
Sequ11741SNP2
Sequ05002SNP1
Sequ28807SNP2
Sequ00752SNP1
Sequ14011EST3
Sequ14452EST3
SequJunEST
Sequ28364EST2
Sequ08950SNP2
Sequ43172EST3
Sequ20166EST3
Sequ12174EST3
Sequ0861TUF
Sequ06236SNP2
Sequ01484SNP1
Sequ21022SNP2
Sequ08562EST2
Sequ44332EST3
Sequ54922EST3
Sequ43590EST3
Sequ07286SNP2
Sequ00138SNP1
Sequ20354EST3
Sequ22356EST3
Sequ25437EST2
Sequ25437SNP2
Sequ42873EST3
Sequ28240SNP2
Sequ14052EST3
Sequ19173SNP3
Sequ08482SNP2
Sequ55904EST3
Sequ20976EST2
Sequ12541EST3
Sequ20473EST2
Sequ21053EST2
Sequ21053SNP2
Sequ06696SNP2
Sequ43408EST3
Sequ28141SNP2
Sequ42604EST3
Sequ12654EST3
Sequ71g6t7
Sequ12982EST3
Sequ06310EST2
Sequ11659EST3
Sequ205m05tBAC
Sequsox9bEST
Sequ11984EST3
Sequ06811SNP2
Sequ14681EST3
Sequ06529EST2
Sequ097n08sBAC
Sequ45502EST3
Sequ29320EST3
Sequ59m19t7
Sequ45k21t7
Sequ17247EST3
Sequ48708EST3
Sequ11268EST3
Sequ19780EST3
Sequ11899EST3
Sequ11899SNP3
Sequ28656SNP2
Sequ52817EST3
Sequ21193SNP2
Sequ54874SNP3
Sequ0362BAC
Sequ11547EST3
Sequ36027EST3
Sequ21147EST2
Sequ11724EST3
Sequ42965EST3
Sequ11909EST3
Sequ13490EST3
Sequ15043EST3
Sequ13192EST3
Sequ43068EST3
Sequ12434EST3
Sequ24821EST3
Sequ28217EST2
Sequ12305EST3
Sequ03384SNP1
Sequ12305SNP3
Sequ22180EST2
Sequ07451EST2
Sequ1002TUF
0.0
33.4
44.5
89.1
111.3
122.4
223.8
257.2
312.9
324.1
391.1
402.2
413.3
462.3
498.5
509.6
Sequ1248BAC
Sequ0216TUF
Sequ0328TUF
Sequ00361SNP1
Sequ11741SNP2
Sequ05002_2SNP1
Sequ0230TUF
Sequ44877SNP3
Sequ0544TUF
Sequ0861TUF
Sequ54922SNP3
Sequ07286_2SNP2
Sequ07286_1SNP2
Sequ20567SNP2
Sequ3151BAC
Sequ1015TUF
Sequ28240SNP2
Sequ21080SNP2
Sequ19173SNP3
Sequ06696_2SNP2
Sequ06811SNP2
Sequ2476BAC
Sequ2379BAC
Sequ0900TUF
Sequ0782TUF
Sequ0776TUF
Sequ0021TUF
Sequ21193SNP2
Sequ28656SNP2
Sequ2687BAC
Sequ2501BAC
Sequ2384BAC
Sequ1881BAC
Sequ0485BAC
Sequ0424BAC
Sequ1002TUF
Sequ1001TUF
Sequ0645TUF
Sequ0589TUF
Sequ0320TUF
Sequ0301TUF
Sequ0212TUF
Sequ0017TUF

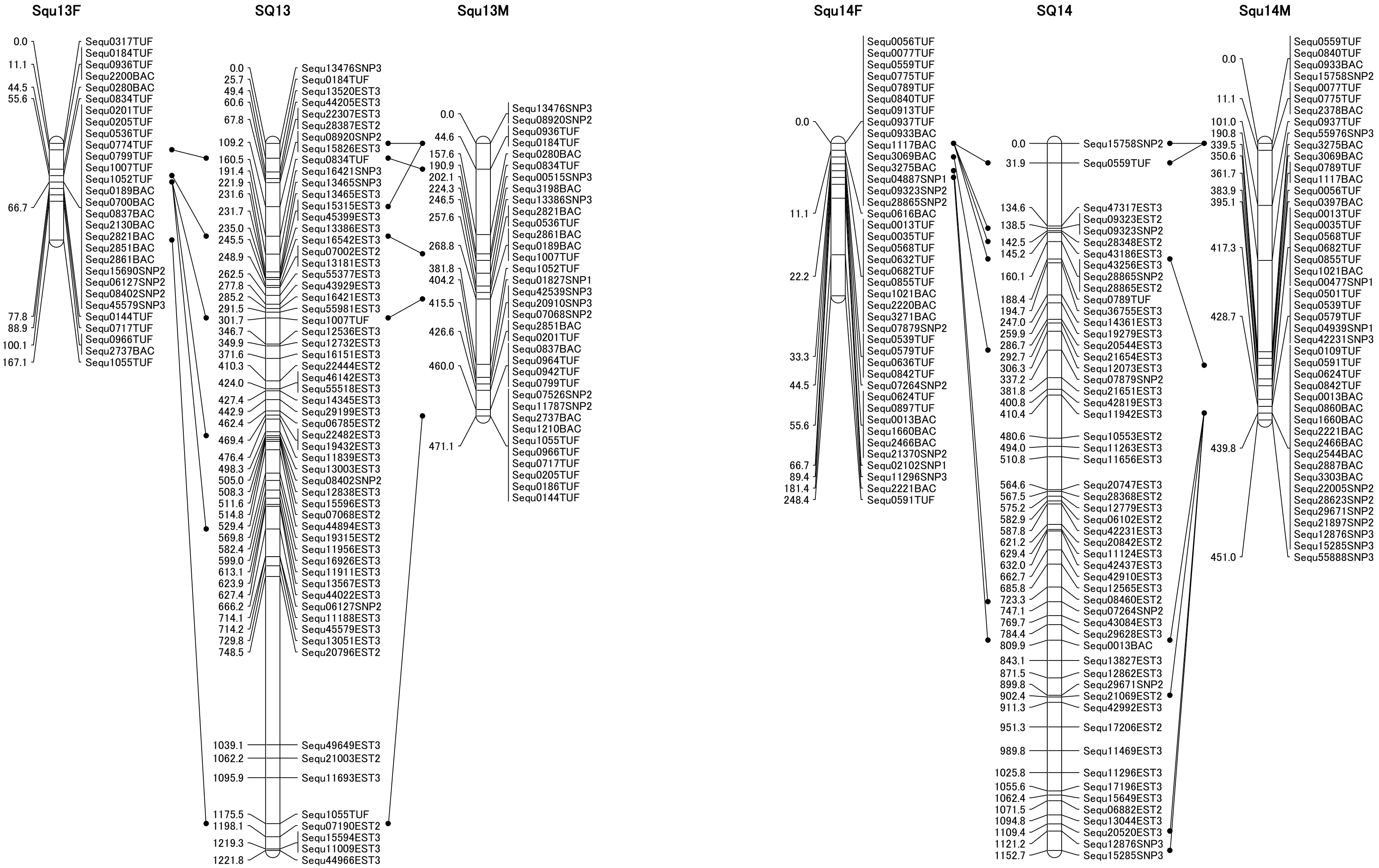

Squ13F
0.0
11.1
44.5
55.6
66.7
77.8
88.9
100.1
167.1
Sequ0317TUF
Sequ0184TUF
Sequ0936TUF
Sequ2200BAC
Sequ0280BAC
Sequ0834TUF
Sequ0201TUF
Sequ0205TUF
Sequ0536TUF
Sequ0774TUF
Sequ0799TUF
Sequ1007TUF
Sequ1052TUF
Sequ0189BAC
Sequ0700BAC
Sequ0837BAC
Sequ2130BAC
Sequ2821BAC
Sequ2851BAC
Sequ2861BAC
Sequ15690SNP2
Sequ06127SNP2
Sequ08402SNP2
Sequ45579SNP3
Sequ0144TUF
Sequ0717TUF
Sequ0966TUF
Sequ2737BAC
Sequ1055TUF
SQ13
0.0
25.7
49.4
60.6
67.8
109.2
160.5
191.4
221.9
231.6
231.7
235.0
245.5
248.9
262.5
277.8
285.2
291.5
301.7
346.7
349.9
371.6
410.3
424.0
427.4
442.9
462.4
469.4
476.4
498.3
505.0
508.3
511.6
514.8
529.4
569.8
582.4
599.0
613.1
623.9
627.4
666.2
714.1
714.2
729.8
748.5
1039.1
1062.2
1095.9
1175.5
1198.1
1219.3
1221.8
Sequ13476SNP3
Sequ0184TUF
Sequ13520EST3
Sequ44205EST3
Sequ22307EST3
Sequ28387EST2
Sequ08920SNP2
Sequ15826EST3
Sequ0834TUF
Sequ16421SNP3
Sequ13465SNP3
Sequ13465EST3
Sequ15315EST3
Sequ45399EST3
Sequ13386EST3
Sequ16542EST3
Sequ07002EST2
Sequ13181EST3
Sequ55377EST3
Sequ43929EST3
Sequ16421EST3
Sequ55981EST3
Sequ1007TUF
Sequ12536EST3
Sequ12732EST3
Sequ16151EST3
Sequ22444EST2
Sequ46142EST3
Sequ55518EST3
Sequ14345EST3
Sequ29199EST3
Sequ06785EST2
Sequ22482EST3
Sequ19432EST3
Sequ11839EST3
Sequ13003EST3
Sequ08402SNP2
Sequ12838EST3
Sequ15596EST3
Sequ07068EST2
Sequ44894EST3
Sequ19315EST2
Sequ11956EST3
Sequ16926EST3
Sequ11911EST3
Sequ13567EST3
Sequ44022EST3
Sequ06127SNP2
Sequ11188EST3
Sequ45579EST3
Sequ13051EST3
Sequ20796EST2
Sequ49649EST3
Sequ21003EST2
Sequ11693EST3
Sequ1055TUF
Sequ07190EST2
Sequ15594EST3
Sequ11009EST3
Sequ44966EST3
Squ13M
0.0
44.6
157.6
190.9
202.1
224.3
246.5
257.6
268.8
381.8
404.2
415.5
426.6
460.0
471.1
Sequ13476SNP3
Sequ08920SNP2
Sequ0936TUF
Sequ0184TUF
Sequ0280BAC
Sequ0834TUF
Sequ00515SNP3
Sequ3198BAC
Sequ13386SNP3
Sequ2821BAC
Sequ0536TUF
Sequ2861BAC
Sequ0189BAC
Sequ1007TUF
Sequ1052TUF
Sequ01827SNP1
Sequ42539SNP3
Sequ20910SNP3
Sequ07068SNP2
Sequ2851BAC
Sequ0201TUF
Sequ0837BAC
Sequ0964TUF
Sequ0942TUF
Sequ0799TUF
Sequ07526SNP2
Sequ11787SNP2
Sequ2737BAC
Sequ1210BAC
Sequ1055TUF
Sequ0966TUF
Sequ0717TUF
Sequ0205TUF
Sequ0186TUF
Sequ0144TUF
Squ14F
0.0
11.1
22.2
33.3
44.5
55.6
66.7
89.4
181.4
248.4
Sequ0056TUF
Sequ0077TUF
Sequ0559TUF
Sequ0775TUF
Sequ0789TUF
Sequ0840TUF
Sequ0913TUF
Sequ0937TUF
Sequ0933BAC
Sequ1117BAC
Sequ3069BAC
Sequ3275BAC
Sequ04887SNP1
Sequ09323SNP2
Sequ28865SNP2
Sequ0616BAC
Sequ0013TUF
Sequ0035TUF
Sequ0568TUF
Sequ0632TUF
Sequ0682TUF
Sequ0855TUF
Sequ1021BAC
Sequ2220BAC
Sequ3271BAC
Sequ07879SNP2
Sequ0539TUF
Sequ0579TUF
Sequ0636TUF
Sequ0842TUF
Sequ07264SNP2
Sequ0624TUF
Sequ0897TUF
Sequ0013BAC
Sequ1660BAC
Sequ2466BAC
Sequ21370SNP2
Sequ02102SNP1
Sequ11296SNP3
Sequ2221BAC
Sequ0591TUF
SQ14
0.0
31.9
134.6
138.5
142.5
145.2
160.1
188.4
194.7
247.0
259.9
286.7
292.7
306.3
337.2
381.8
400.8
410.4
480.6
494.0
510.8
564.6
567.5
575.2
582.9
587.8
621.2
629.4
632.0
662.7
685.8
723.3
747.1
769.7
784.4
809.9
843.1
871.5
899.8
902.4
911.3
951.3
989.8
1025.8
1055.6
1062.4
1071.5
1094.8
1109.4
1121.2
1152.7
Sequ15758SNP2
Sequ0559TUF
Sequ47317EST3
Sequ09323EST2
Sequ09323SNP2
Sequ28348EST2
Sequ43186EST3
Sequ43256EST3
Sequ28865SNP2
Sequ28865EST2
Sequ0789TUF
Sequ36755EST3
Sequ14361EST3
Sequ19279EST3
Sequ20544EST3
Sequ21654EST3
Sequ12073EST3
Sequ07879SNP2
Sequ21651EST3
Sequ42819EST3
Sequ11942EST3
Sequ10553EST2
Sequ11263EST3
Sequ11656EST3
Sequ20747EST3
Sequ28368EST2
Sequ12779EST3
Sequ06102EST2
Sequ42231EST3
Sequ20842EST2
Sequ11124EST3
Sequ42437EST3
Sequ42910EST3
Sequ12565EST3
Sequ08460EST2
Sequ07264SNP2
Sequ43084EST3
Sequ29628EST3
Sequ0013BAC
Sequ13827EST3
Sequ12862EST3
Sequ29671SNP2
Sequ21069EST2
Sequ42992EST3
Sequ17206EST2
Sequ11469EST3
Sequ11296EST3
Sequ17196EST3
Sequ15649EST3
Sequ06882EST2
Sequ13044EST3
Sequ20520EST3
Sequ12876SNP3
Sequ15285SNP3
Squ14M
0.0
11.1
101.0
190.8
339.5
350.6
361.7
383.9
395.1
417.3
428.7
439.8
451.0
Sequ0559TUF
Sequ0840TUF
Sequ0933BAC
Sequ15758SNP2
Sequ0077TUF
Sequ0775TUF
Sequ2378BAC
Sequ0937TUF
Sequ55976SNP3
Sequ3275BAC
Sequ3069BAC
Sequ0789TUF
Sequ1117BAC
Sequ0056TUF
Sequ0397BAC
Sequ0013TUF
Sequ0035TUF
Sequ0568TUF
Sequ0682TUF
Sequ0855TUF
Sequ1021BAC
Sequ00477SNP1
Sequ0501TUF
Sequ0539TUF
Sequ0579TUF
Sequ04939SNP1
Sequ42231SNP3
Sequ0109TUF
Sequ0591TUF
Sequ0624TUF
Sequ0842TUF
Sequ0013BAC
Sequ0860BAC
Sequ1660BAC
Sequ2221BAC
Sequ2466BAC
Sequ2544BAC
Sequ2887BAC
Sequ3303BAC
Sequ22005SNP2
Sequ28623SNP2
Sequ29671SNP2
Sequ21897SNP2
Sequ12876SNP3
Sequ15285SNP3
Sequ55888SNP3

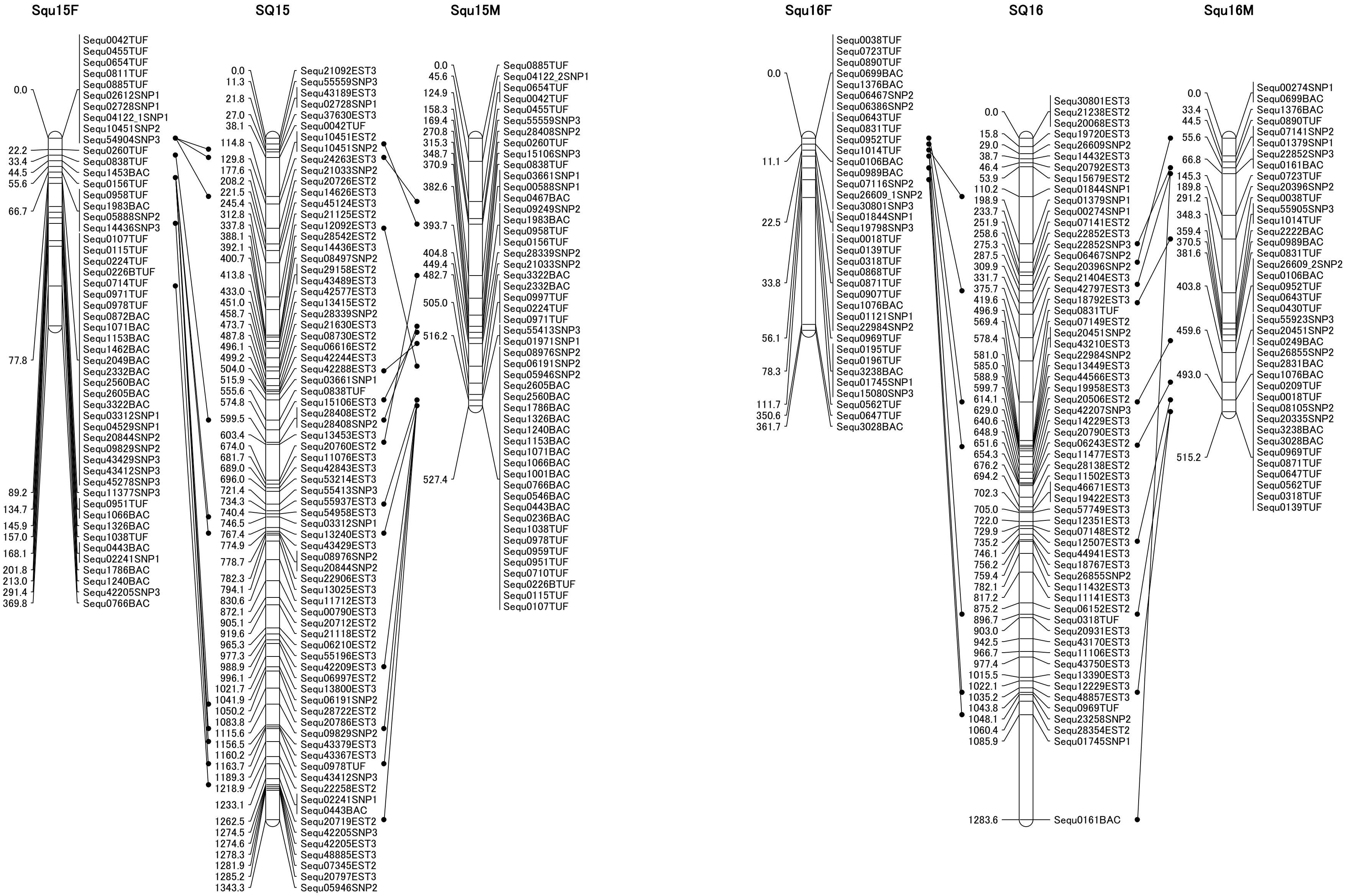
Squ15F
0.0
22.2
33.4
44.5
55.6
66.7
77.8
89.2
134.7
145.9
157.0
168.1
201.8
213.0
291.4
369.8
Sequ0042TUF
Sequ0455TUF
Sequ0654TUF
Sequ0811TUF
Sequ0885TUF
Sequ02612SNP1
Sequ02728SNP1
Sequ04122_1SNP1
Sequ10451SNP2
Sequ54904SNP3
Sequ0260TUF
Sequ0838TUF
Sequ1453BAC
Sequ0156TUF
Sequ0958TUF
Sequ1983BAC
Sequ05888SNP2
Sequ14436SNP3
Sequ0107TUF
Sequ0115TUF
Sequ0224TUF
Sequ0226BTUF
Sequ0714TUF
Sequ0971TUF
Sequ0978TUF
Sequ0872BAC
Sequ1071BAC
Sequ1153BAC
Sequ1462BAC
Sequ2049BAC
Sequ2332BAC
Sequ2560BAC
Sequ2605BAC
Sequ3322BAC
Sequ03312SNP1
Sequ04529SNP1
Sequ20844SNP2
Sequ09829SNP2
Sequ43429SNP3
Sequ43412SNP3
Sequ45278SNP3
Sequ11377SNP3
Sequ0951TUF
Sequ1066BAC
Sequ1326BAC
Sequ1038TUF
Sequ0443BAC
Sequ02241SNP1
Sequ1786BAC
Sequ1240BAC
Sequ42205SNP3
Sequ0766BAC
SQ15
0.0
11.3
21.8
27.0
38.1
114.8
129.8
177.6
208.2
221.5
245.4
312.8
337.8
388.1
392.1
400.7
413.8
433.0
451.0
458.7
473.7
487.8
496.1
499.2
504.0
515.9
555.6
574.8
599.5
603.4
674.0
681.7
689.0
696.0
721.4
734.3
740.4
746.5
767.4
774.9
778.7
782.3
794.1
830.6
872.1
905.1
919.6
965.3
977.3
988.9
996.1
1021.7
1041.9
1050.2
1083.8
1115.6
1156.5
1160.2
1163.7
1189.3
1218.9
1233.1
1262.5
1274.5
1274.6
1278.3
1281.9
1285.2
1343.3
Sequ21092EST3
Sequ55559SNP3
Sequ43189EST3
Sequ02728SNP1
Sequ37630EST3
Sequ0042TUF
Sequ10451EST2
Sequ10451SNP2
Sequ24263EST3
Sequ21033SNP2
Sequ20726EST2
Sequ14626EST3
Sequ45124EST3
Sequ21125EST2
Sequ12092EST3
Sequ28542EST2
Sequ14436SNP3
Sequ08497SNP2
Sequ29158EST2
Sequ43489EST3
Sequ42577EST3
Sequ13415EST2
Sequ28339SNP2
Sequ21630EST3
Sequ08730EST2
Sequ06616EST2
Sequ42244EST3
Sequ42288EST3
Sequ03661SNP1
Sequ0838TUF
Sequ15106EST3
Sequ28408EST2
Sequ28408SNP2
Sequ13453EST3
Sequ20760EST2
Sequ11076EST3
Sequ42843EST3
Sequ53214EST3
Sequ55413SNP3
Sequ55937EST3
Sequ54958EST3
Sequ03312SNP1
Sequ13240EST3
Sequ43429EST3
Sequ08976SNP2
Sequ20844SNP2
Sequ22906EST3
Sequ13025EST3
Sequ11712EST3
Sequ00790EST3
Sequ20712EST2
Sequ21118EST2
Sequ06210EST2
Sequ55196EST3
Sequ42209EST3
Sequ06997EST2
Sequ13800EST3
Sequ06191SNP2
Sequ28722EST2
Sequ20786EST3
Sequ09829SNP2
Sequ43379EST3
Sequ43367EST3
Sequ0978TUF
Sequ43412SNP3
Sequ22258EST2
Sequ02241SNP1
Sequ0443BAC
Sequ20719EST2
Sequ42205SNP3
Sequ42205EST3
Sequ48885EST3
Sequ07345EST2
Sequ20797EST3
Sequ05946SNP2
Squ15M
0.0
45.6
124.9
158.3
169.4
270.8
315.3
348.7
370.9
382.6
393.7
404.8
449.4
482.7
505.0
516.2
527.4
Sequ0885TUF
Sequ04122_2SNP1
Sequ0654TUF
Sequ0042TUF
Sequ0455TUF
Sequ55559SNP3
Sequ28408SNP2
Sequ0260TUF
Sequ15106SNP3
Sequ0838TUF
Sequ03661SNP1
Sequ00588SNP1
Sequ0467BAC
Sequ09249SNP2
Sequ1983BAC
Sequ0958TUF
Sequ0156TUF
Sequ28339SNP2
Sequ21033SNP2
Sequ3322BAC
Sequ2332BAC
Sequ0997TUF
Sequ0224TUF
Sequ0971TUF
Sequ55413SNP3
Sequ01971SNP1
Sequ08976SNP2
Sequ06191SNP2
Sequ05946SNP2
Sequ2605BAC
Sequ2560BAC
Sequ1786BAC
Sequ1326BAC
Sequ1240BAC
Sequ1153BAC
Sequ1071BAC
Sequ1066BAC
Sequ1001BAC
Sequ0766BAC
Sequ0546BAC
Sequ0443BAC
Sequ0236BAC
Sequ1038TUF
Sequ0978TUF
Sequ0959TUF
Sequ0951TUF
Sequ0710TUF
Sequ0226BTUF
Sequ0115TUF
Sequ0107TUF
Squ16F
0.0
11.1
22.5
33.8
56.1
78.3
111.7
350.6
361.7
Sequ0038TUF
Sequ0723TUF
Sequ0890TUF
Sequ0699BAC
Sequ1376BAC
Sequ06467SNP2
Sequ06386SNP2
Sequ0643TUF
Sequ0831TUF
Sequ0952TUF
Sequ1014TUF
Sequ0106BAC
Sequ0989BAC
Sequ07116SNP2
Sequ26609_1SNP2
Sequ30801SNP3
Sequ01844SNP1
Sequ19798SNP3
Sequ0018TUF
Sequ0139TUF
Sequ0318TUF
Sequ0868TUF
Sequ0871TUF
Sequ0907TUF
Sequ1076BAC
Sequ01121SNP1
Sequ22984SNP2
Sequ0969TUF
Sequ0195TUF
Sequ0196TUF
Sequ3238BAC
Sequ01745SNP1
Sequ15080SNP3
Sequ0562TUF
Sequ0647TUF
Sequ3028BAC
SQ16
0.0
15.8
29.0
38.7
46.4
53.9
110.2
198.9
233.7
251.9
258.6
275.3
287.5
309.9
331.7
375.7
419.6
496.9
569.4
578.4
581.0
585.0
588.9
599.7
614.1
629.0
640.6
648.9
651.6
654.3
676.2
694.2
702.3
705.0
722.0
729.9
735.2
746.1
756.2
759.4
782.1
817.2
875.2
896.7
903.0
942.5
966.7
977.4
1015.5
1022.1
1035.2
1043.8
1048.1
1060.4
1085.9
1283.6
Sequ30801EST3
Sequ21238EST2
Sequ20068EST3
Sequ19720EST3
Sequ26609SNP2
Sequ14432EST3
Sequ20792EST3
Sequ15679EST2
Sequ01844SNP1
Sequ01379SNP1
Sequ00274SNP1
Sequ07141EST2
Sequ22852EST3
Sequ22852SNP3
Sequ06467SNP2
Sequ20396SNP2
Sequ21404EST3
Sequ42797EST3
Sequ18792EST3
Sequ0831TUF
Sequ07149EST2
Sequ20451SNP2
Sequ43210EST3
Sequ22984SNP2
Sequ13449EST3
Sequ44566EST3
Sequ19958EST3
Sequ20506EST2
Sequ42207SNP3
Sequ14229EST3
Sequ20790EST3
Sequ06243EST2
Sequ11477EST3
Sequ28138EST2
Sequ11502EST3
Sequ46671EST3
Sequ19422EST3
Sequ57749EST3
Sequ12351EST3
Sequ07148EST2
Sequ12507EST3
Sequ44941EST3
Sequ18767EST3
Sequ26855SNP2
Sequ11432EST3
Sequ11141EST3
Sequ06152EST2
Sequ0318TUF
Sequ20931EST3
Sequ43170EST3
Sequ11106EST3
Sequ43750EST3
Sequ13390EST3
Sequ12229EST3
Sequ48857EST3
Sequ0969TUF
Sequ23258SNP2
Sequ28354EST2
Sequ01745SNP1
Sequ0161BAC
Squ16M
0.0
33.4
44.5
55.6
66.8
145.3
189.8
291.2
348.3
359.4
370.5
381.6
403.8
459.6
493.0
515.2
Sequ00274SNP1
Sequ0699BAC
Sequ1376BAC
Sequ0890TUF
Sequ07141SNP2
Sequ01379SNP1
Sequ22852SNP3
Sequ0161BAC
Sequ0723TUF
Sequ20396SNP2
Sequ0038TUF
Sequ55905SNP3
Sequ1014TUF
Sequ2222BAC
Sequ0989BAC
Sequ0831TUF
Sequ26609_2SNP2
Sequ0106BAC
Sequ0952TUF
Sequ0643TUF
Sequ0430TUF
Sequ55923SNP3
Sequ20451SNP2
Sequ0249BAC
Sequ26855SNP2
Sequ2831BAC
Sequ1076BAC
Sequ0209TUF
Sequ0018TUF
Sequ08105SNP2
Sequ20335SNP2
Sequ3238BAC
Sequ3028BAC
Sequ0969TUF
Sequ0871TUF
Sequ0647TUF
Sequ0562TUF
Sequ0318TUF
Sequ0139TUF

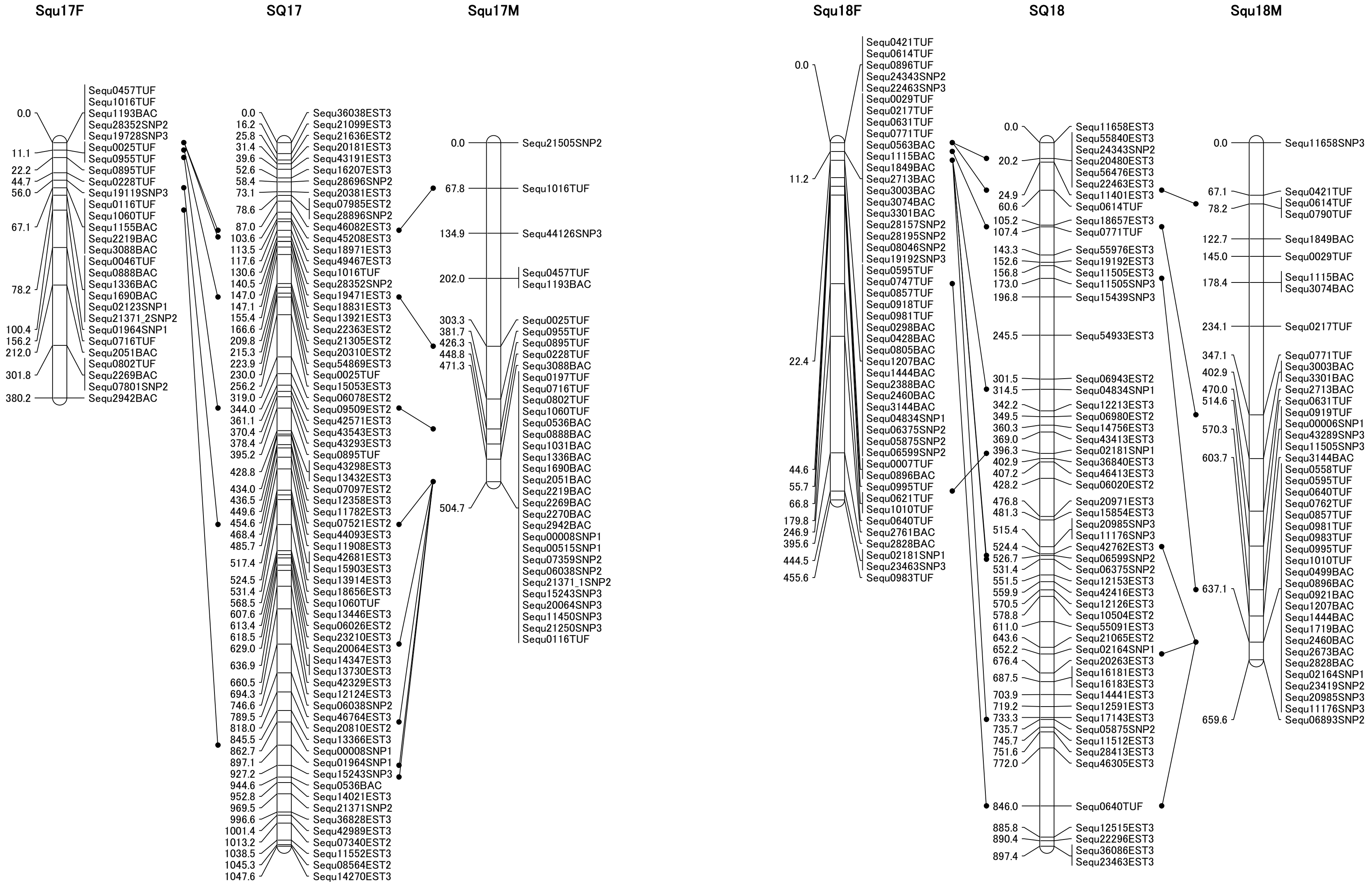
Squ17F
0.0
11.1
22.2
44.7
56.0
67.1
78.2
100.4
156.2
212.0
301.8
380.2
Sequ0457TUF
Sequ1016TUF
Sequ1193BAC
Sequ28352SNP2
Sequ19728SNP3
Sequ0025TUF
Sequ0955TUF
Sequ0895TUF
Sequ0228TUF
Sequ19119SNP3
Sequ0116TUF
Sequ1060TUF
Sequ1155BAC
Sequ2219BAC
Sequ3088BAC
Sequ0046TUF
Sequ0888BAC
Sequ1336BAC
Sequ1690BAC
Sequ02123SNP1
Sequ21371_2SNP2
Sequ01964SNP1
Sequ0716TUF
Sequ2051BAC
Sequ0802TUF
Sequ2269BAC
Sequ07801SNP2
Sequ2942BAC
SQ17
0.0
16.2
25.8
31.4
39.6
52.6
58.4
73.1
78.6
87.0
103.6
113.5
117.6
130.6
140.5
147.0
147.1
155.4
166.6
209.8
215.3
223.9
230.0
256.2
319.0
344.0
361.1
370.4
378.4
395.2
428.8
434.0
436.5
449.6
454.6
468.4
485.7
517.4
524.5
531.4
568.5
607.6
613.4
618.5
629.0
636.9
660.5
694.3
746.6
789.5
818.0
845.5
862.7
897.1
927.2
944.6
952.8
969.5
996.6
1001.4
1013.2
1038.5
1045.3
1047.6
Sequ36038EST3
Sequ21099EST3
Sequ21636EST2
Sequ20181EST3
Sequ43191EST3
Sequ16207EST3
Sequ28696SNP2
Sequ20381EST3
Sequ07985EST2
Sequ28896SNP2
Sequ46082EST3
Sequ45208EST3
Sequ18971EST3
Sequ49467EST3
Sequ1016TUF
Sequ28352SNP2
Sequ19471EST3
Sequ18831EST3
Sequ13921EST3
Sequ22363EST2
Sequ21305EST2
Sequ20310EST2
Sequ54869EST3
Sequ0025TUF
Sequ15053EST3
Sequ06078EST2
Sequ09509EST2
Sequ42571EST3
Sequ43543EST3
Sequ43293EST3
Sequ0895TUF
Sequ43298EST3
Sequ13432EST3
Sequ07097EST2
Sequ12358EST3
Sequ11782EST3
Sequ07521EST2
Sequ44093EST3
Sequ11908EST3
Sequ42681EST3
Sequ15903EST3
Sequ13914EST3
Sequ18656EST3
Sequ1060TUF
Sequ13446EST3
Sequ06026EST2
Sequ23210EST3
Sequ20064EST3
Sequ14347EST3
Sequ13730EST3
Sequ42329EST3
Sequ12124EST3
Sequ06038SNP2
Sequ46764EST3
Sequ20810EST2
Sequ13366EST3
Sequ00008SNP1
Sequ01964SNP1
Sequ15243SNP3
Sequ0536BAC
Sequ14021EST3
Sequ21371SNP2
Sequ36828EST3
Sequ42989EST3
Sequ07340EST2
Sequ11552EST3
Sequ08564EST2
Sequ14270EST3
Squ17M
0.0
67.8
134.9
202.0
303.3
381.7
426.3
448.8
471.3
504.7
Sequ21505SNP2
Sequ1016TUF
Sequ44126SNP3
Sequ0457TUF
Sequ1193BAC
Sequ0025TUF
Sequ0955TUF
Sequ0895TUF
Sequ0228TUF
Sequ3088BAC
Sequ0197TUF
Sequ0716TUF
Sequ0802TUF
Sequ1060TUF
Sequ0536BAC
Sequ0888BAC
Sequ1031BAC
Sequ1336BAC
Sequ1690BAC
Sequ2051BAC
Sequ2219BAC
Sequ2269BAC
Sequ2270BAC
Sequ2942BAC
Sequ00008SNP1
Sequ00515SNP1
Sequ07359SNP2
Sequ06038SNP2
Sequ21371_1SNP2
Sequ15243SNP3
Sequ20064SNP3
Sequ11450SNP3
Sequ21250SNP3
Sequ0116TUF
Squ18F
0.0
11.2
22.4
44.6
55.7
66.8
179.8
246.9
395.6
444.5
455.6
Sequ0421TUF
Sequ0614TUF
Sequ0896TUF
Sequ24343SNP2
Sequ22463SNP3
Sequ0029TUF
Sequ0217TUF
Sequ0631TUF
Sequ0771TUF
Sequ0563BAC
Sequ1115BAC
Sequ1849BAC
Sequ2713BAC
Sequ3003BAC
Sequ3074BAC
Sequ3301BAC
Sequ28157SNP2
Sequ28195SNP2
Sequ08046SNP2
Sequ19192SNP3
Sequ0595TUF
Sequ0747TUF
Sequ0857TUF
Sequ0918TUF
Sequ0981TUF
Sequ0298BAC
Sequ0428BAC
Sequ0805BAC
Sequ1207BAC
Sequ1444BAC
Sequ2388BAC
Sequ2460BAC
Sequ3144BAC
Sequ04834SNP1
Sequ06375SNP2
Sequ05875SNP2
Sequ06599SNP2
Sequ0007TUF
Sequ0896BAC
Sequ0995TUF
Sequ0621TUF
Sequ1010TUF
Sequ0640TUF
Sequ2761BAC
Sequ2828BAC
Sequ02181SNP1
Sequ23463SNP3
Sequ0983TUF
SQ18
0.0
20.2
24.9
60.6
105.2
107.4
143.3
152.6
156.8
173.0
196.8
245.5
301.5
314.5
342.2
349.5
360.3
369.0
396.3
402.9
407.2
428.2
476.8
481.3
515.4
524.4
526.7
531.4
551.5
559.9
570.5
578.8
611.0
643.6
652.2
676.4
687.5
703.9
719.2
733.3
735.7
745.7
751.6
772.0
846.0
885.8
890.4
897.4
Sequ11658EST3
Sequ55840EST3
Sequ24343SNP2
Sequ20480EST3
Sequ56476EST3
Sequ22463EST3
Sequ11401EST3
Sequ0614TUF
Sequ18657EST3
Sequ0771TUF
Sequ55976EST3
Sequ19192EST3
Sequ11505EST3
Sequ11505SNP3
Sequ15439SNP3
Sequ54933EST3
Sequ06943EST2
Sequ04834SNP1
Sequ12213EST3
Sequ06980EST2
Sequ14756EST3
Sequ43413EST3
Sequ02181SNP1
Sequ36840EST3
Sequ46413EST3
Sequ06020EST2
Sequ20971EST3
Sequ15854EST3
Sequ20985SNP3
Sequ11176SNP3
Sequ42762EST3
Sequ06599SNP2
Sequ06375SNP2
Sequ12153EST3
Sequ42416EST3
Sequ12126EST3
Sequ10504EST2
Sequ55091EST3
Sequ21065EST2
Sequ02164SNP1
Sequ20263EST3
Sequ16181EST3
Sequ16183EST3
Sequ14441EST3
Sequ12591EST3
Sequ17143EST3
Sequ05875SNP2
Sequ11512EST3
Sequ28413EST3
Sequ46305EST3
Sequ0640TUF
Sequ12515EST3
Sequ22296EST3
Sequ36086EST3
Sequ23463EST3
Squ18M
0.0
67.1
78.2
122.7
145.0
178.4
234.1
347.1
402.9
470.0
514.6
570.3
603.7
637.1
659.6
Sequ11658SNP3
Sequ0421TUF
Sequ0614TUF
Sequ0790TUF
Sequ1849BAC
Sequ0029TUF
Sequ1115BAC
Sequ3074BAC
Sequ0217TUF
Sequ0771TUF
Sequ3003BAC
Sequ3301BAC
Sequ2713BAC
Sequ0631TUF
Sequ0919TUF
Sequ00006SNP1
Sequ43289SNP3
Sequ11505SNP3
Sequ3144BAC
Sequ0558TUF
Sequ0595TUF
Sequ0640TUF
Sequ0762TUF
Sequ0857TUF
Sequ0981TUF
Sequ0983TUF
Sequ0995TUF
Sequ1010TUF
Sequ0499BAC
Sequ0896BAC
Sequ0921BAC
Sequ1207BAC
Sequ1444BAC
Sequ1719BAC
Sequ2460BAC
Sequ2673BAC
Sequ2828BAC
Sequ02164SNP1
Sequ23419SNP2
Sequ20985SNP3
Sequ11176SNP3
Sequ06893SNP2

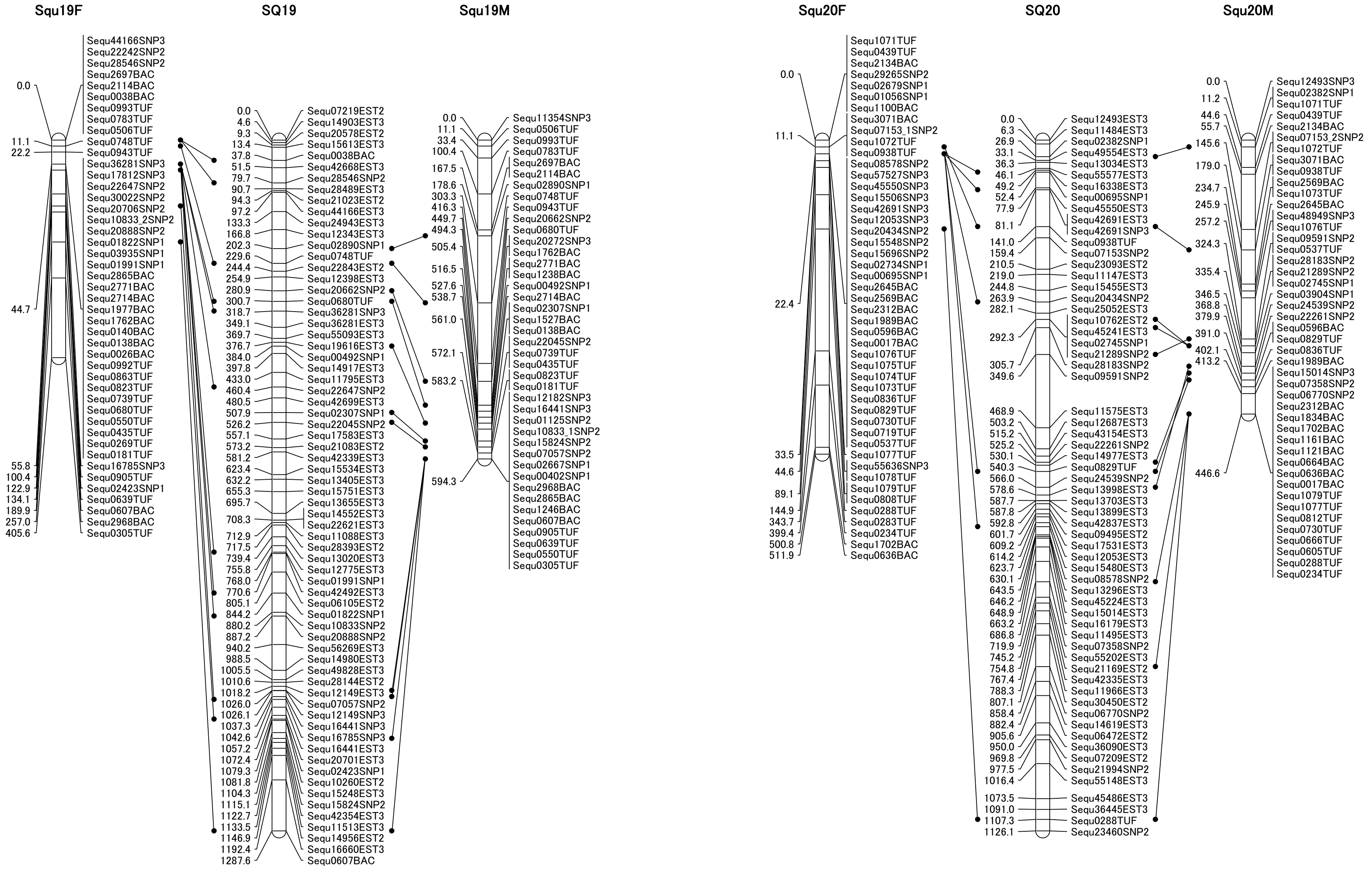
Squ19F
0.0
11.1
22.2
44.7
55.8
100.4
122.9
134.1
189.9
257.0
405.6
Sequ44166SNP3
Sequ22242SNP2
Sequ28546SNP2
Sequ2697BAC
Sequ2114BAC
Sequ0038BAC
Sequ0993TUF
Sequ0783TUF
Sequ0506TUF
Sequ0748TUF
Sequ0943TUF
Sequ36281SNP3
Sequ17812SNP3
Sequ22647SNP2
Sequ30022SNP2
Sequ20706SNP2
Sequ10833_2SNP2
Sequ20888SNP2
Sequ01822SNP1
Sequ03935SNP1
Sequ01991SNP1
Sequ2865BAC
Sequ2771BAC
Sequ2714BAC
Sequ1977BAC
Sequ1762BAC
Sequ0140BAC
Sequ0138BAC
Sequ0026BAC
Sequ0992TUF
Sequ0863TUF
Sequ0823TUF
Sequ0739TUF
Sequ0680TUF
Sequ0550TUF
Sequ0435TUF
Sequ0269TUF
Sequ0181TUF
Sequ16785SNP3
Sequ0905TUF
Sequ02423SNP1
Sequ0639TUF
Sequ0607BAC
Sequ2968BAC
Sequ0305TUF
SQ19
0.0 Sequ07219EST2
4.6 Sequ14903EST3
9.3 Sequ20578EST2
13.4 Sequ15613EST3
37.8 Sequ0038BAC
51.5 Sequ42668EST3
79.7 Sequ28546SNP2
90.7 Sequ28489EST3
94.3 Sequ21023EST2
97.2 Sequ44166EST3
133.3 Sequ24943EST3
166.8 Sequ12343EST3
202.3 Sequ02890SNP1
229.6 Sequ0748TUF
244.4 Sequ22843EST2
254.9 Sequ12398EST3
280.9 Sequ20662SNP2
300.7 Sequ0680TUF
318.7 Sequ36281SNP3
349.1 Sequ36281EST3
369.7 Sequ55093EST3
376.7 Sequ19616EST3
384.0 Sequ00492SNP1
397.8 Sequ14917EST3
433.0 Sequ11795EST3
460.4 Sequ22647SNP2
480.5 Sequ42699EST3
507.9 Sequ02307SNP1
526.2 Sequ22045SNP2
557.1 Sequ17583EST3
573.2 Sequ21083EST2
581.2 Sequ42339EST3
623.4 Sequ15534EST3
632.2 Sequ13405EST3
655.3 Sequ15751EST3
695.7 Sequ13655EST3
708.3 Sequ14552EST3
Sequ22621EST3
712.9 Sequ11088EST3
717.5 Sequ28393EST2
739.4 Sequ13020EST3
755.8 Sequ12775EST3
768.0 Sequ01991SNP1
770.6 Sequ42492EST3
805.1 Sequ06105EST2
844.2 Sequ01822SNP1
880.2 Sequ10833SNP2
887.2 Sequ20888SNP2
940.2 Sequ56269EST3
988.5 Sequ14980EST3
1005.5 Sequ49828EST3
1010.6 Sequ28144EST2
1018.2 Sequ12149EST3
1026.0 Sequ07057SNP2
1026.1 Sequ12149SNP3
1037.3 Sequ16441SNP3
1042.6 Sequ16785SNP3
1057.2 Sequ16441EST3
1072.4 Sequ20701EST3
1079.3 Sequ02423SNP1
1081.8 Sequ10260EST2
1104.3 Sequ15248EST3
1115.1 Sequ15824SNP2
1122.7 Sequ42354EST3
1133.5 Sequ11513EST3
1146.9 Sequ14956EST2
1192.4 Sequ16660EST3
1287.6 Sequ0607BAC
Squ19M
0.0
11.1
33.4
100.4
167.5
178.6
303.3
416.3
449.7
494.3
505.4
516.5
527.6
538.7
561.0
572.1
583.2
594.3
Sequ11354SNP3
Sequ0506TUF
Sequ0993TUF
Sequ0783TUF
Sequ2697BAC
Sequ2114BAC
Sequ02890SNP1
Sequ0748TUF
Sequ0943TUF
Sequ20662SNP2
Sequ0680TUF
Sequ20272SNP3
Sequ1762BAC
Sequ2771BAC
Sequ1238BAC
Sequ00492SNP1
Sequ2714BAC
Sequ02307SNP1
Sequ1527BAC
Sequ0138BAC
Sequ22045SNP2
Sequ0739TUF
Sequ0435TUF
Sequ0823TUF
Sequ0181TUF
Sequ12182SNP3
Sequ16441SNP3
Sequ01125SNP2
Sequ10833_1SNP2
Sequ15824SNP2
Sequ07057SNP2
Sequ02667SNP1
Sequ00402SNP1
Sequ2968BAC
Sequ2865BAC
Sequ1246BAC
Sequ0607BAC
Sequ0905TUF
Sequ0639TUF
Sequ0550TUF
Sequ0305TUF
Squ20F
0.0
11.1
22.4
33.5
44.6
89.1
144.9
343.7
399.4
500.8
511.9
Sequ1071TUF
Sequ0439TUF
Sequ2134BAC
Sequ29265SNP2
Sequ02679SNP1
Sequ01056SNP1
Sequ1100BAC
Sequ3071BAC
Sequ07153_1SNP2
Sequ1072TUF
Sequ0938TUF
Sequ08578SNP2
Sequ57527SNP3
Sequ45550SNP3
Sequ15506SNP3
Sequ42691SNP3
Sequ12053SNP3
Sequ20434SNP2
Sequ15548SNP2
Sequ15696SNP2
Sequ02734SNP1
Sequ00695SNP1
Sequ2645BAC
Sequ2569BAC
Sequ2312BAC
Sequ1989BAC
Sequ0596BAC
Sequ0017BAC
Sequ1076TUF
Sequ1075TUF
Sequ1074TUF
Sequ1073TUF
Sequ0836TUF
Sequ0829TUF
Sequ0730TUF
Sequ0719TUF
Sequ0537TUF
Sequ1077TUF
Sequ55636SNP3
Sequ1078TUF
Sequ1079TUF
Sequ0808TUF
Sequ0288TUF
Sequ0283TUF
Sequ0234TUF
Sequ1702BAC
Sequ0636BAC
SQ20
0.0 Sequ12493EST3
6.3 Sequ11484EST3
26.9 Sequ02382SNP1
33.1 Sequ49554EST3
36.3 Sequ13034EST3
46.1 Sequ55577EST3
49.2 Sequ16338EST3
52.4 Sequ00695SNP1
77.9 Sequ45550EST3
81.1 Sequ42691EST3
Sequ42691SNP3
141.0 Sequ0938TUF
159.4 Sequ07153SNP2
210.5 Sequ23093EST2
219.0 Sequ11147EST3
244.8 Sequ15455EST3
263.9 Sequ20434SNP2
282.1 Sequ25052EST3
Sequ10762EST2
Sequ45241EST3
292.3 Sequ02745SNP1
Sequ21289SNP2
305.7 Sequ28183SNP2
349.6 Sequ09591SNP2
468.9 Sequ11575EST3
503.2 Sequ12687EST3
515.2 Sequ43154EST3
525.2 Sequ22261SNP2
530.1 Sequ14977EST3
540.3 Sequ0829TUF
566.0 Sequ24539SNP2
578.6 Sequ13998EST3
587.7 Sequ13703EST3
587.8 Sequ13899EST3
592.8 Sequ42837EST3
601.7 Sequ09495EST2
609.2 Sequ17531EST3
614.2 Sequ12053EST3
623.7 Sequ15480EST3
630.1 Sequ08578SNP2
643.5 Sequ13296EST3
646.2 Sequ45224EST3
648.9 Sequ15014EST3
663.2 Sequ16179EST3
686.8 Sequ11495EST3
719.9 Sequ07358SNP2
745.2 Sequ55202EST3
754.8 Sequ21169EST2
767.4 Sequ42335EST3
788.3 Sequ11966EST3
807.1 Sequ30450EST2
858.4 Sequ06770SNP2
882.4 Sequ14619EST3
905.6 Sequ06472EST2
950.0 Sequ36090EST3
969.8 Sequ07209EST2
977.5 Sequ21994SNP2
1016.4 Sequ55148EST3
1073.5 Sequ45486EST3
1091.0 Sequ36445EST3
1107.3 Sequ0288TUF
1126.1 Sequ23460SNP2
Squ20M
0.0
11.2
44.6
55.7
145.6
179.0
234.7
245.9
257.2
324.3
335.4
346.5
368.8
379.9
391.0
402.1
413.2
446.6
Sequ12493SNP3
Sequ02382SNP1
Sequ1071TUF
Sequ0439TUF
Sequ2134BAC
Sequ07153_2SNP2
Sequ1072TUF
Sequ3071BAC
Sequ0938TUF
Sequ2569BAC
Sequ1073TUF
Sequ2645BAC
Sequ48949SNP3
Sequ1076TUF
Sequ09591SNP2
Sequ0537TUF
Sequ28183SNP2
Sequ21289SNP2
Sequ02745SNP1
Sequ03904SNP1
Sequ24539SNP2
Sequ22261SNP2
Sequ0596BAC
Sequ0829TUF
Sequ0836TUF
Sequ1989BAC
Sequ15014SNP3
Sequ07358SNP2
Sequ06770SNP2
Sequ2312BAC
Sequ1834BAC
Sequ1702BAC
Sequ1161BAC
Sequ1121BAC
Sequ0664BAC
Sequ0636BAC
Sequ0017BAC
Sequ1079TUF
Sequ1077TUF
Sequ0812TUF
Sequ0730TUF
Sequ0666TUF
Sequ0605TUF
Sequ0288TUF
Sequ0234TUF

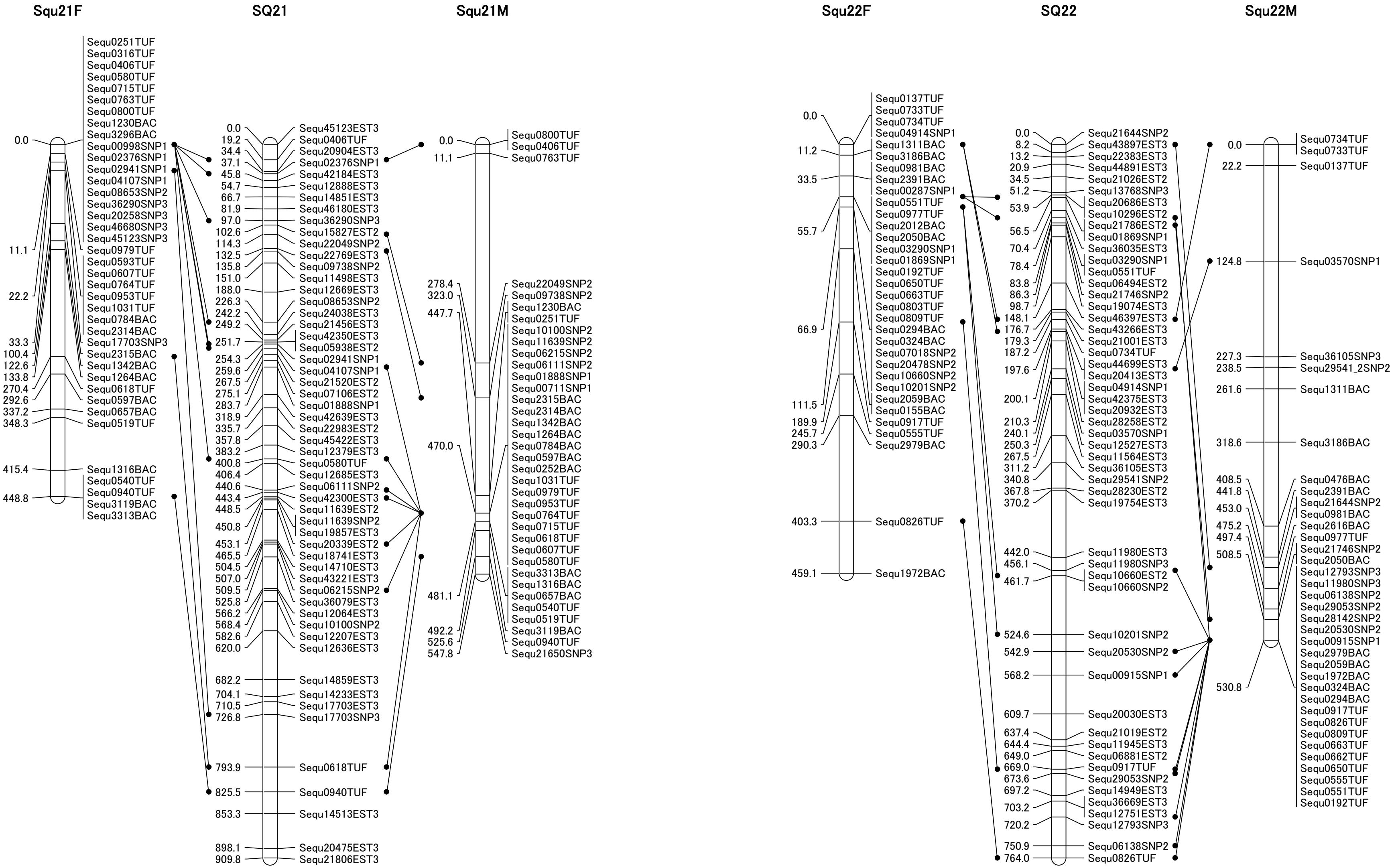

Squ21F
0.0
11.1
22.2
33.3
100.4
122.6
133.8
270.4
292.6
337.2
348.3
415.4
448.8
Sequ0251TUF
Sequ0316TUF
Sequ0406TUF
Sequ0580TUF
Sequ0715TUF
Sequ0763TUF
Sequ0800TUF
Sequ1230BAC
Sequ3296BAC
Sequ00998SNP1
Sequ02376SNP1
Sequ02941SNP1
Sequ04107SNP1
Sequ08653SNP2
Sequ36290SNP3
Sequ20258SNP3
Sequ46680SNP3
Sequ45123SNP3
Sequ0979TUF
Sequ0593TUF
Sequ0607TUF
Sequ0764TUF
Sequ0953TUF
Sequ1031TUF
Sequ0784BAC
Sequ2314BAC
Sequ17703SNP3
Sequ2315BAC
Sequ1342BAC
Sequ1264BAC
Sequ0618TUF
Sequ0597BAC
Sequ0657BAC
Sequ0519TUF
Sequ1316BAC
Sequ0540TUF
Sequ0940TUF
Sequ3119BAC
Sequ3313BAC
SQ21
0.0 Sequ45123EST3
19.2 Sequ0406TUF
34.4 Sequ20904EST3
37.1 Sequ02376SNP1
45.8 Sequ42184EST3
54.7 Sequ12888EST3
66.7 Sequ14851EST3
81.9 Sequ46180EST3
97.0 Sequ36290SNP3
102.6 Sequ15827EST2
114.3 Sequ22049SNP2
132.5 Sequ22769EST3
135.8 Sequ09738SNP2
151.0 Sequ11498EST3
188.0 Sequ12669EST3
226.3 Sequ08653SNP2
242.2 Sequ24038EST3
249.2 Sequ21456EST3
251.7 Sequ42350EST3
Sequ05938EST2
254.3 Sequ02941SNP1
259.6 Sequ04107SNP1
267.5 Sequ21520EST2
275.1 Sequ07106EST2
283.7 Sequ01888SNP1
318.9 Sequ42639EST3
335.7 Sequ22983EST2
357.8 Sequ45422EST3
383.2 Sequ12379EST3
400.8 Sequ0580TUF
406.4 Sequ12685EST3
440.6 Sequ06111SNP2
443.4 Sequ42300EST3
448.5 Sequ11639EST2
450.8 Sequ11639SNP2
Sequ19857EST3
453.1 Sequ20339EST2
465.5 Sequ18741EST3
504.5 Sequ14710EST3
507.0 Sequ43221EST3
509.5 Sequ06215SNP2
525.8 Sequ36079EST3
566.2 Sequ12064EST3
568.4 Sequ10100SNP2
582.6 Sequ12207EST3
620.0 Sequ12636EST3
682.2 Sequ14859EST3
704.1 Sequ14233EST3
710.5 Sequ17703EST3
726.8 Sequ17703SNP3
793.9 Sequ0618TUF
825.5 Sequ0940TUF
853.3 Sequ14513EST3
898.1 Sequ20475EST3
909.8 Sequ21806EST3
Squ21M
0.0
11.1
278.4
323.0
447.7
470.0
481.1
492.2
525.6
547.8
Sequ0800TUF
Sequ0406TUF
Sequ0763TUF
Sequ22049SNP2
Sequ09738SNP2
Sequ1230BAC
Sequ0251TUF
Sequ10100SNP2
Sequ11639SNP2
Sequ06215SNP2
Sequ06111SNP2
Sequ01888SNP1
Sequ00711SNP1
Sequ2315BAC
Sequ2314BAC
Sequ1342BAC
Sequ1264BAC
Sequ0784BAC
Sequ0597BAC
Sequ0252BAC
Sequ1031TUF
Sequ0979TUF
Sequ0953TUF
Sequ0764TUF
Sequ0715TUF
Sequ0618TUF
Sequ0607TUF
Sequ0580TUF
Sequ3313BAC
Sequ1316BAC
Sequ0657BAC
Sequ0540TUF
Sequ0519TUF
Sequ3119BAC
Sequ0940TUF
Sequ21650SNP3
Squ22F
0.0
11.2
33.5
55.7
66.9
111.5
189.9
245.7
290.3
403.3
459.1
Sequ0137TUF
Sequ0733TUF
Sequ0734TUF
Sequ04914SNP1
Sequ1311BAC
Sequ3186BAC
Sequ0981BAC
Sequ2391BAC
Sequ00287SNP1
Sequ0551TUF
Sequ0977TUF
Sequ2012BAC
Sequ2050BAC
Sequ03290SNP1
Sequ01869SNP1
Sequ0192TUF
Sequ0650TUF
Sequ0663TUF
Sequ0803TUF
Sequ0809TUF
Sequ0294BAC
Sequ0324BAC
Sequ07018SNP2
Sequ20478SNP2
Sequ10660SNP2
Sequ10201SNP2
Sequ2059BAC
Sequ0155BAC
Sequ0917TUF
Sequ0555TUF
Sequ2979BAC
Sequ0826TUF
Sequ1972BAC
SQ22
0.0 Sequ21644SNP2
8.2 Sequ43897EST3
13.2 Sequ22383EST3
20.9 Sequ44891EST3
34.5 Sequ21026EST2
51.2 Sequ13768SNP3
53.9 Sequ20686EST3
Sequ10296EST2
56.5 Sequ21786EST2
Sequ01869SNP1
70.4 Sequ36035EST3
78.4 Sequ03290SNP1
Sequ0551TUF
83.8 Sequ06494EST2
86.3 Sequ21746SNP2
98.7 Sequ19074EST3
148.1 Sequ46397EST3
176.7 Sequ43266EST3
179.3 Sequ21001EST3
187.2 Sequ0734TUF
197.6 Sequ44699EST3
Sequ20413EST3
Sequ04914SNP1
200.1 Sequ42375EST3
Sequ20932EST3
210.3 Sequ28258EST2
240.1 Sequ03570SNP1
250.3 Sequ12527EST3
267.5 Sequ11564EST3
311.2 Sequ36105EST3
340.8 Sequ29541SNP2
367.8 Sequ28230EST2
370.2 Sequ19754EST3
442.0 Sequ11980EST3
456.1 Sequ11980SNP3
461.7 Sequ10660EST2
Sequ10660SNP2
524.6 Sequ10201SNP2
542.9 Sequ20530SNP2
568.2 Sequ00915SNP1
609.7 Sequ20030EST3
637.4 Sequ21019EST2
644.4 Sequ11945EST3
649.0 Sequ06881EST2
669.0 Sequ0917TUF
673.6 Sequ29053SNP2
697.2 Sequ14949EST3
703.2 Sequ36669EST3
Sequ12751EST3
720.2 Sequ12793SNP3
750.9 Sequ06138SNP2
764.0 Sequ0826TUF
Squ22M
0.0 Sequ0734TUF
Sequ0733TUF
22.2 Sequ0137TUF
124.8 Sequ03570SNP1
227.3 Sequ36105SNP3
238.5 Sequ29541_2SNP2
261.6 Sequ1311BAC
318.6 Sequ3186BAC
408.5
441.8
453.0
475.2
497.4
508.5
530.8
Sequ0476BAC
Sequ2391BAC
Sequ21644SNP2
Sequ0981BAC
Sequ2616BAC
Sequ0977TUF
Sequ21746SNP2
Sequ2050BAC
Sequ12793SNP3
Sequ11980SNP3
Sequ06138SNP2
Sequ29053SNP2
Sequ28142SNP2
Sequ20530SNP2
Sequ00915SNP1
Sequ2979BAC
Sequ2059BAC
Sequ1972BAC
Sequ0324BAC
Sequ0294BAC
Sequ0917TUF
Sequ0826TUF
Sequ0809TUF
Sequ0663TUF
Sequ0662TUF
Sequ0650TUF
Sequ0555TUF
Sequ0551TUF
Sequ0192TUF

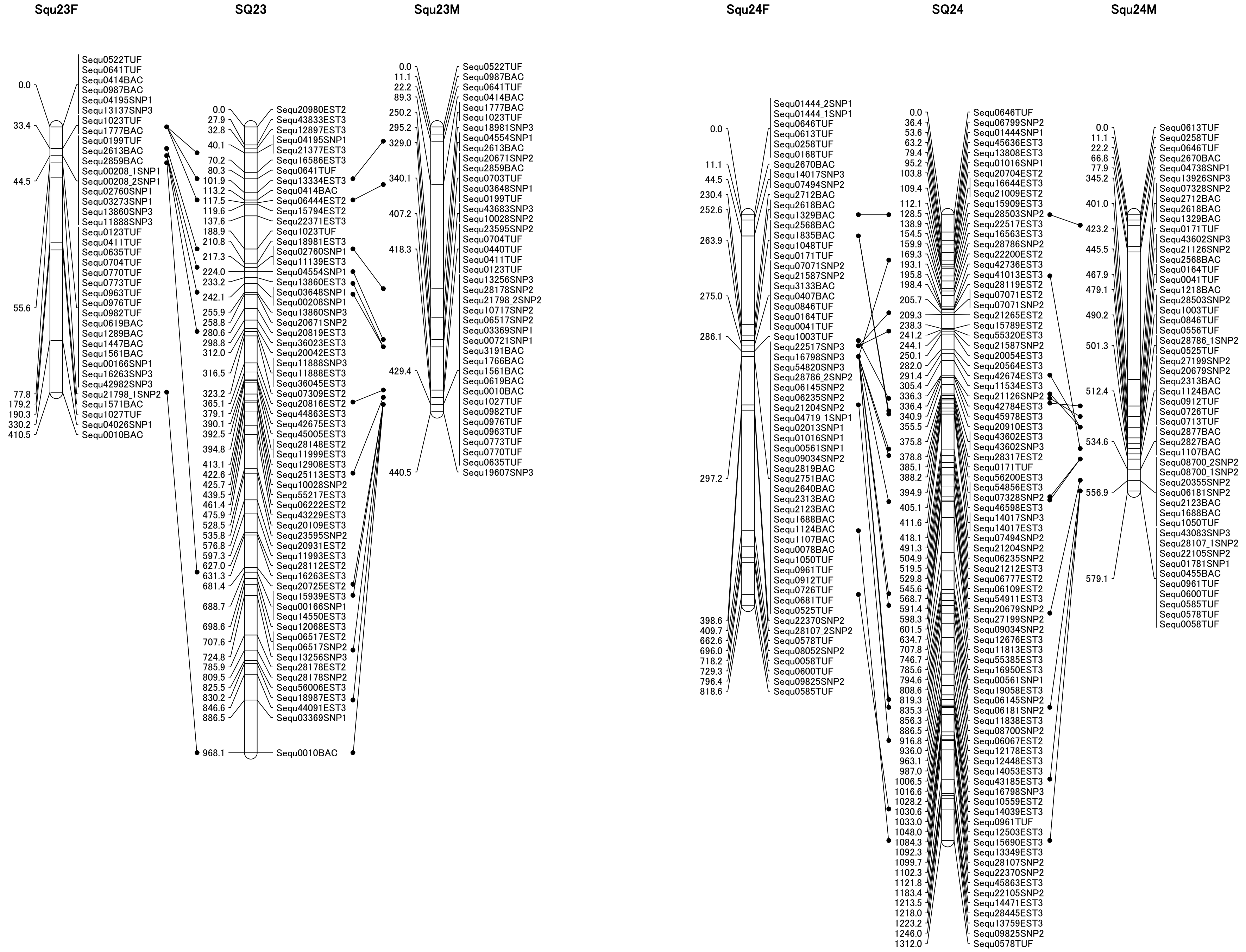

Squ23F
SQ23
Squ23M
Squ24F
SQ24
Squ24M
0.0
33.4
44.5
55.6
77.8
179.2
190.3
330.2
410.5
Sequ0522TUF
Sequ0641TUF
Sequ0414BAC
Sequ0987BAC
Sequ04195SNP1
Sequ13137SNP3
Sequ1023TUF
Sequ1777BAC
Sequ0199TUF
Sequ2613BAC
Sequ2859BAC
Sequ00208_1SNP1
Sequ00208_2SNP1
Sequ02760SNP1
Sequ03273SNP1
Sequ13860SNP3
Sequ11888SNP3
Sequ0123TUF
Sequ0411TUF
Sequ0635TUF
Sequ0704TUF
Sequ0770TUF
Sequ0773TUF
Sequ0963TUF
Sequ0976TUF
Sequ0982TUF
Sequ0619BAC
Sequ1289BAC
Sequ1447BAC
Sequ1561BAC
Sequ00166SNP1
Sequ16263SNP3
Sequ42982SNP3
Sequ21798_1SNP2
Sequ1571BAC
Sequ1027TUF
Sequ04026SNP1
Sequ0010BAC
0.0
27.9
32.8
40.1
70.2
80.3
101.9
113.2
117.5
119.6
137.6
188.9
210.8
217.3
224.0
233.2
242.1
255.9
258.8
280.6
298.8
312.0
316.5
323.2
365.1
379.1
390.1
392.5
394.8
413.1
422.6
425.7
439.5
461.4
475.9
528.5
535.8
576.8
597.3
627.0
631.3
681.4
688.7
698.6
707.6
724.8
785.9
809.5
825.5
830.2
846.6
886.5
968.1
Sequ20980EST2
Sequ43833EST3
Sequ12897EST3
Sequ04195SNP1
Sequ21377EST3
Sequ16586EST3
Sequ0641TUF
Sequ13334EST3
Sequ0414BAC
Sequ06444EST2
Sequ15794EST2
Sequ22371EST3
Sequ1023TUF
Sequ18981EST3
Sequ02760SNP1
Sequ11139EST3
Sequ04554SNP1
Sequ13860EST3
Sequ03648SNP1
Sequ00208SNP1
Sequ13860SNP3
Sequ20671SNP2
Sequ20819EST3
Sequ36023EST3
Sequ20042EST3
Sequ11888SNP3
Sequ11888EST3
Sequ36045EST3
Sequ07309EST2
Sequ20816EST2
Sequ44863EST3
Sequ42675EST3
Sequ45005EST3
Sequ28148EST2
Sequ11999EST3
Sequ12908EST3
Sequ25113EST3
Sequ10028SNP2
Sequ55217EST3
Sequ06222EST2
Sequ43229EST3
Sequ20109EST3
Sequ23595SNP2
Sequ20931EST2
Sequ11993EST3
Sequ28112EST2
Sequ16263EST3
Sequ20725EST2
Sequ15939EST3
Sequ00166SNP1
Sequ14550EST3
Sequ12068EST3
Sequ06517EST2
Sequ06517SNP2
Sequ13256SNP3
Sequ28178EST2
Sequ28178SNP2
Sequ56006EST3
Sequ18987EST3
Sequ44091EST3
Sequ03369SNP1
Sequ0010BAC
0.0
11.1
22.2
89.3
250.2
295.2
329.0
340.1
407.2
418.3
429.4
440.5
Sequ0522TUF
Sequ0987BAC
Sequ0641TUF
Sequ0414BAC
Sequ1777BAC
Sequ1023TUF
Sequ18981SNP3
Sequ04554SNP1
Sequ2613BAC
Sequ20671SNP2
Sequ2859BAC
Sequ0703TUF
Sequ03648SNP1
Sequ0199TUF
Sequ43683SNP3
Sequ10028SNP2
Sequ23595SNP2
Sequ0704TUF
Sequ0440TUF
Sequ0411TUF
Sequ0123TUF
Sequ13256SNP3
Sequ28178SNP2
Sequ21798_2SNP2
Sequ10717SNP2
Sequ06517SNP2
Sequ03369SNP1
Sequ00721SNP1
Sequ3191BAC
Sequ1766BAC
Sequ1561BAC
Sequ0619BAC
Sequ0010BAC
Sequ1027TUF
Sequ0982TUF
Sequ0976TUF
Sequ0963TUF
Sequ0773TUF
Sequ0770TUF
Sequ0635TUF
Sequ19607SNP3
0.0
11.1
44.5
230.4
252.6
263.9
275.0
286.1
297.2
398.6
409.7
662.6
696.0
718.2
729.3
796.4
818.6
Sequ01444_2SNP1
Sequ01444_1SNP1
Sequ0646TUF
Sequ0613TUF
Sequ0258TUF
Sequ0168TUF
Sequ2670BAC
Sequ14017SNP3
Sequ07494SNP2
Sequ2712BAC
Sequ2618BAC
Sequ1329BAC
Sequ2568BAC
Sequ1835BAC
Sequ1048TUF
Sequ0171TUF
Sequ07071SNP2
Sequ21587SNP2
Sequ3133BAC
Sequ0407BAC
Sequ0846TUF
Sequ0164TUF
Sequ0041TUF
Sequ1003TUF
Sequ22517SNP3
Sequ16798SNP3
Sequ54820SNP3
Sequ28786_2SNP2
Sequ06145SNP2
Sequ06235SNP2
Sequ21204SNP2
Sequ04719_1SNP1
Sequ02013SNP1
Sequ01016SNP1
Sequ00561SNP1
Sequ09034SNP2
Sequ2819BAC
Sequ2751BAC
Sequ2640BAC
Sequ2313BAC
Sequ1688BAC
Sequ1124BAC
Sequ1107BAC
Sequ0078BAC
Sequ1050TUF
Sequ0961TUF
Sequ0912TUF
Sequ0726TUF
Sequ0681TUF
Sequ0525TUF
Sequ22370SNP2
Sequ28107_2SNP2
Sequ0578TUF
Sequ08052SNP2
Sequ0058TUF
Sequ0600TUF
Sequ09825SNP2
Sequ0585TUF
0.0
36.4
53.6
63.2
79.4
95.2
103.8
109.4
112.1
128.5
138.9
154.5
159.9
169.3
193.1
195.8
198.4
205.7
209.3
238.3
241.2
244.1
250.1
282.0
291.4
305.4
336.3
336.4
340.9
355.5
375.8
378.8
385.1
388.2
394.9
405.1
411.6
418.1
491.3
504.9
519.5
529.8
545.6
568.7
591.4
598.3
601.5
634.7
707.8
746.7
785.6
794.6
808.6
819.3
835.3
856.3
886.5
916.8
936.0
963.1
987.0
1006.5
1016.6
1028.2
1030.6
1033.0
1048.0
1084.3
1092.3
1099.7
1102.3
1121.8
1183.4
1213.5
1218.0
1223.2
1246.0
1312.0
Sequ0646TUF
Sequ06799SNP2
Sequ01444SNP1
Sequ45636EST3
Sequ13808EST3
Sequ01016SNP1
Sequ20704EST2
Sequ16644EST3
Sequ21009EST2
Sequ15909EST3
Sequ28503SNP2
Sequ22517EST3
Sequ16563EST3
Sequ28786SNP2
Sequ22200EST2
Sequ42736EST3
Sequ41013EST3
Sequ28119EST2
Sequ07071EST2
Sequ07071SNP2
Sequ21265EST2
Sequ15789EST2
Sequ55320EST3
Sequ21587SNP2
Sequ20054EST3
Sequ20564EST3
Sequ42674EST3
Sequ11534EST3
Sequ21126SNP2
Sequ42784EST3
Sequ45978EST3
Sequ20910EST3
Sequ43602EST3
Sequ43602SNP3
Sequ28317EST2
Sequ0171TUF
Sequ56200EST3
Sequ54856EST3
Sequ07328SNP2
Sequ46598EST3
Sequ14017SNP3
Sequ14017EST3
Sequ07494SNP2
Sequ21204SNP2
Sequ06235SNP2
Sequ21212EST3
Sequ06777EST2
Sequ06109EST2
Sequ54911EST3
Sequ20679SNP2
Sequ27199SNP2
Sequ09034SNP2
Sequ12676EST3
Sequ11813EST3
Sequ55385EST3
Sequ16950EST3
Sequ00561SNP1
Sequ19058EST3
Sequ06145SNP2
Sequ06181SNP2
Sequ11838EST3
Sequ08700SNP2
Sequ06067EST2
Sequ12178EST3
Sequ12448EST3
Sequ14053EST3
Sequ43185EST3
Sequ16798SNP3
Sequ10559EST2
Sequ14039EST3
Sequ0961TUF
Sequ12503EST3
Sequ15690EST3
Sequ13349EST3
Sequ28107SNP2
Sequ22370SNP2
Sequ45863EST3
Sequ22105SNP2
Sequ14471EST3
Sequ28445EST3
Sequ13759EST3
Sequ09825SNP2
Sequ0578TUF
0.0
11.1
22.2
66.8
77.9
345.2
401.0
423.2
445.5
467.9
479.1
490.2
501.3
512.4
534.6
556.9
579.1
Sequ0613TUF
Sequ0258TUF
Sequ0646TUF
Sequ2670BAC
Sequ04738SNP1
Sequ13926SNP3
Sequ07328SNP2
Sequ2712BAC
Sequ2618BAC
Sequ1329BAC
Sequ0171TUF
Sequ43602SNP3
Sequ21126SNP2
Sequ2568BAC
Sequ0164TUF
Sequ0041TUF
Sequ1218BAC
Sequ28503SNP2
Sequ1003TUF
Sequ0846TUF
Sequ0556TUF
Sequ28786_1SNP2
Sequ0525TUF
Sequ27199SNP2
Sequ20679SNP2
Sequ2313BAC
Sequ1124BAC
Sequ0912TUF
Sequ0726TUF
Sequ0713TUF
Sequ2877BAC
Sequ2827BAC
Sequ1107BAC
Sequ08700_2SNP2
Sequ08700_1SNP2
Sequ20355SNP2
Sequ06181SNP2
Sequ2123BAC
Sequ1688BAC
Sequ1050TUF
Sequ43083SNP3
Sequ28107_1SNP2
Sequ22105SNP2
Sequ01781SNP1
Sequ0455BAC
Sequ0961TUF
Sequ0600TUF
Sequ0585TUF
Sequ0578TUF
Sequ0058TUF
